# Constituents of the Stem Bark of *Symphonia globulifera* Linn. f. with Antileishmanial and Antibacterial Activities

**DOI:** 10.3390/molecules28062473

**Published:** 2023-03-08

**Authors:** Ruland Tchuinkeu Nguengang, Billy Toussie Tchegnitegni, Eric Carly Nono Nono, Georges Bellier Tabekoueng, Yannick Stéphane Fotsing Fongang, Jean Jules Kezetas Bankeu, Jean Rodolphe Chouna, Céline Nguefeu Nkenfou, Fabrice Boyom Fekam, Norbert Sewald, Bruno Ndjakou Lenta

**Affiliations:** 1 Department of Organic Chemistry, Faculty of Science, University of Yaoundé I, Yaoundé P.O. Box 812, Cameroon; 2 Department of Chemistry, Higher Teacher Training College, University of Yaoundé I, Yaoundé P.O. Box 47, Cameroon; 3 Department of Chemistry, Faculty of Science, University of Dschang, Dschang P.O. Box 67, Cameroon; 4 Department of Chemistry, Faculty of Science, University of Douala, Douala P.O. Box 24157, Cameroon; 5 Department of Chemistry, Higher Teachers’ Training College, The University of Maroua, Maroua P.O. Box 55, Cameroon; 6 Department of Chemistry, Faculty of Science, The University of Bamenda, Bambili P.O. Box 39, Cameroon; 7 Department of Biology, Higher Teacher Training College, University of Yaoundé I, Yaoundé P.O. Box 47, Cameroon; 8 Antimicrobial & Biocontrol Agents Unit, Laboratory for Phytobiochemistry and Medicinal Plants Studies, Department of Biochemistry, University of Yaoundé I, Yaoundé P.O. Box 812, Cameroon; 9 Organic and Bioorganic Chemistry, Faculty of Chemistry, Bielefeld University, D-33501 Bielefeld, Germany

**Keywords:** Clusiaceae, *Symphonia globulifera*, polyprenylated benzophenones, guttiferone, tocotrienol, cytotoxicity, antileishmanial, antibacterial

## Abstract

The chemical investigation of the *n*-hexane fraction from the methanol extract of the stem bark of *Symphonia globulifera* Linn f., which displayed good in vitro activity against *Leishmania donovani* NR-48822 promastigotes (IC_50_ 43.11 µg/mL), led to the isolation of three previously unreported polyprenylated benzophenones, guttiferone U (**1**), V (**2**)/W (**3**), and a new tocotrienol derivative named globuliferanol (**4**), along with 11 known compounds (**5**–**15**). Their structures were elucidated based on their NMR and MS data. Some isolated compounds were assessed for both their antileishmanial and cytotoxic activities against *L. donovani* and Vero cells, respectively. Guttiferone K (**5**) exhibited the best potency (IC_50_ 3.30 μg/mL), but with low selectivity to Vero cells. The *n*-hexane fraction and some compounds were also assessed in vitro for their antibacterial activity against seven bacterial strains. All the samples exhibited moderate to potent antibacterial activity (MICs ≤ 15.6 µg/mL) against at least one of the tested strains.

## 1. Introduction

Leishmaniasis is a neglected infectious vector-borne disease caused by the protozoan parasite *Leishmania,* transmitted to humans and animals through the bite of infected female phlebotomine sandflies during their blood meals [1,2]. Visceral leishmaniasis remains the most lethal if left untreated, with the most severe and underreported form of the disease representing up to 95% of cases [3,4,5,6]. Based on the most recent available data on this disease, between 498,000 and 862,000 new cases of all forms of leishmaniasis occur annually, resulting in up to 18,700 deaths and approximately 1.6 million disability-adjusted life years lost [7]. Moreover, the number of imported infection cases in non-endemic areas is increasing [8,9,10]. Leishmaniasis is, however, largely ignored and faces problems of resistance of the parasite to the available therapeutic molecules. The goal of the WHO 2021–2030 neglected tropical diseases road map is to reduce the mortality caused by the disease to less than 1% [11]. Treatment of leishmaniasis is based on chemotherapy, but only drugs such as amphotericin B, pentavalent antimony derivatives, pentamidine, miltefosine, and paramomycin are available. Therefore, there is an urgent need to develop alternative drugs with less side effects that could be more efficient as effective treatments [5,12]. Cameroonian medicinal plants from the Clusiaceae family are reported to be a source of potent antileishmanial compounds [13,14,15,16]. The Clusiaceae family possesses approximately 1200 species grouped into about 50 genera. *Symphonia* is a genus of tropical woody plants, especially tall trees with milky or colored sap, and includes 17 species distributed from South America to Africa and Madagascar [17]. *S. globulifera* Linn f. is a medium to tall tree (of over 30 m) with a characteristic yellow latex broadly distributed across the Neotropics and Equatorial Africa. It is the only species found outside of Madagascar Island where palaeobotanical studies have established its origin [17,18]. Its parts are used in African and South American folk medicines to treat diabetes, stomach troubles, cough, malaria, intestinal worms, jaundice, fever, and scabies [19,20,21]. In Cameroon, its bark and heartwood are used as laxatives for pregnant women and as general tonics [22]. In Panama, its fresh latex is used as a cataplasm against skin diseases and body pain [23]. The decoction of the bark is traditionally rubbed on the skin for the treatment of cutaneous leishmaniasis in Colombia [24]. Previous chemical investigations of *S. globulifera* led to the isolation of bioactive biflavonoids, steroids, xanthones, benzophenones, and polyprenylated acylphloroglucinol (PPAPs) [25,26,27]. In our continuing search for potent antileishmanial agents from Cameroonian medicinal plants, the *n*-hexane, EtOAc, and *n*-BuOH soluble fractions from the methanol extract of the stem bark of *S. globulifera* were assessed. Herein, we report the antileishmanial bioguided isolation of the constituents of the active *n*-hexane soluble fraction along with the antibacterial activities of some isolated compounds against seven bacterial strains.

## 2. Results and Discussion

### 2.1. Structure Elucidation

**Figure 1:**
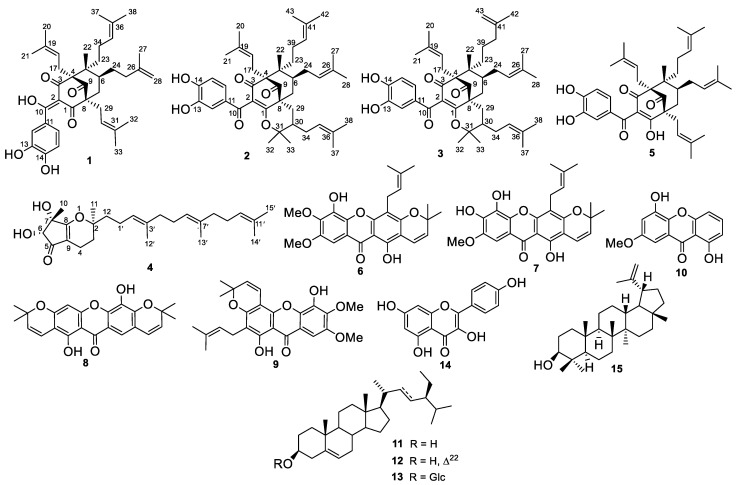
Structures of isolated compounds **1**–**15**.

The MeOH extract of the stem bark of *S. globulifera* was partitioned into three fractions by liquid–liquid partition using *n*-hexane, EtOAc, and *n*-BuOH. The in vitro antileishmanial assay was performed on the crude extract and fractions against the *L. donovani* NR-48822 promastigote strain. The MeOH extract, the EtOAc, and *n*-BuOH fractions were inactive (IC_50_ value > 100 μg/mL), while the *n*-hexane fraction showed good antileishmanial activity with an IC_50_ value of 43.11 μg/mL. The *n*-hexane fraction was further chemically investigated to give 15 compounds including three previously unreported guttiferone analogs, guttiferone U (**1**), a mixture of guttiferones V/W (**2/3**), one new tocotrienol derivative, globuliferanol (**4**) together with guttiferone K (**5**) [28], gaboxanthone (**6**) [29], xanthone V2 (**7**) [30], pyranojacareubin (**8**) [31], symphonin (**9**) [32], 1,5-dihydroxy-3-methoxyxanthone (**10**) [33], *β*-sitosterol (**11**), stigmasterol (**12**) [34], *β*-sitosterol 3-*O*-*β*-D-glucopyranoside (**13**) [34], kaempferol (**14**) [35], and lupeol (**15)** [36] (Figure 1). 

Compound **1** was obtained as a yellow amorphous solid. It was optically active with ${\left[\alpha \right]}_{589}^{20}\;$+ 93 (*c* 0.5, MeOH). Its molecular formula, C_38_H_50_O_6_, with 14 degrees of unsaturation was deduced from its positive HR-ESIMS, which exhibited a sodium adduct peak [M  +  Na]^+^ at *m/z* 625.3494 (calcd for C_38_H_50_O_6_Na^+^, 625.3500) and was later confirmed by its NMR data. The ^1^H and ^13^C NMR data of **1** (Table 1) were very similar to those of guttiferone K (**5**) [28]. Its ^1^H NMR spectrum exhibited signals of an ABX pattern at ẟ_H_ 6.81 (1H, d, *J* = 8.3 Hz, H-15), 7.06 (1H, dd, *J* = 8.3, 2.1 Hz, H-16), and 7.23 (1H, d, *J* = 2.1 Hz, H-12). The proton spectrum also displayed characteristic signals of three isopent-2-enyl groups at ẟ_H_ {[2.05 (2H, m, H-29), 5.06 (1H, m, H-30), 1.65 (3H, s, H-32), and 1.53 (3H, s, H-33)], [2.68 (2H, m, H-17), 5.15 (1H, m, H-18), 1.71 (3H, s, H-20), and 1.65 (3H, s, H-21)], [1.91 (2H, m, H-34), 5.11 (1H, m, H-35), 1.68 (3H, s, H-37), and 1.60 (3H, s, H-38)]}, and those of one isopent-3-enyl unit at ẟ_H_ [2.05 (2H, m, H-24), 1.91 (2H, m, H-25), 1.70 (3H, s, H-27), and 4.66 (2H, brs, H-28)]. 

**Figure 2:**
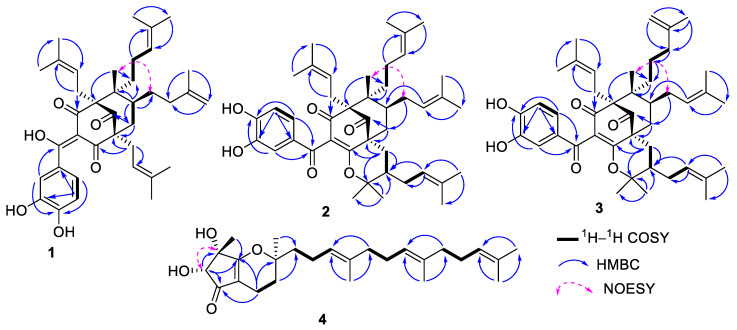
Key ^1^H–^1^H COSY, NOESY, and HMBC correlations of compounds **1**, **2**, **3**, and **4**.

In addition, the proton spectrum showed the resonances of one tertiary methyl group at ẟ_H_ 1.16 (3H, s, H-22), two methylenes at ẟ_H_ 1.28 (2H, m, H-23) and 2.05 (2H, m, H-7), and one methine at ẟ_H_ 1.87 (1H, m, H-6). The ^13^C NMR spectrum (Table 1) of **1** revealed 38 carbon signals, which were sorted by DEPT and HMBC experiments into eight methyls, eight methylenes, seven methines [including three aromatic carbons at ẟ_C_ 116.6 (C-12), 114.2 (C-15), and 123.9 (C-16)], and fifteen quaternary carbons. The carbon spectrum also displayed typical signals of a bicyclo[3.3.1]nonane ring system that included one ketone [ẟ_C_ 208.8 (C-9)], an enolized 1,3-diketone [ẟ_C_ 196.5 (C-1), 193.9 (C-10), 116.1 (C-2), and 191.9 (C-3)], three quaternary carbons [ẟ_C_ 70.3 (C-4), 51.8 (C-5), and 57.8 (C-8)], one methine at ẟ_C_ 39.7 (C-6), and a methylene at ẟ_C_ 43.3 (C-7) [37]. All these spectral data are close to those of guttiferone A, a polyisoprenylated benzophenone [38]. The discrepancy between guttiferone A and compound **1** was the isomerization of one isopent-2-enyl unit into an isopent-3-enyl unit. In addition, the Δ^1–2^ enol group in guttiferone A was present as Δ^2–(10)^ in compound **1**. The HMBC correlations (Figure 2) of H-12/C-10, C-14 (150.9), and C-16 and H-15/C-13 (144.8) and C-11 (132.0) indicated the presence of a catechol unit and supported the presence of the Δ^2–(10)^ enol group in the structure. The isopent-3-enyl unit was located at C-6 following the HMBC correlations of H-24, H-25, and H-22 with C-6 (39.7). Additional HBMC correlations of H-17/C-3 (ẟ_C_ 191.9) and C-9 (ẟ_C_ 208.8), H-34 (ẟ_H_ 1.91)/C-23 (ẟ_C_ 35.0) and C-5 (ẟ_C_ 51.8), and H-29 (ẟ_H_ 2.05)/C-8 (ẟ_C_ 57. 8) allowed the junction of isoprenyl groups at C-4, C-23, and C-8, respectively. The bicyclic ring system in **1** required that the isopentenyl groups on C-4 and C-8 be equatorial [38]. Furthermore, the lack of NOESY correlation between H-17 and H-22 suggested that the isopentenyl unit fixed at C-4 and the methyl group at C-5 were on the opposite sides. Nevertheless, the NOESY spectrum displayed an important correlation between CH_3_-22 (ẟ_H_ 1.16) and H-24 (ẟ_H_ 2.05), suggesting a relative *Cis*-configuration with the methyl group (Figure 2).

This information suggested that the relative stereochemistry of compound **1** could be identical to that of guttiferone A. This was further confirmed by the comparison of their optical rotation signs. Therefore, compound **1** was concluded to be a new polyprenylated acylphloroglucinol derivative named guttiferone U with the structure as shown.

Compounds **2** and **3** were obtained as an optically active mixture of a yellow amorphous solid with the same R_f_ on thin-layer chromatography (TLC) in different solvent systems. They were present as a 1:1 mixture based on their NMR peak intensities. The HR-ESIMS showed a sodium adduct peak at *m*/*z* 693.4141 [M + Na]^+^ (calcd for C_43_H_58_O_6_Na^+^, 693.4126) corresponding to the molecular formula, C_43_H_58_O_6_, a mass which was 68 a.m.u higher than that of compound **1**, suggesting the presence of an additional prenyl side chain (C_5_H_9_) when compared to **1**. The IR spectrum exhibited strong absorption bands at 3300 (hydroxy groups), 1729, and 1669 (for non-conjugated and conjugated carbonyl groups), and 1699 cm^−1^ (C=C). The UV spectrum displayed characteristic absorptions at *λ*_max_ 340 and 370 nm for the aromatic ring and conjugated carbonyl chromophores, respectively [38]. The analysis of the NMR spectra confirmed **2**/**3** to be polyprenylated benzophenone derivatives. The ^1^H NMR spectrum (Table 1) of the mixture exhibited signals of two ABX substitution patterns as pairs of duplicated signals at ẟ_H_ 7.39/7.38 (1H, d, *J* = 2.0 Hz, H-12), 7.14/7.07 (1H, dd, *J* = 8.2, 2.0 Hz, H-16), and 6.85/6.82 (1H, d, *J* = 8.2 Hz, H-15). The spectrum also displayed the characteristic signals of seven isopent-2-enyl groups {[ẟ_H_ 2.59/2.54 (2H, m, H-17), 4.95/4.95 (2H, m, H-18), 1.56/1.56 (6H, s, H-20), and 1.70/1.70 (6H, brs, H-21)], [ẟ_H_ 2.80/2.80 (2H, m, H-24), 5.02/5.02 (2H, m, H-25), 1.71/1.71 (6H, s, H-27), and 1.71/1.71 (6H, brs, H-28)], [ẟ_H_ 2.10/2.10 (2H, m, H-34), 5.22/5.22 (2H, m, H-35), 1.77/1.77 (6H, brs, H-37), and 1.63/1.63 (6H, brs, H-38)], [ẟ_H_ 1.94 (2H, m, H-39, **2**), 5.12 (1H, m, H-40, **2**), 1.67 (3H, brs, H-42, **2**), and 1.60 (3H, brs, H-43, **2**)]}, those of one isopent-3-enyl unit [ẟ_H_ 1.94 (2H, m, H-39, **3**), 1.21 (2H, m, H-40, **3**), 1.74 (3H, brs, H-42, **3**), and 4.79 (2H, brs, H-43, **3**)], and the signals of two isopentyl units [ẟ_H_ 3.00/3.05 (2H, m, H-29), 1.10/1.10 (2H, m, H-30), 0.99/0.99 (6H, s, H-32), and 1.27/1.29 (3H, s, H-33)]. The duplication of all these data in association with the mass data confirmed that **2/3** was a mixture of two polyisoprenylated benzophenone derivatives. The ^13^C NMR spectrum of **2/3** displayed characteristic signals for a bicyclo[3.3.1]nonane ring as pairs of carbons at ẟ_C_ 170.3/170.5 (C-1), 125.6/125.9 (C-2), 193.3/193.6 (C-3), 68.9/69.0 (C-4), 48.3/48.4 (C-5), 2×39.4 (C-6), 38.1/38.3 (C-7), 51.2/51.5 (C-8), and 206.2/206.3 (C-9) [37,39].

In addition, the combination of the DEPT 135 and HSQC confirmed the presence of seven isopent-2-enyl units, one isopent-3-enyl with the characteristic signals at δ_C_/δ_H_ [22.4/1.94 (C-39/H-39), 35.4/1.21 (C-40/H-40), 145.0 (C-41), 21.5/1.74 (C-42/H-42), and 110.4/4.79 (C-43/H-43)], and one dimethylpyrane moiety identified by the signals at δ_C_/δ_H_ {[28.3/0.89 (C-29/H-29), 41.8/1.10 (C-30/H-30), 86.2 (C-31), 27.9/0.89 (C-32/H-32), 20.6/1.27 (C-33/H-33), **2**], [28.3/0.94 (C-29/H-29), 42.9/1.10 (C-30/H-30, 86.5 (C-31), 28.0/0.89 (C-32/H-32), 20.7/1.29 (C-33/H-33), **3**]}. All these data indicated that the structures of **2/3** are closely related to shomburgkianone I [39]. The location of the dimethylpyrane moiety on the bicyclo unit was deduced by the HMBC correlation of H-29 (ẟ_H_ 3.00/3.05)/C-8 (ẟ_C_ 51.2/51.5), C-1 (δ_C_ 170.3/170.5), C-30 (δ_C_ 41.8/42.9), and C-31 (δ_C_ 86.5). All this evidence suggested that the only difference between compounds **2** and **3** was the isomerization of one isopent-2-enyl unit in **2** into an isopent-3-enyl unit in **3**; they were both located at C-23 by the HMBC correlation of H-39/C-23 (Figure 2). The locations of additional isoprenyl groups were evidenced by the HMBC correlations of H-17/C-3 and C-9, H-39/C-23, H-24/C-6 and C-7, and H-35/C-30. The relative configurations of the different stereogenic centers in **2** and **3** were deduced by the same manner to that of **1** [38]. Based on the above data, compounds **2** and **3** were found to be new polyisoprenylated benzophenone isomers named guttiferones V and W, respectively.

Compound **4** was isolated as a brown oil, ${\left[\alpha \right]}_{589\;}^{20}$: + 21.5 (*c* 0.5, MeOH). Its molecular formula, C_26_H_40_O_4_, with seven degrees of unsaturation was deduced from its HR-ESIMS, which showed the sodium adduct peak [M + Na]^+^ at *m/z* 439.2816 (calcd for C_26_H_40_O_4_Na^+^, 439.2819). The vibrational absorption bands at 3364, 1685, and 1620 cm^−1^ in the IR spectrum were consistent with those of the hydroxy, conjugated carbonyl, and olefinic groups, respectively. The UV spectrum showed an absorption band at λ_max_ 320 nm. The ^1^H NMR spectrum (Table 2) exhibited the signals of protons of two free hydroxy groups at δ_H_ [5.53 (1H, d, *J* = 6.5 Hz, OH-6) and 5.32 (1H, s, OH-7)]; three olefinic protons at δ_H_ [5.12 (1H, t, *J* = 7.1 Hz, H-2′) and 5.06 (2H, m, H-6′ and H-10′)]; one oxymethine at δ_H_ 3.90 (1H, d, *J* = 6.3 Hz H-6); eight methylene protons at δ_H_ [1.73 (1H, dd, *J* = 14.0, 6.2 Hz, H-3a)/1.22 (1H, m, H-3b), 2.03 (2H, m, H-4), 1.63 (1H, m, H-12a)/1.53 (1H, m, H-12b), and 2.03–2.05 (10H, m, H-1′, H-4′, H-5′, H-8′ and H-9′)]; six methyls {including four methyl linked to sp^2^ carbons at [δ_H_ 1.63 (3H, s, H-15′), 1.58 (3H, m, H-12′), and 1.55 (6H, s, H-13′/H-14′)]; and two sp^3^ carbons at [1.28 (3H, s, H-11) and 1.17 (3H, s, H-10)]}. Its ^13^C NMR (Table 2) spectrum exhibited the signals for 26 carbons, which were sorted by DEPT and HSQC into eight methylenes, four methines, and eight quaternary carbons, among which there was one *α*,*β*-conjugated carbonyl at δ_C_ 198.8 (C-5), and six methyl groups. The 6,7-dihydroxy-2,2,7-trimethyl-3,4,6,7-tetrahydrocyclopenta[*b*]pyran-5(2*H*)-one and farnesyl moieties were built based on the correlations observed in the ^1^H–^1^H COSY and HMBC spectra (Figure 2) [40,41]. The farnesyl unit was located at C-2 as proven by the HMBC correlations of H-3 and H-11/C-12 (δ_C_ 38.3) and C-2 (δ_C_ 82.2), and of H-1′ and H-12/C-2 (δ_C_ 82.2). All of the above evidence indicated that compound **4** is an unusual tocotrienol with a C5/C6 membered ring. The NOESY spectrum displayed a correlation (Figure 2) between H-6 (δ_H_ 3.90) and H-10 (δ_H_ 1.17), suggesting their *Cis*-orientation. Furthermore, the lack of a NOESY correlation between H-10 and H-11, which was biogenetically *α*-oriented in tocotrienol derivatives [41,42], allowed us to suggest a *β*-orientation for H-6 and H-10. Thus, compound **4** was characterized as 12-farnesyl-6,7-dihydroxy-7-methyl-3,4,6,7-tetrahydrocyclopenta[*b*]pyran-5(2*H*)-one, trivially named globuliferanol.

With regard to the chemophenetic contribution, fifteen compounds (**1–15**, Figure 1) sorted into five classes of secondary metabolites were isolated from the *n*-hexane soluble fraction of the stem bark of *S. globulifera*, including four polyprenylated benzophenones (**1**–**3**, **5**), among which there were three new derivatives (**1**–**3**), one new tocotrienol derivative (**4**), five xanthones (**6**–**10**), three steroids (**11**–**13**), one flavanol (**14**), and one triterpenoid (**15**). The presence of the polyprenylated benzophenones (**1**–**3**) is not surprising since benzophenones (polycyclic polyprenylated acylphloroglucinols) are known to be widespread in the Clusiaceae family [27]. Moreover, gaboxanthone (**6**), symphonin (**9**), lupeol (**15**), *β*-sitosterol (**11**), stigmasterol (**12**), and *β*-sitosterol 3-*O*-*β*-D-glucopyranoside (**13**) have been reported from *S. globulifera* [29,43,44]. In addition, the new tocotrienol derivative (**4**) has been isolated for the first time from the genus *Symphonia*. However, the literature indicates that tocotrienol derivatives have already been isolated from other genera of the Clusiaceae family, such as *Garcinia* [45,46] and *Clusia* [47]. To the best of our knowledge, guttiferone K (**5**), xanthone V2 (**7**), pyranojacareubin (**8**), and kaempferol (**14**) were isolated for the first time from the genus *Symphonia*. However, these compounds have been reported from plants of the Clusiaceae family. In fact, guttiferone K (**5**) has been previously isolated from the fruits of *Rheedia calcicole* [28], xanthone V2 (**7**) was also isolated from the root bark of *Vismia guineensis* [30], pyranojacareubin (**8**) was reported from the bark of *Calophyllum gracilipes* [31], and kaempferol (**14**) was obtained from the leaves of *V. guineensis* [48]. Furthermore, 1,5-dihydroxy-3-methoxyxanthone (**10**) was isolated for the first time from Clusiaceae.

### 2.2. Antileishmanial and Antibacterial Activities

Compounds (**1**–**10**) were assessed in vitro for their antileishmanial activity against *L. donovani* NR-48822 promastigotes and for their cytotoxicity toward Raw 264.7 macrophage cells (Table 3). Guttiferone K (**5**) exhibited the best antileishmanial activity against the parasite with an IC_50_ value of 3.30 ± 0.51 μg.mL^−1^ but with weak selectivity toward Raw 264.7 macrophage cells (SI = 1.57), while compounds **1**–**4**, **6**–**8**, and **10** showed moderate activity with IC_50_ values ranging from 10.80 to 15.98 μg.mL^−1^. The assessed compounds were more active than the *n*-hexane soluble fraction from which they were obtained. The inactivity of the MeOH crude extract and EtOAc fraction may be due to the antagonistic effect of its constituents. The majority of the active compounds were xanthones or benzophenones, which are known to possess antileishmanial activity.

The methanol crude extract, the *n*-hexane, EtOAc, and *n*-butanol soluble fractions along with some the isolates were assessed for their antibacterial activity against seven bacterial strains: *Salmonella typhi CPC, S. enterica NR13555, Staphylococcus aureus ATCC43300*, *S. aureus ATCC25923*, *Klebsiella pneumoniae clinical isolate, K. pneumoniae NR41388*, and *Pseudomonas aeruginosa HM801* (Table 4).

The MeOH crude extract and the *n*-BuOH fraction exhibited moderate activity, while the *n*-hexane and EtOAc fractions exhibited good antibacterial activities on at least two strains with MIC values ranging from 15.7 to 31.2 μg.mL^−1^, except on *S. aureus* ATCC43300, which was not susceptible to the EtOAc fraction. Compounds **1** and **2**–**5** displayed good to moderate activity against these strains, with MIC values ranging from 3.9 to 62.5 μg.mL^−1^, except for *S. aureus* ATCC43300, which was not susceptible.

These results highlight the knowledge on the potential of guttiferone derivatives as potent antileishmanial and antibacterial agents [15,49], and thus justify the use of this plant in traditional medicine to treat skin and bacterial diseases [19,23].

## 3. Materials and Methods

### 3.1. General Experimental Procedures

Column chromatography (CC) separations were carried out with silica gel (230–400, 70–230 mesh Merck, Darmstadt, Germany) and Sephadex LH-20 (Sigma-Aldrich, Munich, Germany). Pre-coated aluminum-backed silica gel 60 F254 sheets were used for thin-layer chromatography. Spots were visualized under UV light (254 nm and 366 nm) or using a diluted solution of sulfuric acid followed by heating at about 80 °C. Optical rotation was determined by using a JASCO DIP-3600 polarimeter (JASCO, Tokyo, Japan). UV spectra were recorded on a UV-3100 PC spectrophotometer. The ^1^H and ^13^C NMR spectra were recorded at 500 MHz or 600 MHz and 125 MHz or 150 MHz, respectively. The spectrometers were Bruker AM Avance DRX 500 (Rheinstetten, Germany; ^1^H NMR, 500 MHz and ^13^C NMR, 125 MHz) and Bruker Avance 600 (^1^H NMR, 600 MHz and ^13^C NMR, 150 MHz). Infrared (IR) spectra (KBr tablet or film) were recorded on a Bruker Tensor 27 FTIR-spectrometer equipped with a diamond ATR. High-resolution mass spectra were recorded on a Bruker QTOF compact spectrometer equipped with an ESI source.

### 3.2. Plant Material and Identification

The stem bark of *S. globulifera* was harvested in May 2016 in Bangangte (west region of Cameroon) and identified by Mr. Nana Victor, a retired botanist at the National Herbarium of Cameroon, where a voucher specimen (29529 SRFK) was already available.

### 3.3. Extraction and Isolation

The stem bark of *S. globulifera* was chopped, air-dried, and then ground to give 10.3 kg of powder, which was extracted by maceration using methanol for 48 h, three times each. The extract was freed from the solvent using a rotavapor to yield 638.7 g of MeOH extract. This extract was subjected to bioguided fractionation toward the *L. donovani* NR-48822 promastigote strain. Part of the extract (628.2 g) was dissolved in water and successively partitioned with *n*-hexane, EtOAc, and *n*-BuOH. After evaporation of each solvent under reduced pressure, 146.8 g of *n*-hexane, 76.2 g of EtOAc, and 42.3 g of *n*-BuOH fractions were obtained. A part of the soluble *n*-hexane fraction of *S. globulifera* (140.1 g), which was the most active fraction, was separated over a silica gel CC using a mixture of *n*-hexane-EtOAc solvent systems of increasing polarities. Ninety-eight (98) subfractions were obtained and combined based on their TLC profiles into five fractions labeled F1 (34.8 g; *n*-hexane/EtOAc, 19:1–4:1, *v*/*v*), F2 (30.2 g; *n*-hexane/EtOAc, 9:1–3:1, *v*/*v*), F3 (25.2 g; *n*-hexane/EtOAc, 4:1–3:2, *v*/*v*), F4 (15.7 g; *n*-hexane/EtOAc, 3:2–1:1, *v*/*v*), and F5 (15.6 g; *n*-hexane/EtOAc, 1:1–0:1, *v*/*v*). The CC of fraction F1 (34.8 g) over silica gel using mixtures of *n*-hexane/EtOAc (19:1–4:1, *v*/*v*) gave compound **15** (5.5 mg) and a mixture of (1:1) **11** and **12** (5.8 mg). The CC of fraction F2 (30.2 g) over Sephadex LH-20 eluting with MeOH followed by MPLC using normal phase pre-packed silica-gel columns as the stationary phase with the *n*-hexane/EtOAc (9:1–3:2, *v*/*v*) gradient solvent system and a second purification by repeated Sephadex LH-20 CC yielded the mixture of (1:1) **2** and **3** (10.5 mg), **6** (5.2 mg), **1** (15.0 mg), **7** (5.6 mg), **8** (6.3 mg), **9** (5.3 mg), and **14** (5.1 mg). In the same way, F3 (25.2 g) was subjected to Sephadex LH-20 CC eluting with MeOH followed by MPLC using normal phase pre-packed silica-gel columns as the stationary phase with the mixture of *n*-hexane/EtOAc (9:1–3:2, *v*/*v*) gradient solvent systems and purified a second time by repeated Sephadex LH-20 CC to afford compounds **5** (20.5 mg) and **10** (5.0 mg). The CC of fraction F4 (15.7 g) over Sephadex LH-20 eluting with MeOH followed by MPLC using normal phase pre-packed silica-gel columns as the stationary phase with *n*-hexane/EtOAc (7:3–1:1, *v*/*v*) gradient solvent system afforded compound **4** (10.4 mg). The CC of fraction F5 (15.6 g) over silica gel using mixtures of *n*-hexane/EtOAc (1:1–0:1, *v*/*v*) gave compound **13** (7.4 mg).

#### 3.3.1. Guttiferone U (**1**)

Yellow amorphous solid, ${\left[\alpha \right]}_{589}^{20}$: + 93 (*c* 0.5, MeOH). IR(KBr) ν_max_ 3735, 2974, 2924, 2362, 2337, 1725, 1646, 1289, 1020, 987, 828, 669 cm^−1^; for ^1^H and ^13^C NMR data (acetone-*d*_6_, 600 and 150 MHz), see Table 1; HRESIMS *m/z* 625.3494 [M + Na]^+^ (calcd for C_38_H_50_O_6_Na^+^, 625.3500).

#### 3.3.2. Guttiferone V (**2**)/Guttiferone W (**3**)

Yellow amorphous solid, IR(KBr) ν_max_ 3735, 2967, 2929, 2854, 2360, 2341, 1729, 1669, 1594, 1540, 1521, 1289, 1119, 985, 952, 821, 668 cm^−1^; for ^1^H and ^13^C NMR data (acetone-*d*_6_, 600 and 150 MHz), see Table 1; HRESIMS *m/z* 693.4141 [M + Na]^+^ (calcd for C_43_H_58_O_6_Na^+^, 693.4126).

#### 3.3.3. Globuliferanol (**4**)

Brown oil, ${\left[\alpha \right]}_{589}^{20}$: + 21.5 (*c* 0.5, MeOH). IR(KBr) ν_max_ 3364, 2964, 2923, 2360, 2341, 1685, 1620, 1409, 1375, 1292, 1084, 1029, 806 cm^−1^; for ^1^H and ^13^C NMR data (DMSO-*d*_6_, 600 and 150 MHz), see Table 2; HRESIMS *m/z* 439.2816 [M + Na]^+^ (calcd for C_26_H_40_O_4_Na^+^, 439.2819).

### 3.4. Antileishmanial and Cytotoxicity Assays

The antileishmanial activity on cultured *L. donovani* 1S (MHOM/SD/62/1S) promastigotes was evaluated using the resazurin colorimetric method as described by Siqueira-Neto et al. (2010) [50] with little modifications. They were all assessed in triplicate at concentrations ranging from 100 to 0.16 µg/mL for the extract and 50–0.08 µg/mL for the compounds. Test plates were incubated for 28 h at 28 °C, followed by the addition of 1 mg/mL resazurin. The negative and positive controls were 0.1% DMSO and amphotericin B (10–0.016 µg/mL), respectively. The cytotoxicity profile of the crude extract and compounds was assessed using the Alamar blue assay (Mosman, 1983) against Raw 264.7 macrophage cells.

### 3.5. Antibacterial Bioassay

The minimum inhibitory concentration (MIC) of the samples was evaluated following the broth microdilution method as described by Eloff, with light modifications [51]. The extracts, compounds, and reference drug were dissolved in DMSO-MHB. The strain inocula were prepared and their turbidity was adjusted to 0.5 McFarland standard to give an approximate 1.5 × 10^8^ CFU/mL. Gentamicin was used as the positive control. Briefly, one hundred microliters of Mueller Hinton Broth was added to all wells of the 96-well plate, and 100 µL of the compounds/extracts was introduced into the wells in the first row (A) and mixed thoroughly. The sample mixture (100 µL) was removed from the well from row A to perform a twofold serial dilution down the rows (B–H). The last 100 µL was discarded. Then, 100 µL of the inoculum was introduced into the corresponding wells. The final volume in each well was 200 µL. Each extract concentration was assayed in triplicate and each test was performed twice. After an incubation period of 18 h at 37 °C, 20 µL of Alamar Blue was added to each well. The plates were then re-incubated for 30 min at 37 °C. A blue color in the well was scored as ‘‘no bacterial growth”, while a pink color was scored as a ‘‘growth occurrence”. MIC values were read at those concentrations where a pronounced change in color formation was noticed (from blue to pink).

## 4. Conclusions

In addition to enriching the knowledge on the chemistry of *S. globulifera*, this work represents a significant chemophenetic contribution to this species. It has provided further information with regard to possible chemophenetic markers of *S. globulifera* and showed the presence of uncommon metabolites encountered in this species. Moreover, the results obtained for the biological evaluation of isolated compounds support the use of *S. globulifera* in folk medicine.

## Figures and Tables

**Table 1:** ^1^H (600 MHz) and ^13^C (150 MHz) NMR data of compounds **1**–**3** in acetone-*d*_6_ (*δ* in ppm).

Position	(1)		(2)		(3)	
	*δ*_C_	*δ*_H_ (m, *J* (Hz))	*δ*_C_	*δ*_H_ (m, *J* (Hz))	*δ*_C_	*δ*_H_ (m, *J* (Hz))
1	196.5		170.3		170.5	
2	116.1		125.6		125.9	
3	191.9		193.4		193.6	
4	70.3		68.9		69.0	
5	51.8		48.3		48.4	
6	39.7	1.87 (m)	39.4	1.88 (m)	39.4	1.86 (m)
7	43.3	2.05 (m)	38.1	2.31 (m)	38.3	2.28 (m)
8	57.8		51.2		51.5	
9	208.8		206.2		206.3	
10	193.9		191.2		191.3	
11	132.0		130.3		130.4	
12	116.6	7.23 (d, 2.2)	114.7	7.39 (d, 2.0)	114.9	7.38 (d, 2.0)
13	144.8		144.8		144.9	
14	150.9		150.2		150.3	
15	114.2	6.81 (d, 8.3)	114.4	6.85 (d, 8.2)	114.6	6.82 (d, 8.2)
16	123.9	7.06 (dd, 8.3, 2.1)	122.7	7.14 (dd, 8.2, 2.0)	123.0	7.07 (dd, 8.2, 2.0)
17	25.2	2.68 (m)	24.9	2.59 (m)	24.9	2.54 (m)
18	120.7	5.15 (brs)	120.5	4.95 (m)	120.6	4.95 (m)
19	134.2		133.1		133.3	
20	25.2	1.71 (s)	25.4	1.56 (brs)	25.5	1.56 (brs)
21	17.5	1.65 (s)	17.3	1.70 (brs)	17.4	1.70 (brs)
22	18.1	1.16 (s)	17.9	1.18 (brs)	17.9	1.18 (brs)
23	35.0	1.28 (m)/1.31 (m)	35.1	1.21 (m)/1.43 (m)	35.3	1.21 (m)/1.43 (m)
24	28.4	2.05 (m)	28.8	2.80 (m)	28.9	2.80 (m)
25	35.4	1.91 (m)	125.5	5.02 (m)	125.5	5.02 (m)
26	145.6		132.2		132.2	
27	21.9	1.70 (brs)	25.2	1.71 (brs)	25.2	1.71 (brs)
28	109.4	4.66 (brs)	17.6	1.71 (brs)	17.6	1.71 (brs)
29	28.4	2.05 (m)	28.3	3.00 (d, 3.3)/ 3.05 (dd, 14.0, 3.3)	28.3	3.00 (d, 3.3)/ 3.05 (dd, 14.0, 3.3)
30	123.9	5.06 (m)	41.8	1.10 (m)	42.9	1.10 (m)
31	134.2		86.2		86.5	
32	25.1	1.65 (brs)	27.9	0.89 (brs)	28.0	0.89 (brs)
33	17.2	1.53 (brs)	20.6	1.27 (brs)	20.7	1.29 (brs)
34	22.7	1.91 (m)	29.2	2.10 (brs)	29.3	2.10 (brs)
35	124.3	5.11 (m)	122.0	5.22 (m)	122.0	5.22 (m)
36	131.2		132.9		132.9	
37	25.0	1.68 (s)	25.0	1.77 (brs)	25.1	1.77 (brs)
38	16.9	1.60 (brs)	17.11	1.63 (brs)	17.2	1.63 (brs)
39			22.4	1.94 (m)	22.4	1.94 (m)
40			124.2	5.12 (m)	35.4	1.21 (m)
41			131.2		145.0	
42			24.9	1.67 (brs)	21.5	1.74 (brs)
43			16.7	1.60 (brs)	110.4	4.79 (brs)

**Table 2:** ^1^H (600 MHz) and ^13^C (150 MHz) NMR data of compound **4** in DMSO-*d*_6_ (*δ* in ppm).

Position	^13^C	^1^H
	*δ*_C_	*δ*_H_ (m, *J* (in Hz))
2	82.2	
3	29.9	1.73 (dd, 14.0, 6.2)/1.22 (m)
4	13.7	2.03 (m)
5	198.8	
6	80.9	3.90 (d, 6.3)
7	75.9	
8	181.6	
9	109.5	
10	21.4	1.17 (s)
11	24.1	1.28 (s)
12	38.3	1.63 (m)/1.53 (brs)
1′	21.9	2.03 (m)
2′	124.2	5.12 (t, 7.1)
3′	135.2	
4′	39.5	2.03 (m)
5′	26.6	2.03 (m)
6′	124.3	5.06 (m)
7′	134.8	
8′	39.6	2.03 (m)/1.93 (q, 6.3)
9′	26.4	2.04 (m, 2H)
10′	124.5	5.06 (m)
11′	131.0	
12′	16.22	1.58 (m)
13′	16.25	1.55 (s)
14′	18.0	1.55 (s)
15′	25.9	1.63 (s)
OH-6	-	5.53 (d, 6.5)
OH-7	-	5.32 (s)

**Table 3:** Antileishmanial and cytotoxic activities of extract, fractions, and compounds from the stem bark of *S. globulifera*.

Extracts/ Compounds	Antileishmanial Activity IC_50_ ± SD (μg/mL)	Macrophages CC_50_ ± SD (μg/mL)	Selectivity Index SI ± SD (=CC5_0_/IC_50_)
ME	>100		
HF	43.11 ± 0.01	>20	
EF	>100		
BF	>100		
**1**	12.91 ± 1.11	28.06 ± 5.72	2.17
**2** and **3**	12.13 ± 1.08	9.60 ± 0.26	0.79
**4**	14.03 ± 1.14	>20	>1.39
**5**	3.30 ± 0.51	5.20 ± 0.02	1.57
**6**	15.97 ± 1.20	>20	>1.25
**7**	12.91 ± 1.11	>20	>1.54
**8**	12.91 ± 1.11	>20	>1.54
**9**	ND	ND	
**10**	47.04 ± 1.67	>20	>0.42
Amphotericin B	0.048		

ND: not determined; BF: *n*-butanol fraction; HF: *n*-hexane fraction; ME: methanol extract; EF: ethyl acetate fraction.

**Table 4:** Antibacterial activity of extract, fractions, and compounds (MIC in μg.mL^−1^).

Extracts/ Compounds	Antibacterial Activity (MIC in µg.mL^−1^)						
	St	Se	Sa	Sau	Kpc	Kp	Pa
ME	250	-	250	250	250	1000	125
HF	15.7	62.5	250	62.5	31.2	125	31.2
EF	62.5	500	1000	31.2	31.2	500	31.2
BF	500	-	-	500	1000	1000	500
**1**	3.9	62.5	125	3.9	3.9	62.5	3.9
**2** and **3**	15.6	62.5	500	62.5	15.6	62.5	15.6
**4**	31.2	125	500	62.5	31.2	62.5	31.2
**5**	3.9	125	250	31.2	3.9	62.5	3.9
Gentamycin	0.048	0.07	0.07	0.03	0.048	0.07	0.048

St: *Salmonella typhi* CPC; Se: *S. enterica* NR13555; Sa: *Staphylococcus aureus* ATCC43300; Sau: *S. aureus* ATCC25923; Kpc: *Klebsiella pneumoniae* clinical isolate; Kp: *K. pneumoniae* NR41388; Pa: *Pseudomonas aeruginosa* HM801; -: >1000 μg/mL; BF: *n*-butanol fraction; HF: *n*-hexane fraction; ME: methylene chloride/methanol extract; EF: ethyl acetate fraction.

## Data Availability

See Supplementary Materials.

## Supplementary Materials

The following supporting information can be downloaded at: https://www.mdpi.com/article/10.3390/molecules28062473/s1, Figure S1: HRESIMS of compound **1**; Figure S2: UV spectrum of compound **1**; Figure S3: IR spectrum of compound **1**; Figure S4: ^1^H NMR (Acetone-*d*_6_, 600 MHz) spectrum of compound **1**; Figure S5: ^1^H–^1^H COSY spectrum of compound **1**; Figure S6: ^13^C NMR (Acetone-*d*_6_, 150 MHz) spectrum of compound 1; Figure S7: DEPT 135 NMR spectrum of compound **1**; Figure S8: HSQC spectrum of compound **1**; Figure S9: HMBC spectrum of compound **1**; Figure S10: NOESY spectrum of compound **1**; Figure S11: HRESIMS of compound **2/3**; Figure S12: UV spectrum of compound **2/3**; Figure S13: IR spectrum of compound **2/3**; Figure S14: ^1^H NMR (Acetone-*d*_6_, 600 MHz) spectrum of compound **2/3**; Figure S15: ^1^H–^1^H COSY spectrum of compound **2/3**; Figure S16: ^13^C NMR (Acetone-*d*_6_, 150 MHz) spectrum of compound **2/3**; Figure S17: DEPT 135 spectrum of compound **2/3**; Figure S18: HSQC spectrum of compound **2/3**; Figure S19: HMBC spectrum of compound **2/3**; Figure S20: NOESY spectrum of compound **2/3**; Figure S21: HRESIMS of compound **4**; Figure S22: UV spectrum of compound **4**; Figure S23: IR spectrum of compound **4**; Figure S24: ^1^H NMR (DMSO-*d*_6_, 600 MHz) spectrum of compound **4**; Figure S25: ^1^H–^1^H COSY spectrum of compound **4**; Figure S26: ^13^C NMR (DMSO-*d*_6_, 150 MHz) spectrum of compound **4**; Figure S27: DEPT 135 spectrum of compound **4**; Figure S28: HSQC spectrum of compound **4**; Figure S29: HMBC spectrum of compound **4**; Figure S30: NOESY spectrum of compound **4**; Figure S31: ^1^H NMR (DMSO-*d*_6_, 600 MHz) spectrum of compound **5**; Figure S32: ^13^C NMR (DMSO-*d*_6_, 150 MHz) spectrum of compound **5**; Figure S33: ^1^H NMR (DMSO-*d*_6_, 600 MHz) spectrum of compound **6**; Figure S34: ^13^C NMR (DMSO-*d*_6_, 150 MHz) spectrum of compound **6**; Figure S35: ^1^H NMR (Acetone, 500 MHz) spectrum of compound **7**; Figure S36: ^13^C NMR (Acetone, 125 MHz) spectrum of compound **7**; Figure S37: ^1^H NMR (DMSO-*d*_6_, 500 MHz) spectrum of compound **8**; Figure S38: ^13^C NMR (DMSO-*d*_6_, 125 MHz) spectrum of compound **8**; Figure S39: ^1^H NMR (DMSO-*d*_6_, 500 MHz) spectrum of compound **9**; Figure S40: ^13^C NMR (DMSO-*d*_6_, 125 MHz) spectrum of compound **9**; Figure S41: ^1^H NMR (Acetone, 500 MHz) spectrum of compound **10**; Figure S42: ^13^C NMR (Acetone, 125 MHz) spectrum of compound **10**; Figure S43: ^1^H NMR (CDCl_3_, 600 MHz) spectrum of compound **11+12**; Figure S44: ^13^C NMR (CDCl_3_, 150 MHz) spectrum of compound **11+12**; Figure S45: ^1^H NMR (Pyridine-*d_5_*, 600 MHz) spectrum of compound **13**; Figure S46: ^13^C NMR (Pyridine-*d_5_*, 150 MHz) spectrum of compound **13**; Figure S47: ^1^H NMR (Acetone, 600 MHz) spectrum of compound **14**; Figure S48: ^13^C NMR (Acetone, 150 MHz) spectrum of compound **14**; Figure S49: ^1^H NMR (CDCl_3_, 600 MHz) spectrum of compound **15**; Figure S50: ^13^C NMR (CDCl_3_, 150 MHz) spectrum of compound **15**.

## Sample Availability

Samples of the compounds are not available from the authors.

## References

[1] Burza S., Croft S.L., Boelaert M. (2018). Leishmaniasis. Lancet.

[2] Ngouateu O.M., Dondji B. (2022). Leishmaniasis in Cameroon and neighboring countries: An overview of current status and control challenges. Curr. Res. Parasitol. Vector-Borne Dis..

[3] Uliana S.R., Trinconi C.T., Coelho A.C. (2018). Chemotherapy of leishmaniasis: Present challenges. Parasitology.

[4] WHO (2020). Global leishmaniasis surveillance, 2017–2018, and first report on five additional indicators. Wkly. Epidemiol. Rec..

[5] Sasidharan S., Saudagar P. (2021). Leishmaniasis: Where we are and where we are heading?. Parasitol. Res..

[6] Yimer M., Nibret E., Yismaw G. (2022). Updates on prevalence and trends status of visceral leishmaniasis at two health facilities in amhara regional state, northwest Ethiopia: A retrospective study. Biochem. Res. Int.

[7] Kaye P.M., Mohan S., Mantel C., Malhame M., Revill P., Rutte E.L., Parkash V., Layton A.M., Lacey C.J.N., Malvoti S. (2021). Overcoming roadblocks in the development of vaccines for leishmaniasis. Exp. Rev. Vacc..

[8] Kitano H., Sanjoba C., Goto Y., Iwamoto Y., Kitagawa K., Nomura T., Shigemoto N., Hide M., Matsumoto Y., Ohge H. (2021). Complicated cutaneous leishmaniasis caused by an imported case of *Leishmania tropica* in Japan: A case report. Trop. Med. Health.

[9] Mann S., Frasca K., Scherre S., Henao-Martinez A.F., Newman S., Ramanan P., Suarez J.A. (2021). A review of leishmaniasis: Current knowledge and future directions. Curr. Trop. Med. Rep..

[10] Monzote L., Gonzalez D., Blanco O., Fraga J., Capo V., Herrera A., Montalvo A.M. (2022). Imported cases of cutaneous leishmaniasis in Cuba, 2017: Role of human movement. Trop. Dis. Travel Med. Vaccines..

[11] WHO (2021). Ending the Neglected to Attain the Sustainable Development Goals: A Road Map for Neglected Tropical Diseases 2021−2030.

[12] Freitas-Junior L.H., Chatelain E., Kim H.A., Siqueira-Neto J.L. (2012). Visceral leishmaniasis treatment: What do we have, what do we need and how to deliver it?. Int. J. Parasitol. Drugs Drug. Resist..

[13] Fotie J., Bohle D.S., Olivier M., Gomez M.A., Nzimiro S. (2007). Trypanosomial and antileishmanial dihydrochelerythrine derivatives from *Garcinia lucida*. J. Nat. Prod..

[14] Lenta B.N., Vonthron-Sénécheau C., Weniger B., Devkota K.P., Ngoupayo J., Kaiser M., Sewald N. (2007). Leishmanicidal and cholinesterase inhibiting activities of phenolic compounds from *Allanblackia monticola* and *Symphonia globulifera*. Molecules.

[15] Azebaze A.G.B., Ouahouo B.M.W., Vardamides J.C., Valentin A., Kuete V., Acebey L., Meyer M. (2008). Antimicrobial and antileishmanial xanthones from the stem bark of *Allanblackia gabonensis* (Guttiferae). Nat. Prod. Res..

[16] Garba J.K., Nguengang R.T., Youmbi G.T., Menache J.N., Ngansop C.A.N., Bankeu J.J.K., Chouna J.R., Boyom F.F., Sewald N., Lenta B.N. (2022). Antileishmanial, antibacterial and cytotoxicity activity of the extracts, fractions, and compounds from the fruits and stem bark of *Pentadesma butyraceae* Sabine. Z. Naturforsch. B.

[17] Dick C.W., Heuertz M. (2008). The complex biogeographic history of widespread tropical tree species. Evolution.

[18] Marti G., Eparvier V., Moretti C., Prado S., Grellier P., Hue N., Thoison O., Delpech B., Gueritte F., Litaudon M. (2010). Antiplasmodial benzophenone derivatives from the root barks of *Symphonia globulifera* (Clusiaceae). Phytochemistry.

[19] Ssegawa P., Kasenene J.M. (2007). Medicinal plant diversity and uses in the Sango bay area, southern Uganda. J. Ethnopharmacol..

[20] Fromentin Y., Cottet K., Kritsanida M., Mihel S., Gaboriaud-Kolar N., Lallemand M.C. (2014). *Symphonia globulifera*, a widespread source of complex metabolites with potent biological activities. Planta Med..

[21] Majekodunmi S.O., Aliga U.L. (2017). A systematic study on flow ability and compressibility of *Symphonia globulifera* stem bark powder for tablet dosage form. Am. J. Biomed. Eng..

[22] Irvine F.R. (1961). Woody Plants of Ghana with Special Reference to Their Uses.

[23] Gupta M.P., Solis P.N., Calderon A.I., Guinneau-Sinclair F., Correa M., Galdames C., Ocampo R. (2005). Medical ethnobotany of the tribes of bocas del toro Panama. J. Ethnopharmacol..

[24] Lopez A., Hudson J.B., Towers G.H.N. (2001). Antiviral and antimicrobial activities of Colombian medicinal plants. J. Ethnopharmacol..

[25] Nkengfack A.E., Mkounga P., Meyer M., Fomum Z.T., Bodo B. (2002). Globulixanthones C, D and E: Three prenylated xanthones with antimicrobial properties from the root bark of *Symphonia globulifera*. Phytochemistry.

[26] Mkounga P., Fomum Z.T., Meyer M., Bodo B., Nkemgfack A.E. (2009). Globulixanthone F, a new polyoxygenated xanthone with an isoprenoid group and two antimicrobial biflavonoids from the stem bark of *Symphonia globulifera*. Nat. Prod. Commun..

[27] Fromentin Y., Gaboriaud-Kolar N., Lenta B.N., Wansi J.D., Buisson D., Mouray E., Michel S. (2013). Synthesis of novel guttiferone A derivative: In-vitro evaluation toward *Plasmodium falciparum*, *Trypanosoma brucei* and *Leishmania donovani*. Eur. J. Med. Chem..

[28] Cao S., Brodie P.J., Miller J.S., Ratovoson F., Birkinshaw C., Randrianasolo S., Kingston D.G. (2007). Guttiferones. K and L, antiproliferative compounds of *Rheedia calcicola* from the Madagascar rainforest. J. Nat. Prod..

[29] Ngouela S., Lenta B.N., Noungoue D.T., Ngoupayo J., Boyom F.F., Tsamo E., Connolly J.D. (2006). Antiplasmodial and antioxidant activities of constituents of the seed shells of *Symphonia globulifera* Linn f. Phytochemistry.

[30] Botta B., Monachè D.F., Monache D.G., Kabangu K. (1986). Acetylvismione D from *Psorospermum febrifugum*. Phytochemistry.

[31] Cao S.G., Lim T.B., Sim K.Y., Goh S.H. (1997). A highly prenylated xanthone from the bark of *Calophyllum gracilipes* (Guttiferae). Nat. Prod. Lett..

[32] Lenta B.N., Ngouela S., Noungoue D.T., Tsamo E., Connolly J.D. (2004). Symphonin: A new prenylated pyranoxanthone with antimicrobial activity from the seeds of *Symphonia globulifera* (Guttiferae). Bull. Chem. Soc. Ethiop..

[33] Tosa H., Iinuma M., Murakami K.I., Ito T., Tanaka T., Chelladurai V., Riswan S. (1997). Three xanthones from *Poeciloneuron pauciflorum* and *Mammea acuminate*. Phytochemistry.

[34] Ahmed Y., Rahman S., Akhtar P., Islam F., Rahman M., Yaakob Z. (2013). Isolation of steroids from *n*-hexane extract of the leaves of *Saurauia roxburghii*. Int. Food. Res. J..

[35] Xiao Z.P., Wu H.K., Wu T., Shi H., Hang B., Aisa H.A. (2006). Kaempferol and quercetin flavonoids from *Rosa rugose*. Chem. Nat. Comp..

[36] Silva A.T.M., Magalhaes C.G., Duarte L.P., Mussel W.N., Ruiz A.L.T.G., Shiozawa L., Carvalho J.E., Trindade C.T., Filho S.A.V. (2017). Lupeol and its esters: NMR, powder XRD data and *in vitro* evaluation of cancer cell growth. Braz. J. Pharm. Sci..

[37] Bailly C., Vergoten G. (2021). Anticancer properties and mechanism of action of oblongifolin C, guttiferone K and related poplyprenylated acylphloroglucinols. Nat. Prod. Bioprospect..

[38] Gustafson K.R., Blunt J.W., Munro M.H., Fuller R.W., McKee T.C., Cardellina II J.H., Boyd M.R. (1992). The guttiferones, HIV-inhibitory benzophenones from *Symphonia globulifera*, *Garcinia livingstonei*, *Garcinia ovalifolia* and *Clusia rosea*. Tetrahedron.

[39] Nguyen H.T., Nguyen T.T., Duong T.H., Tran N.M.A., Nguyen C.H., Nguyen T.H.A., Sichaem J. (2022). *α*-Glucosidase inhibitory and antimicrobial benzoylphloroglucinols from *Garcinia schomburgakiana* fruits: *In vitro* and *in silico* studies. Molecules.

[40] Ohnmacht S., West R., Simionescu R., Atkinson J. (2008). Assignment of the ^1^H and ^13^C NMR of tocotrienols. Magn. Reson. Chem..

[41] Zeutsop J.F., Zebaze N.J., Nono R., Frese M., Chouna J.R., Lenta N.B., Nkeng-Efouet-Alango P., Sewald N. (2022). Antioxydant and cytotoxicity activities of *δ*-tocotrienol from the seed of *Allophylus africanus*. Nat. Prod. Res..

[42] Collakova E., DellaPenna D. (2001). Isolation and functional analysis of homogentisate phytyltransferase from *Synechocystis sp*. PCC 6803 and *Arabidopsis*. Plant Physiol..

[43] Suffredini I.B., Paciencia M.L.B., Díaz I.E., Frana S.A., Bernardi M.M. (2017). Mice behavioral phenotype changes after administration of Anani (*Symphonia globulifera*, Clusiaceae), an alternative Latin American and African medicine. Pharmacogn. Mag..

[44] Téné D.G., Tih A.E., Kamdem M.H.K., Talla R.M., Diboue P.H.B., Melongo Y.K.D., Ghogomu R.T. (2021). Antibacterial and antioxidant activities of compounds isolated from the leaves of *Symphonia globulifera* (Clusiaceae) and their chemophenetic significance. Biochem. Syst. Ecol..

[45] Tan X., Han X., Teng H., Li Q., Chen Y., Lei X., Yang G. (2021). Structural elucidation of garcipaucinones A and B from *Garcinia paucinervis* using quantum chemical calculations. J. Nat. Prod..

[46] Fuentes R.G., Pearce K.C., Du Y., Rakotondrafara A., Valenciano A.L., Cassera M.B., Kingston D.G. (2018). Phloroglucinols from the roots of *Garcinia dauphinensis* and their antiproliferative and antiplasmodial activities. J. Nat. Prod..

[47] Marques E.D.J., Ferraz C.G., dos Santos I.B., dos Santos I.I., El-Bachá R.S., Ribeiro P.R., Cruz F.G. (2021). Chemical constituents isolated from *Clusia criuva* subsp. *Criuva* and their chemophenetics significance. Biochem. Sys. Ecol..

[48] Mbaveng A.T., Kuete V., Nguemeving J.R., Penlap B.V., Nkengfack A.E., Meyer J.M., Krohn K. (2008). Antimicrobial activity of the extracts and compounds obtained from *Vismia guineensis* (Guttiferae). Asian J. Tradit. Med..

[49] Iinuma M., Tosa H., Tanaka T., Kanamaru S., Asai F., Kobayashi Y., Shimano R. (1996). Antibacterial activity of some *Garcinia* benzophenone derivatives against methicillin-resistant *Staphylococcus aureus*. Biol. Pharm. Bull..

[50] Siqueira-Neto J.L., Song O.R., Oh H., Sohn J.H., Yang G., Nam J., Jang J., Cechetto J., Lee C.B., Moon S. (2010). Antileishmanial high-throughput drug screening reveals drug candidates with new Scaffolds. PLoS Negl. Trop. Dis..

[51] Eloff J.N. (1998). A sensitive and quick microplate method to determine the minimal inhibitory concentration of plant extracts for bacteria. Planta Med..

