# Supplementary material for: Constituents of the Stem Bark of Symphonia globulifera Linn. f. with Antileishmanial and Antibacterial Activities
Source: Molecules. 2023 Mar 8;28(6):2473. doi: 10.3390/molecules28062473 (PMC10053230; doi:10.3390/molecules28062473)
Supplement: Supplementary file 1 [file molecules-28-02473-s001.zip › molecules-2253766-supplementary.pdf]

Supporting material

Article

Constituents of the stem bark of *Symphonia globulifera* Linn. f. with antileishmanial and antibacterial activities

Ruland Tchuinkeu Nguengang [1,2], Billy Toussie Tchegnitegni [3], Eric Carly Nono Nono [1], Georges Bellier Tabekoueng [4], Yannick Stéphane Fotsing Fongang [5], Jean Jules Kezetas Bankeu [6], Jean Rodolphe Chouna [3], Céline Nguefeu Nkenfou [7], Fabrice Boyom Fekam [8], Norbert Sewald [9,*], Bruno Ndjakou

Table of contents

Figure S1: HRESIMS of compound 1. 3

Figure S2: UV Spectrum of compound 1 3

Figure S3: IR Spectrum of compound 1 4

Figure S4: ^{1}H NMR (Acetone-d_6, 600 MHz) spectrum of compound 1 4

Figure S5: ^{1}H–^{1}H COSY spectrum of compound 1 5

Figure S6: ^{13}C NMR (Acetone-d_6, 150 MHz) spectrum of compound 1 5

Figure S7: DEPT 135 NMR spectrum of compound 1 6

Figure S8: HSQC spectrum of compound 1 6

Figure S9: HMBC spectrum of compound 1 7

Figure S10: NOESY spectrum of compound 1 7

Figure S11: HRESIMS of compound 2/3 8

Figure S12: UV Spectrum of compound 2/3. 8

Figure S13: IR Spectrum of compound 2/3. 9

Figure S14: ^{1}H NMR (Acetone-d_6, 600 MHz) spectrum of compound 2/3 10

Figure S15: ^{1}H–^{1}H COSY spectrum of compound 2/3 11

Figure S16: ^{13}C NMR (Acetone-d_6, 150 MHz) spectrum of compound 2/3 12

Figure S17: DEPT 135 spectrum of compound 2/3 13

Figure S18: HSQC spectrum of compound 2/3 13

Figure S19: HMBC spectrum of compound 2/3 14

Figure S20: NOESY spectrum of compound 2/3 14

Figure S21: HRESIMS of compound 4 15

Figure S22: UV Spectrum of compound 4 15

Figure S23: IR Spectrum of compound 4 16

Figure S24: ^{1}H NMR (DMSO-d_6, 600 MHz) spectrum of compound 4 16

Figure S25: ^{1}H–^{1}H COSY spectrum of compound 4 17
Figure S26: ^{13}C NMR (DMSO-d_6, 150 MHz) spectrum of compound 4 17
Figure S27: DEPT 135 spectrum of compound 4 18
Figure S28: HSQC spectrum of compound 4 18
Figure S29: HMBC spectrum of compound 4 19
Figure S30: NOESY spectrum of compound 4 19
Figure S31: ^{1}H NMR (DMSO-d_6, 600 MHz) spectrum of compound 5 20
Figure S32: ^{13}C NMR (DMSO-d_6, 150 MHz) spectrum of compound 5 20
Figure S33: ^{1}H NMR (DMSO-d_6, 600 MHz) spectrum of compound 6 21
Figure S34: ^{13}C NMR (DMSO-d_6, 150 MHz) spectrum of compound 6 21
Figure S35: ^{1}H NMR (Acetone, 500 MHz) spectrum of compound 7 22
Figure S36: ^{13}C NMR (Acetone, 125 MHz) spectrum of compound 7 22
Figure S37: ^{1}H NMR (DMSO-d_6, 500 MHz) spectrum of compound 8 23
Figure S38: ^{13}C NMR (DMSO-d_6, 125 MHz) spectrum of compound 8 23
Figure S39: ^{1}H NMR (DMSO-d_6, 500 MHz) spectrum of compound 9 24
Figure S40: ^{13}C NMR (DMSO-d_6, 125 MHz) spectrum of compound 9 24
Figure S41: ^{1}H NMR (Acetone, 500 MHz) spectrum of compound 10 25
Figure S42: ^{13}C NMR (Acetone, 125 MHz) spectrum of compound 10 25
Figure S43: ^{1}H NMR ($CDCl_3$, 600 MHz) spectrum of compound 11+12 26
Figure S44: ^{13}C NMR ($CDCl_3$, 150 MHz) spectrum of compound **11+1** 26
Figure S45: ^{1}H NMR (Pyridine-d_5, 600 MHz) spectrum of compound 13 27
Figure S46: ^{13}C NMR (Pyridine-d_5, 150 MHz) spectrum of compound 13 27
Figure S47: ^{1}H NMR (Acetone, 600 MHz) spectrum of compound 14 28
Figure S48: ^{13}C NMR (Acetone, 150 MHz) spectrum of compound 14 28
Figure S49: ^{1}H NMR ($CDCl_3$, 600 MHz) spectrum of compound 15 29
Figure S50: ^{13}C NMR ($CDCl_3$, 150 MHz) spectrum of compound 15 29

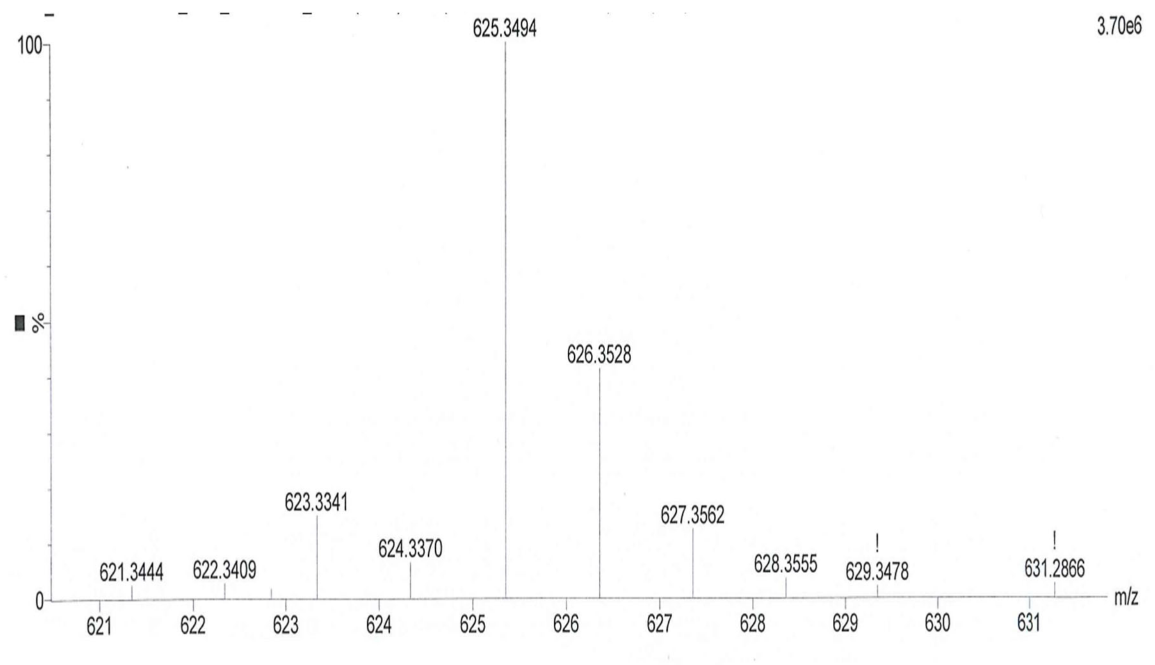

Figure S1: HRESIMS of compound **1.**

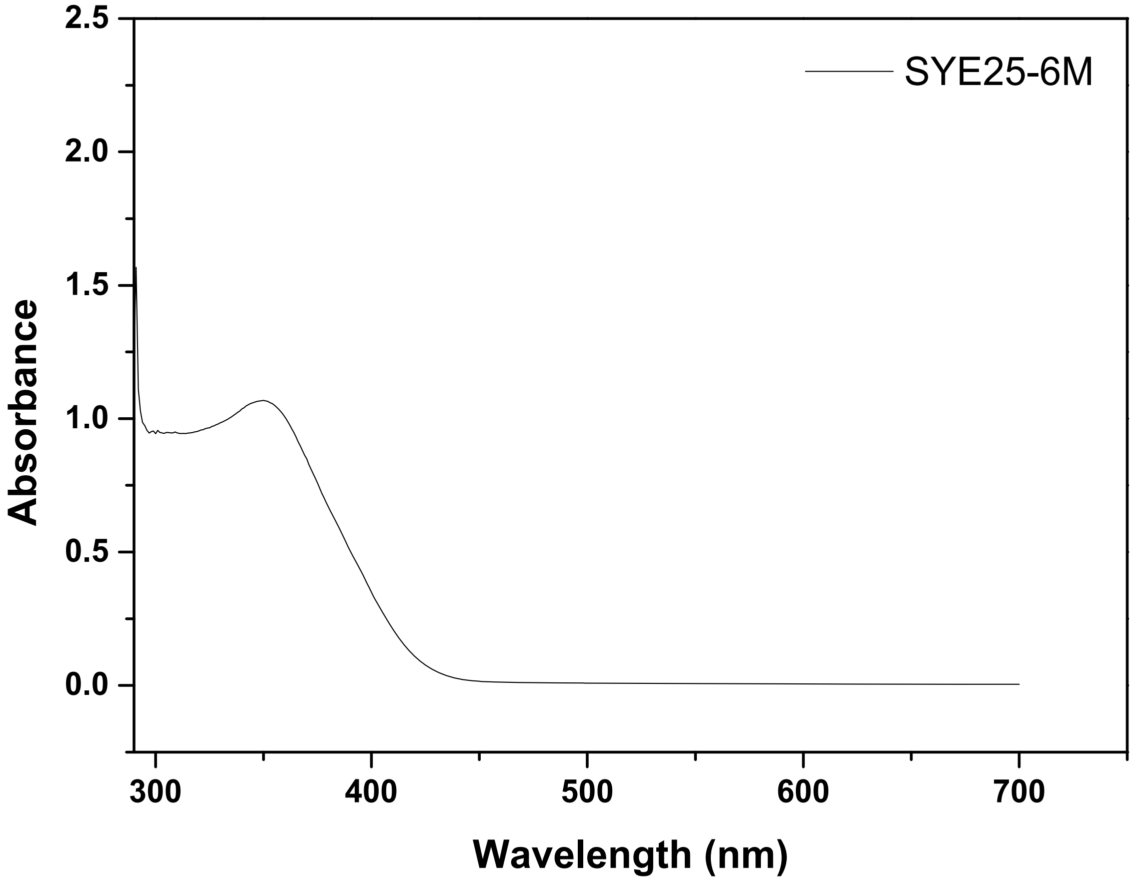

Figure S2: UV Spectrum of compound **1.**

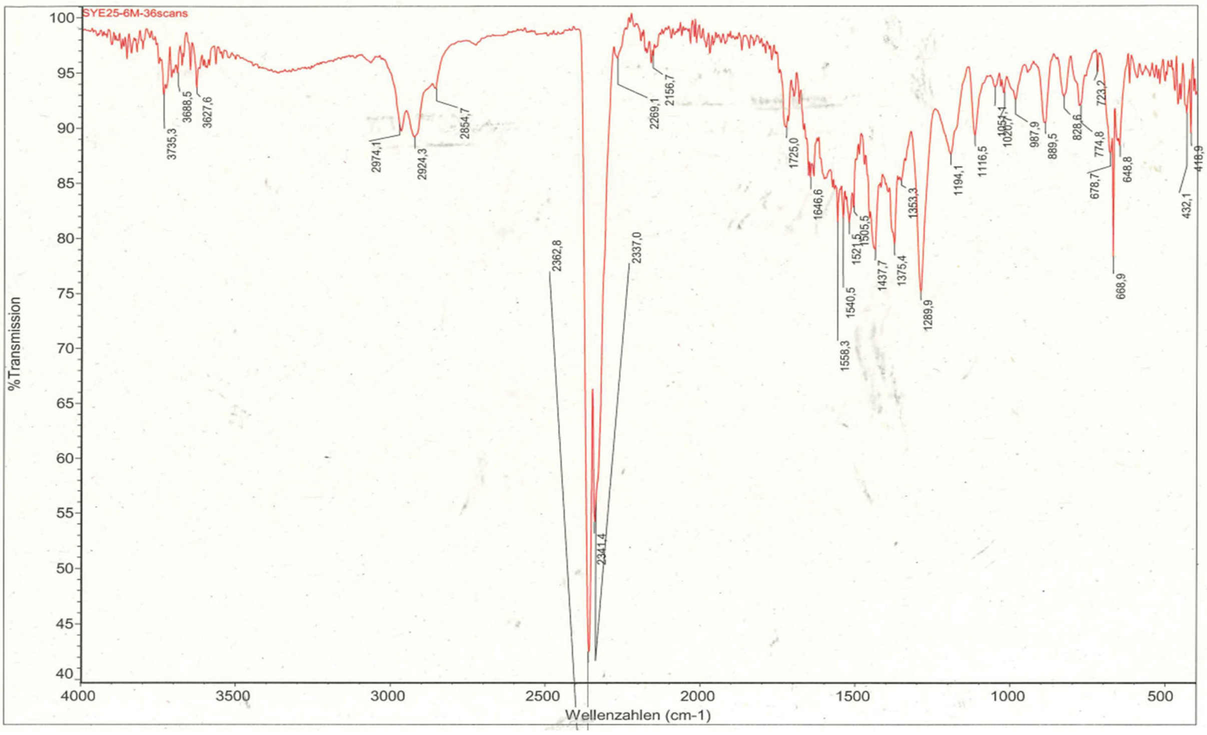

Figure S3: IR Spectrum of compound **1.**

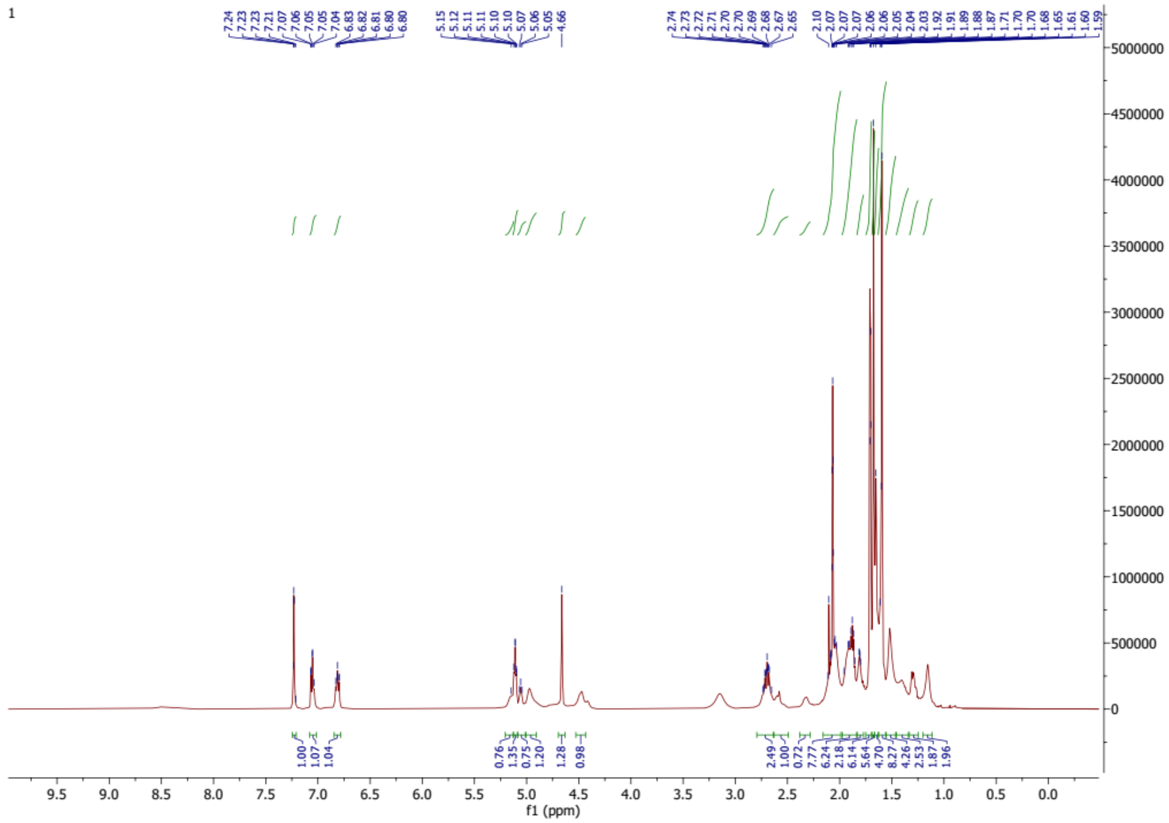

Figure S4: ^{1}H NMR (Acetone-d_6, 600 MHz) spectrum of compound **1**

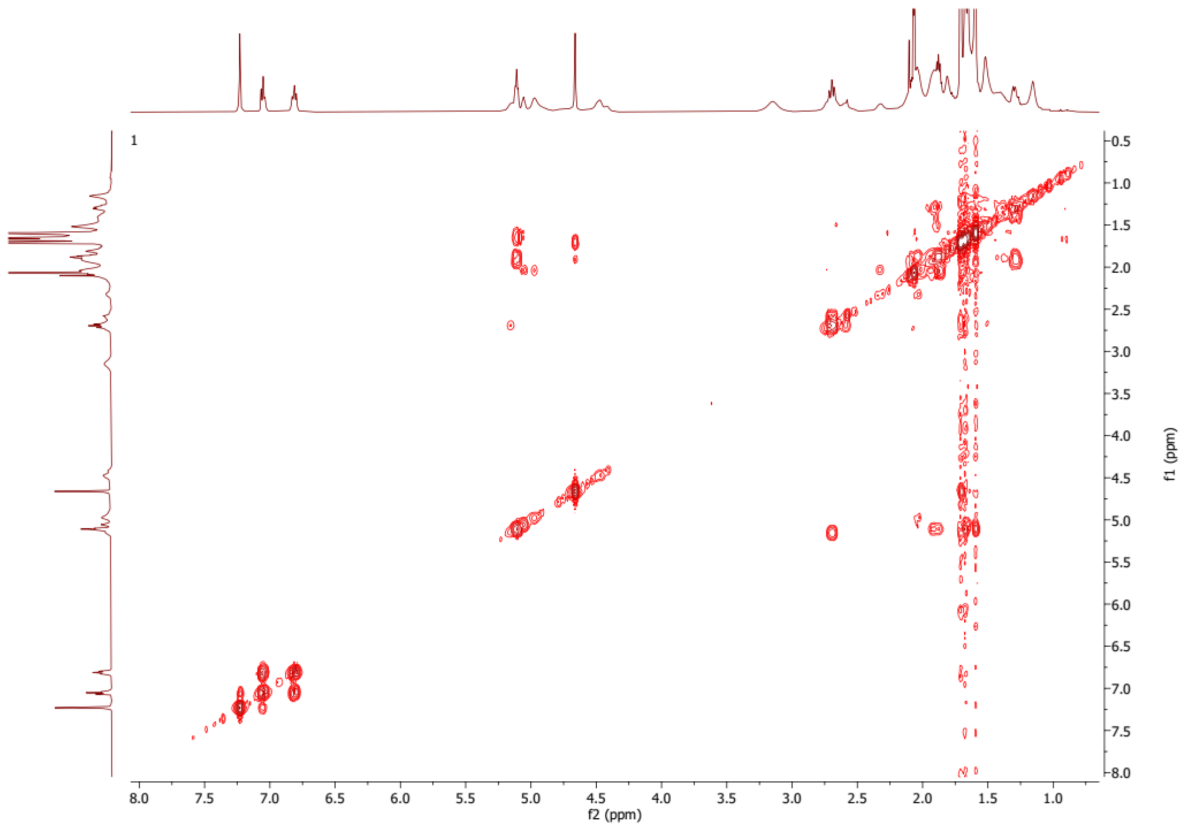

Figure S5: ^{1}H–^{1}H COSY spectrum of compound **1**

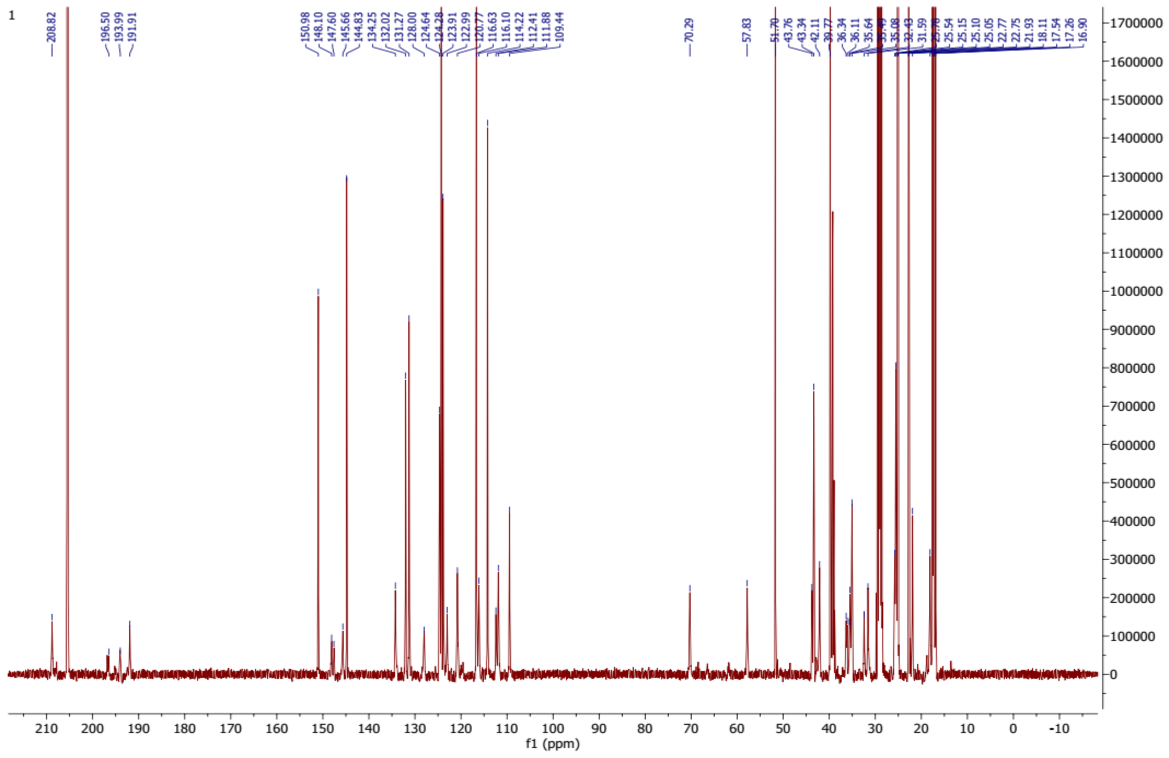

Figure S6: ^{13}C NMR (Acetone-d_6, 150 MHz) spectrum of compound **1**

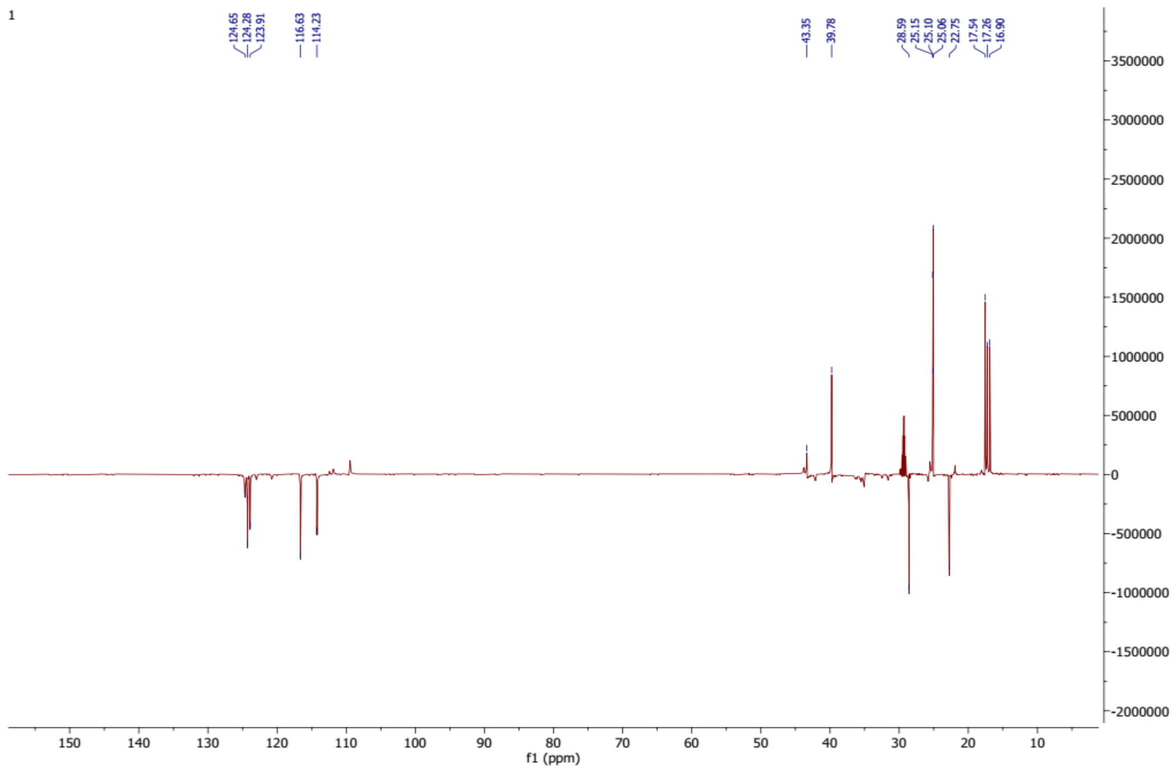

Figure S7: DEPT 135 NMR spectrum of compound **1**

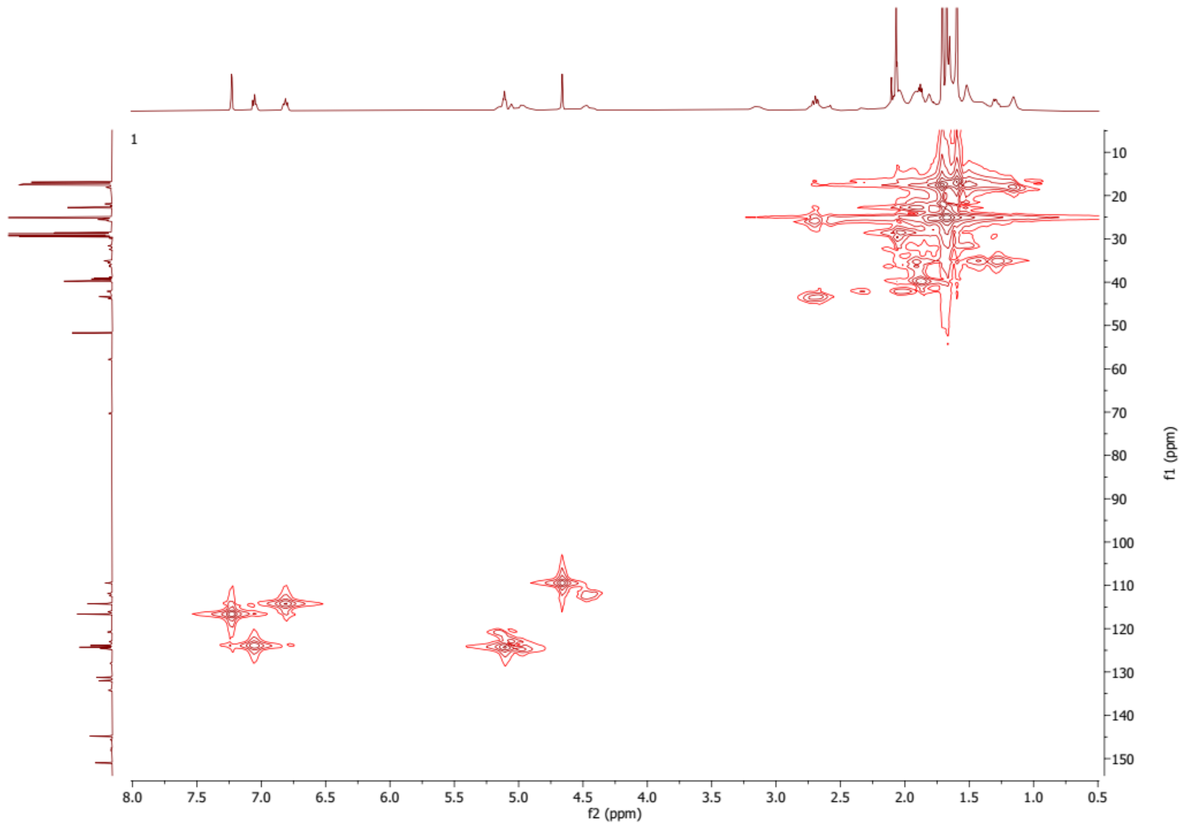

Figure S8: HSQC spectrum of compound **1**

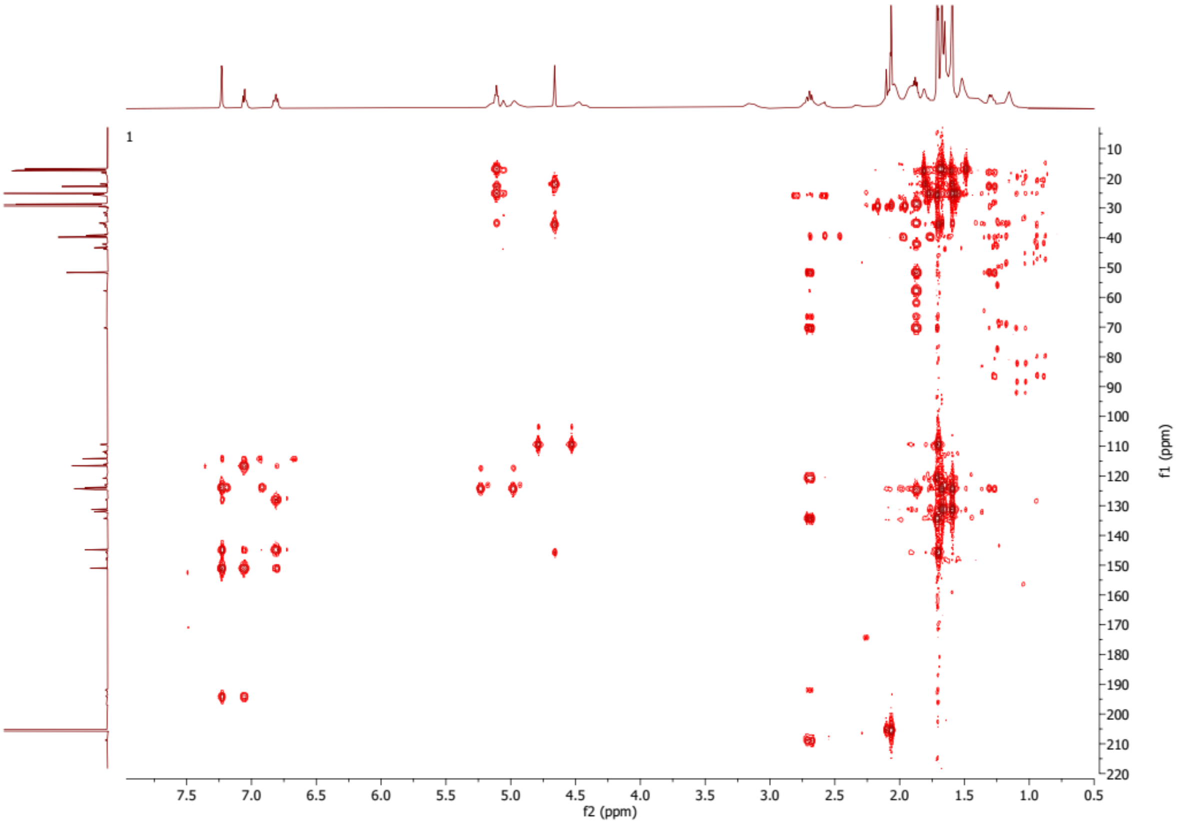

Figure S9: HMBC spectrum of compound **1**

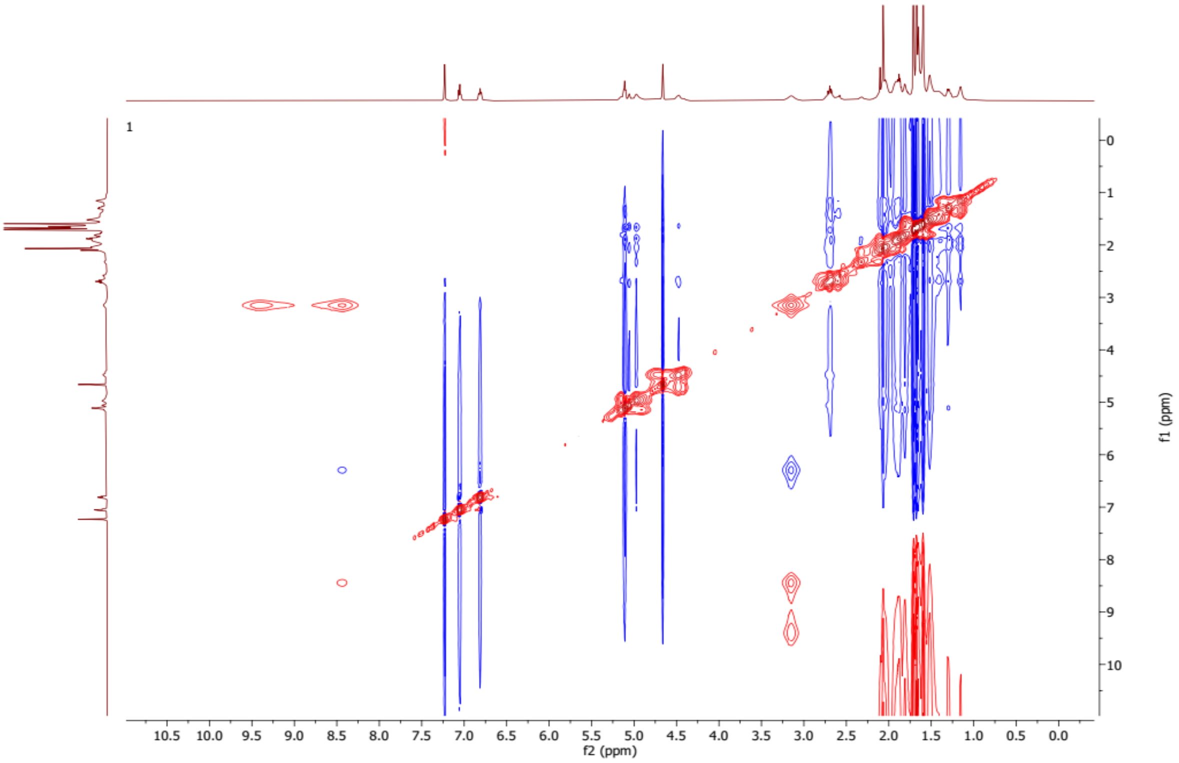

Figure S10: NOESY spectrum of compound **1**

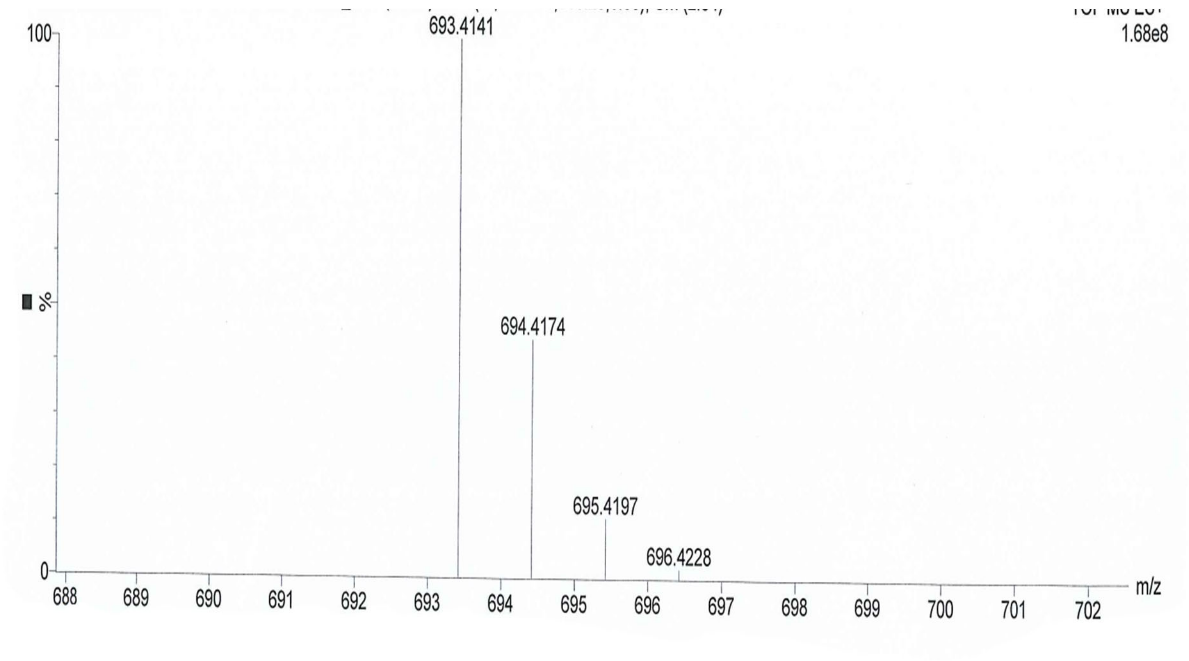

Figure S11: HRESIMS of compound **2/3.**

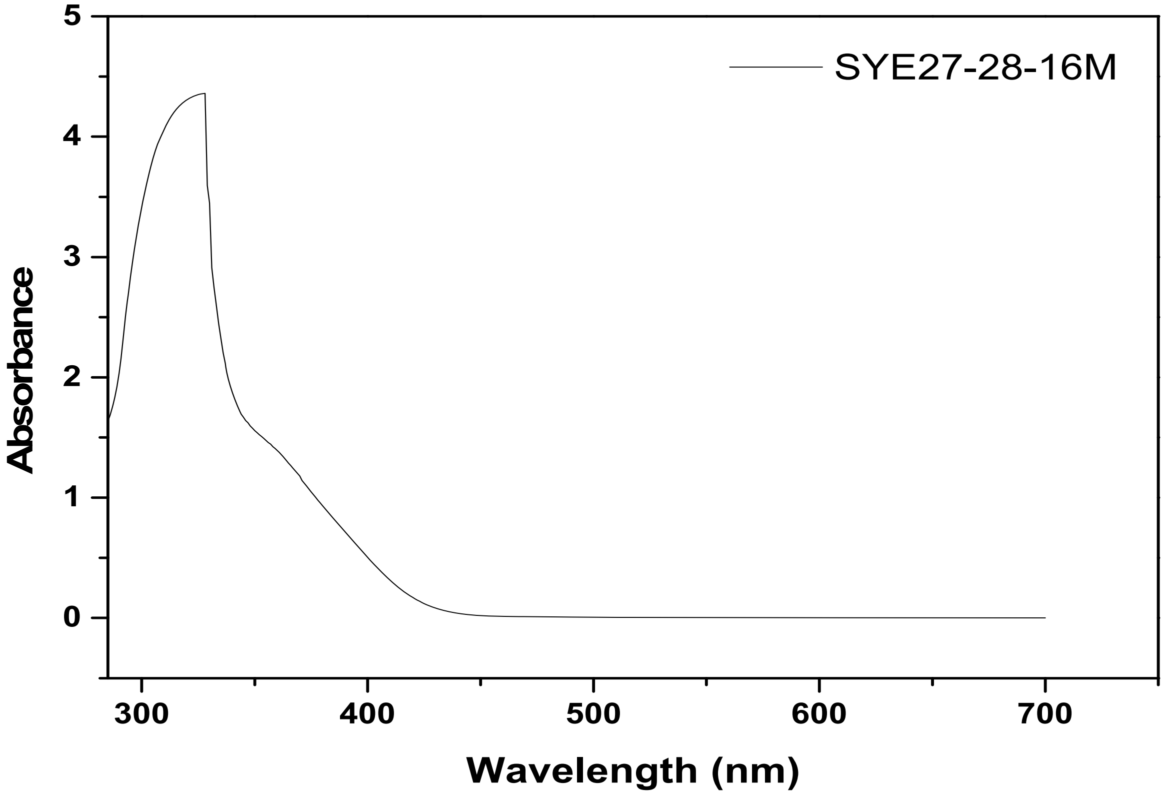

Figure S12: UV Spectrum of compound **2/3.**

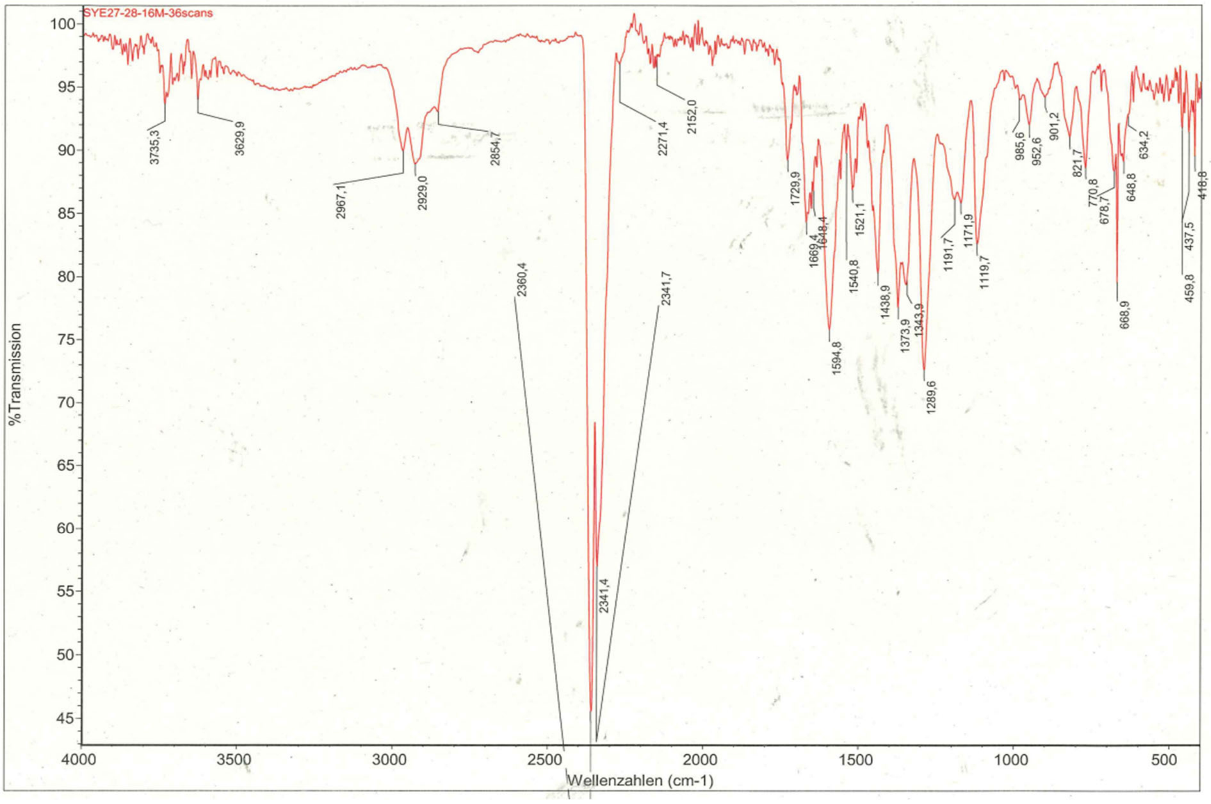

Figure S13: IR Spectrum of compound **2/3.**

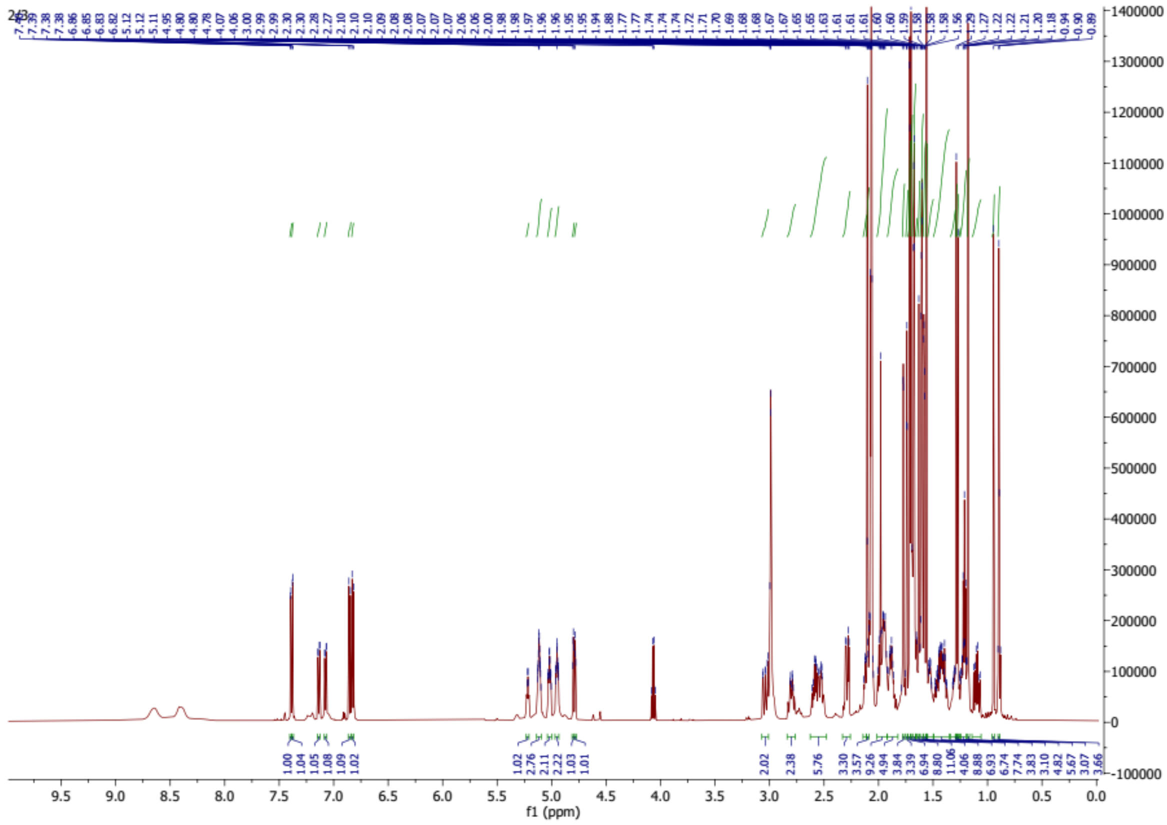

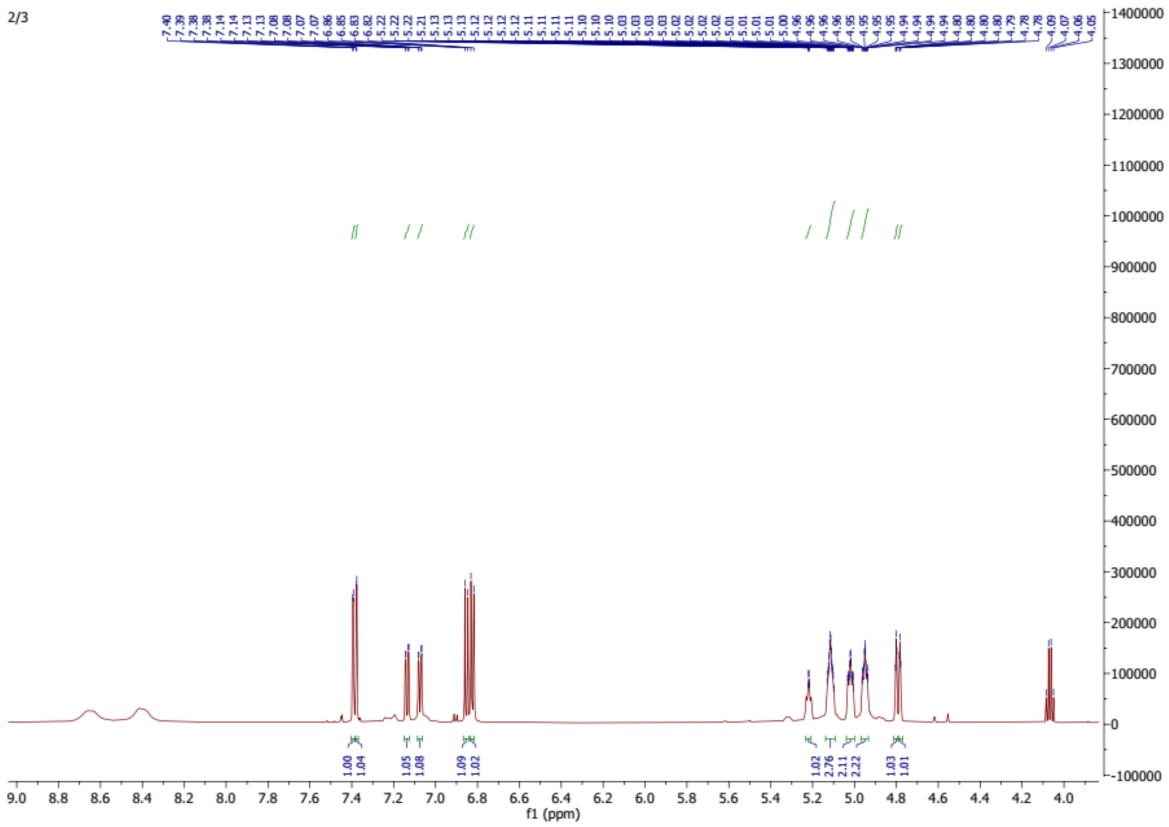

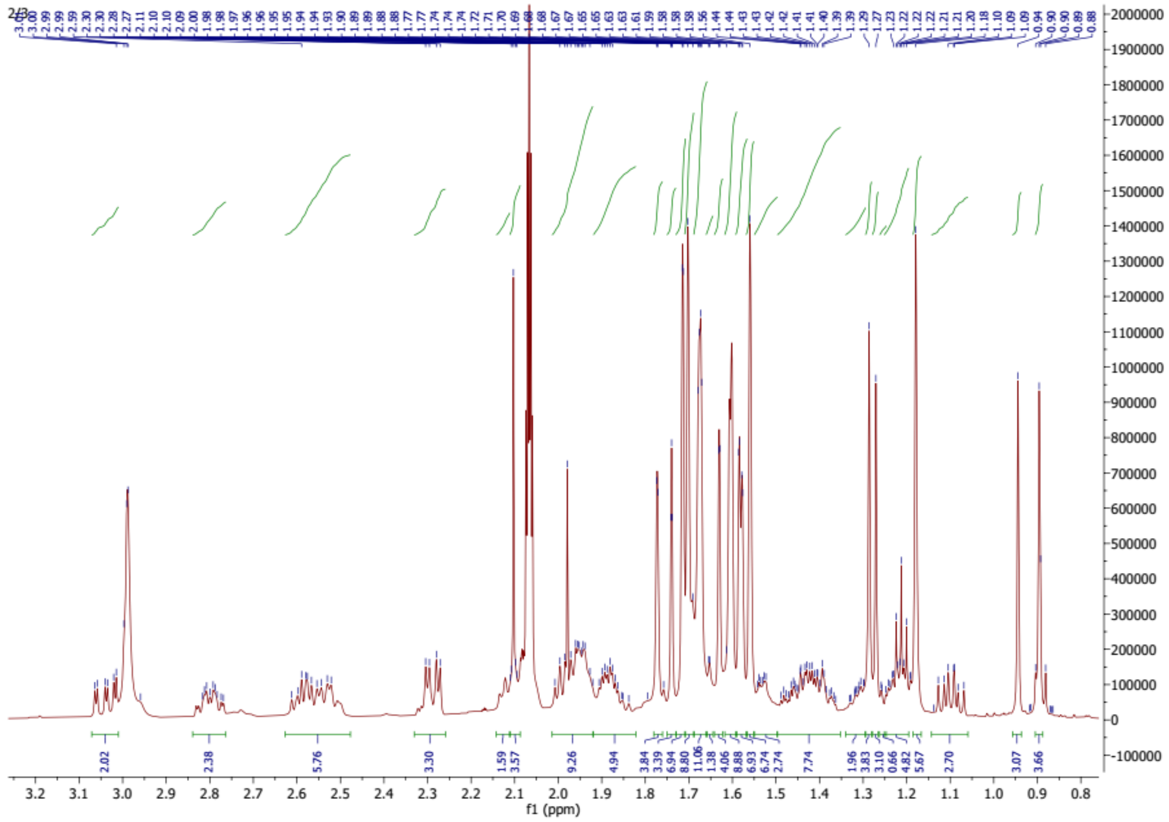

Figure S14: ^{1}H NMR (Acetone-d_6, 600 MHz) spectrum of compound **2/3**

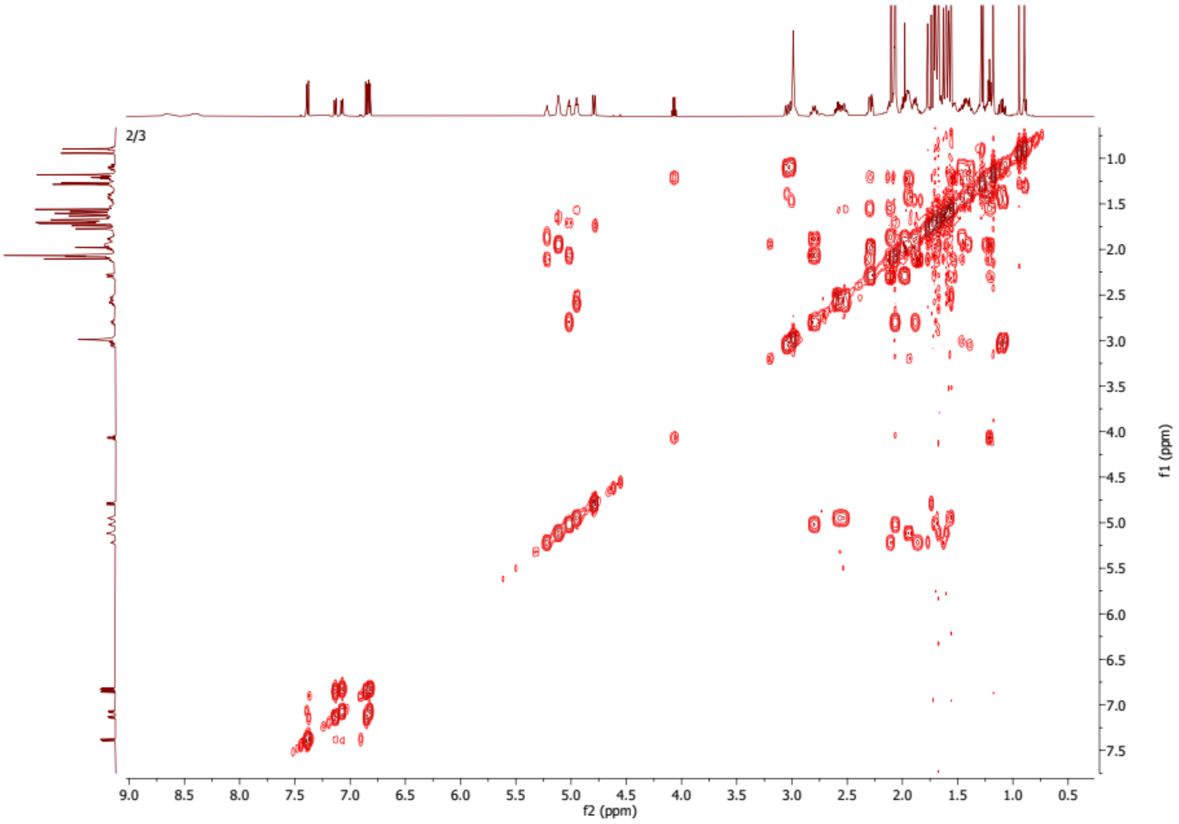

Figure S15: ^{1}H–^{1}H COSY spectrum of compound **2/3**

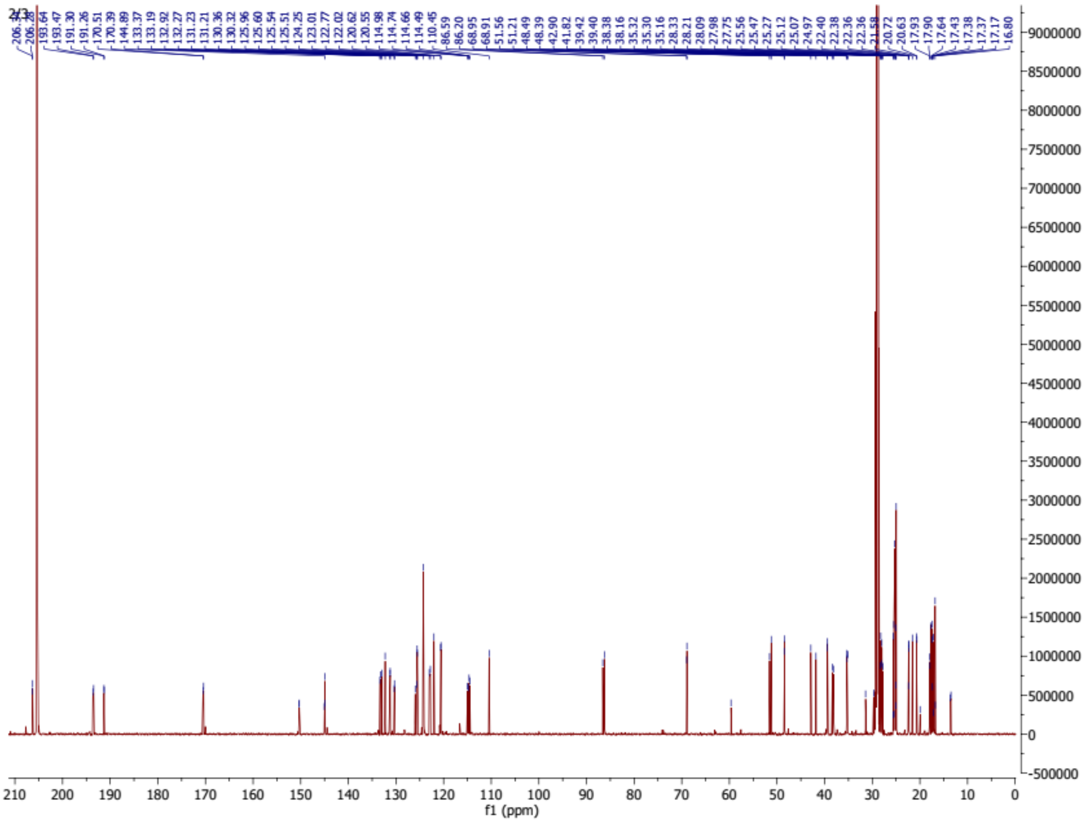

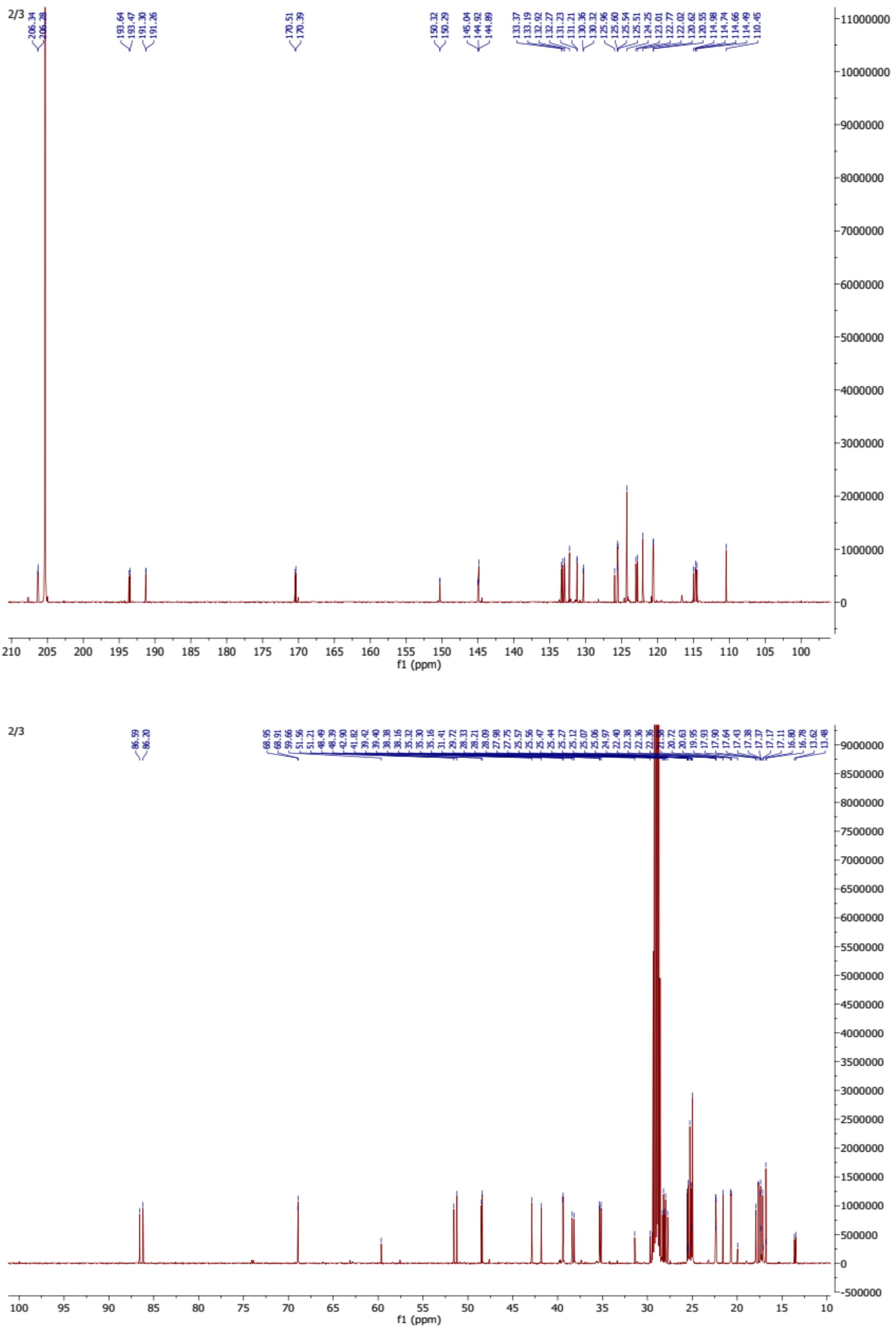

Figure S16: ^{13}C NMR (Acetone-d_6, 150 MHz) spectrum of compound **2/3**

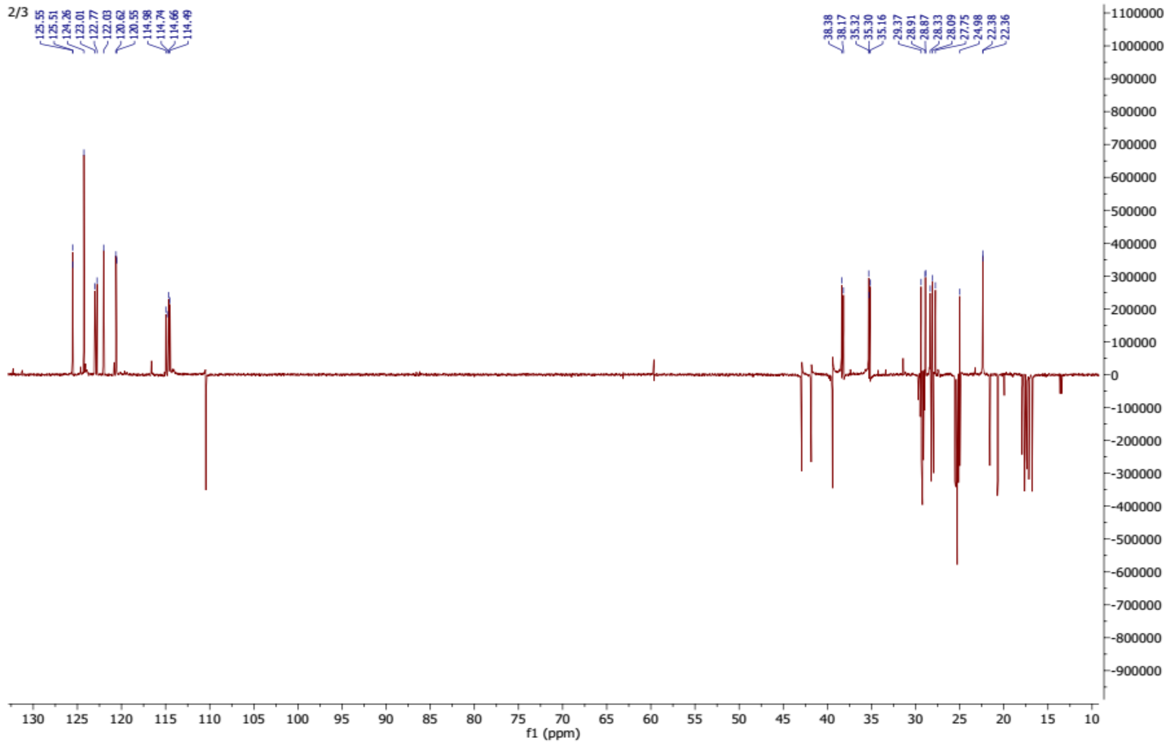

Figure S17: DEPT 135 spectrum of compound **2/3**

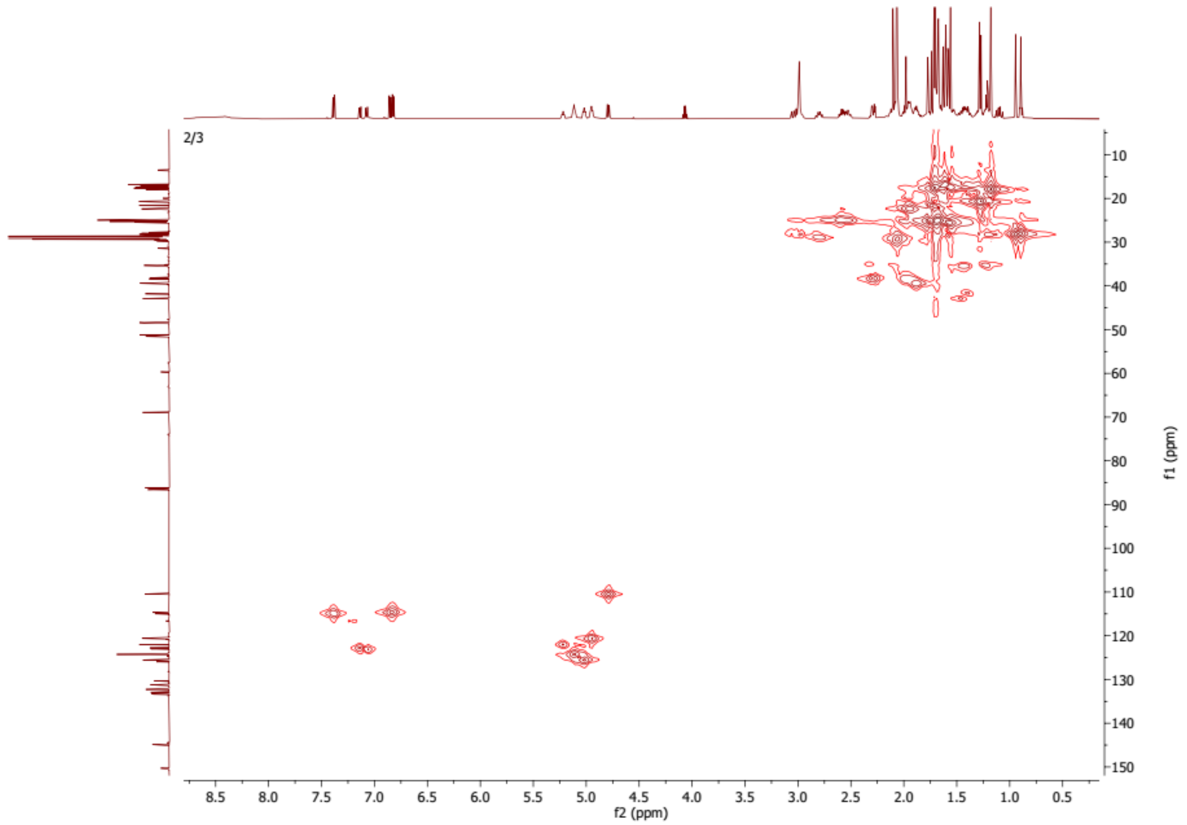

Figure S18: HSQC spectrum of compound **2/3**

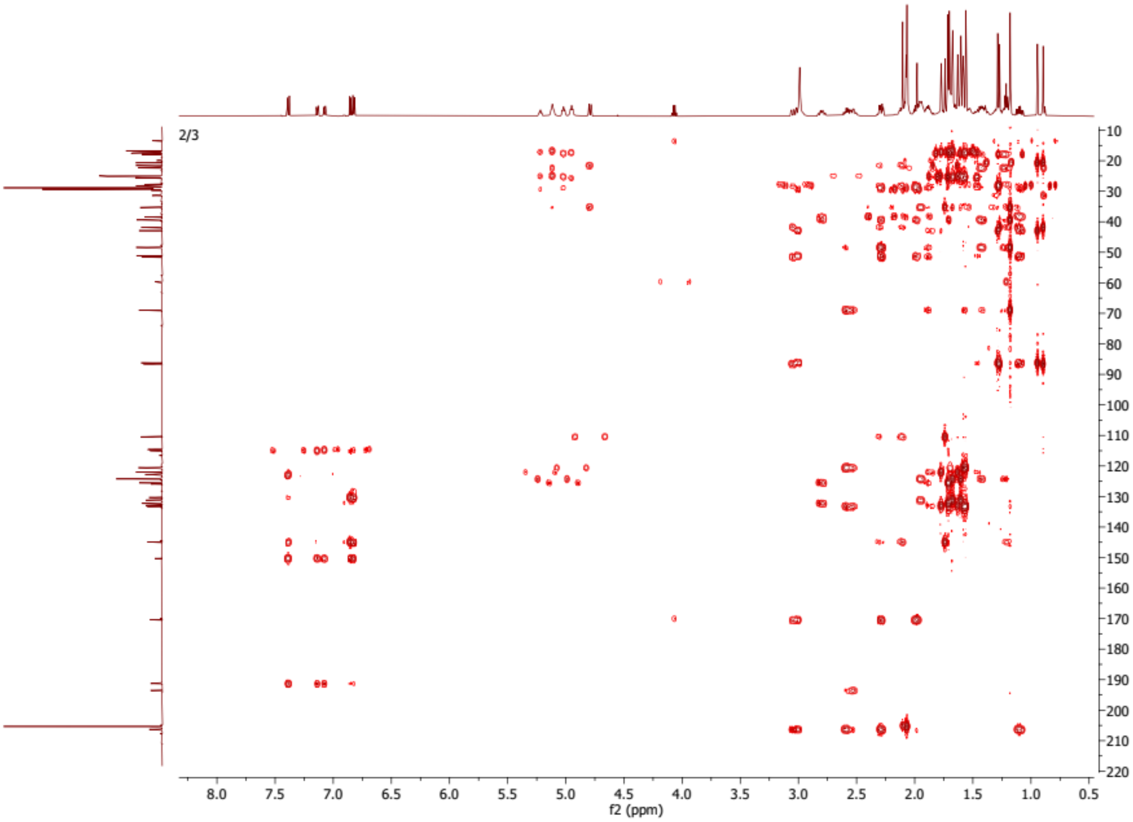

Figure S19: HMBC spectrum of compound **2/3**

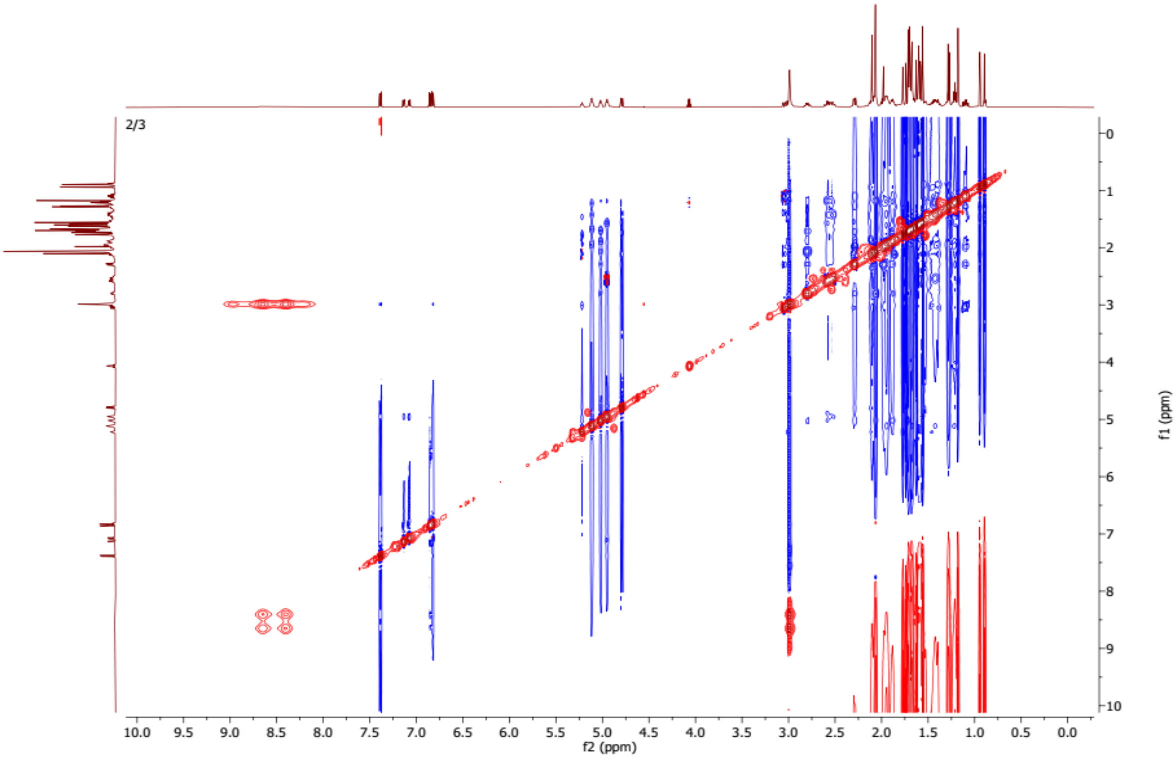

Figure S20: NOESY spectrum of compound **2/3**

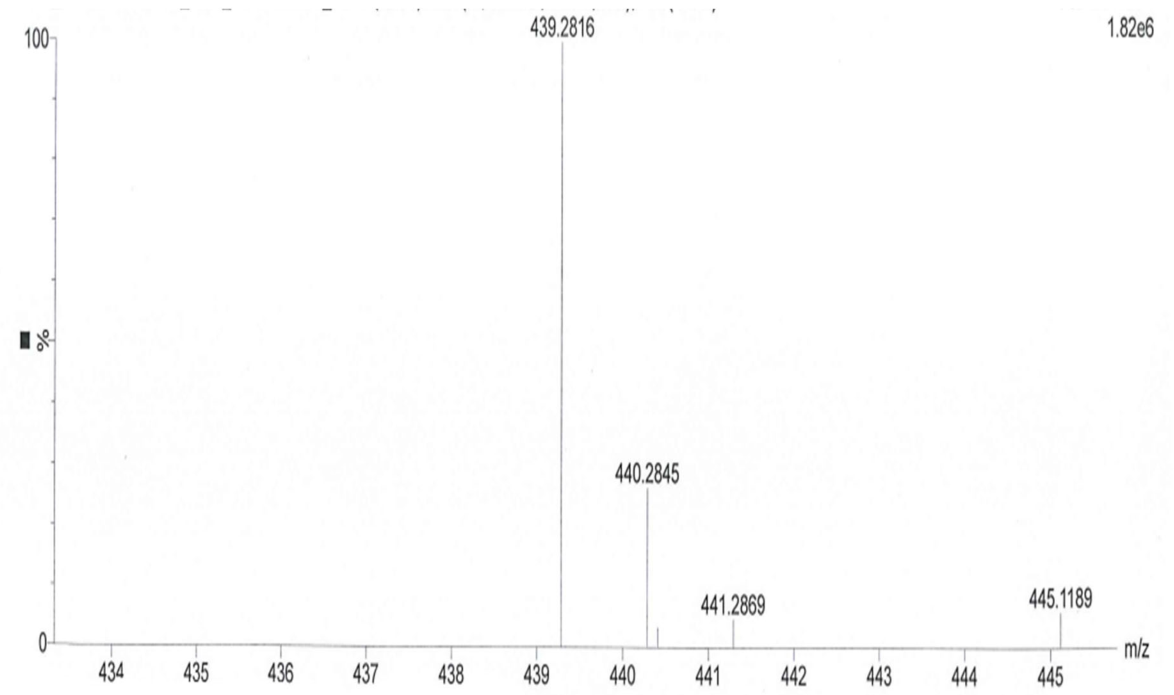

Figure S21: HRESIMS of compound **4**

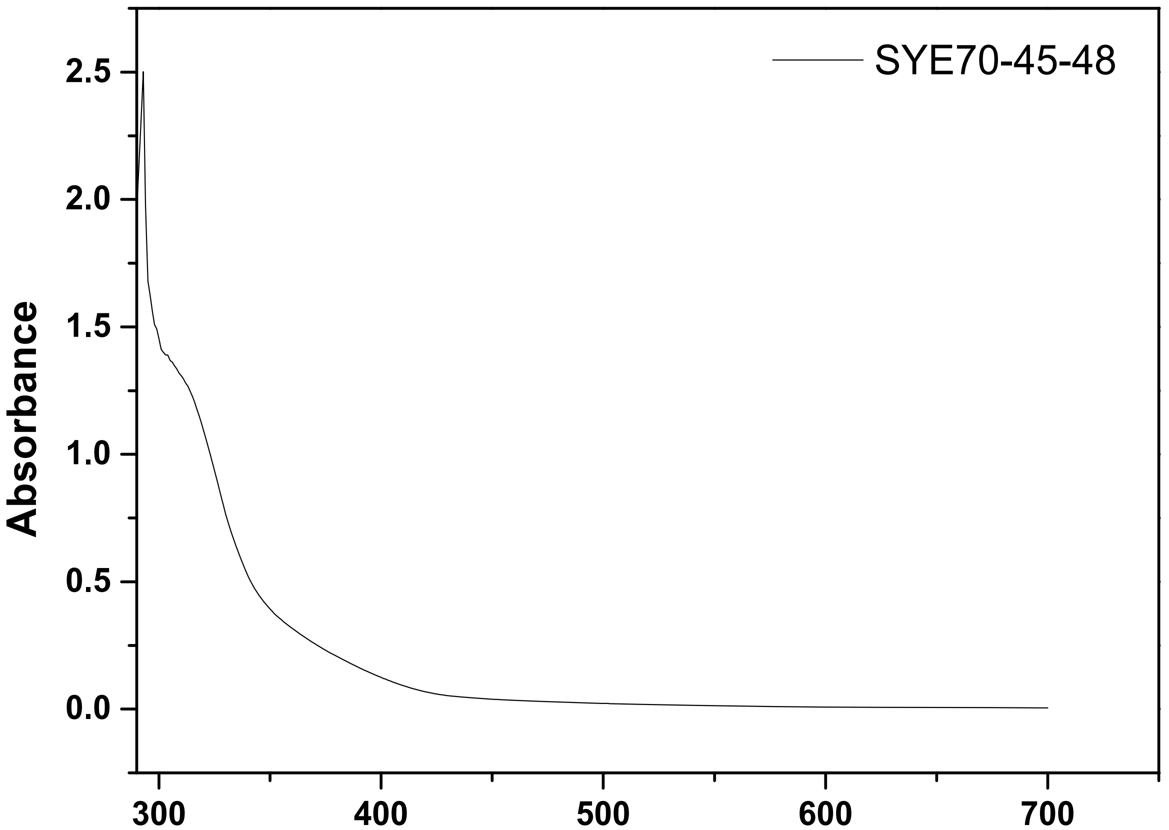

Figure S22: UV Spectrum of compound **4**

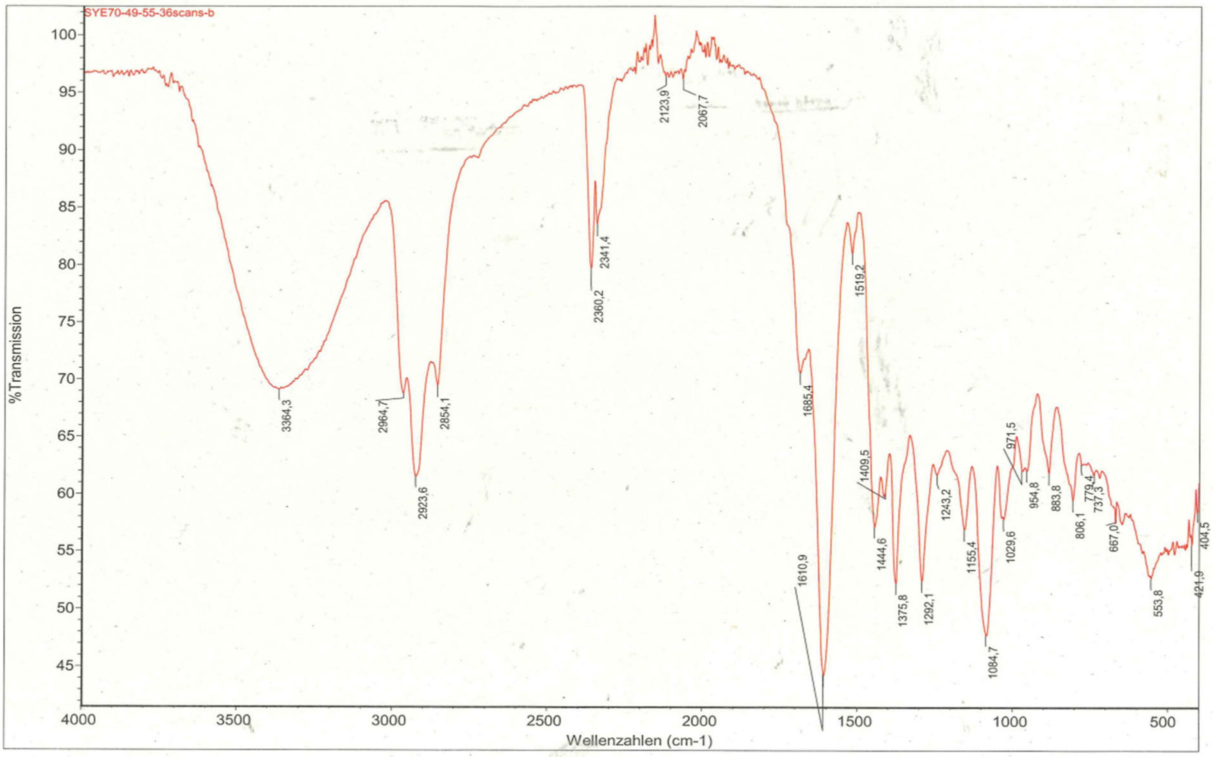

Figure S23: IR Spectrum of compound 4

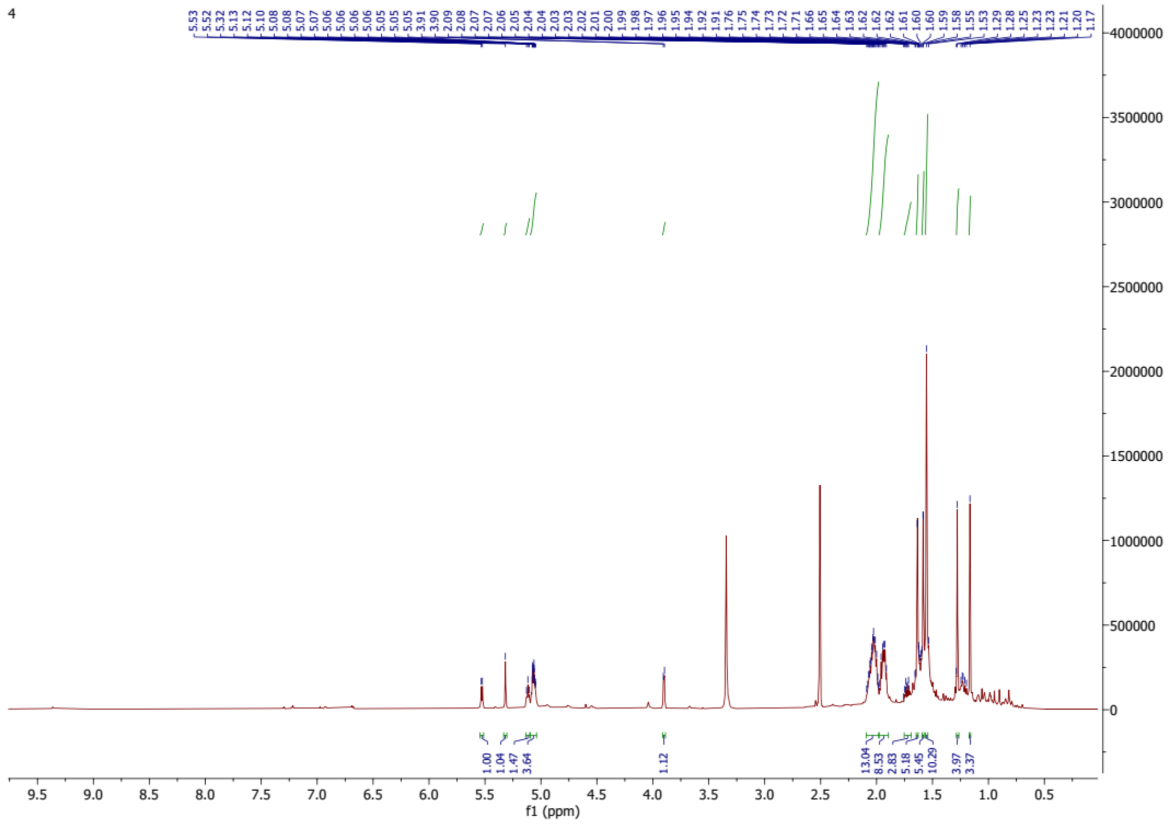

Figure S24: ^{1}H NMR (DMSO-d_6, 600 MHz) spectrum of compound **4**

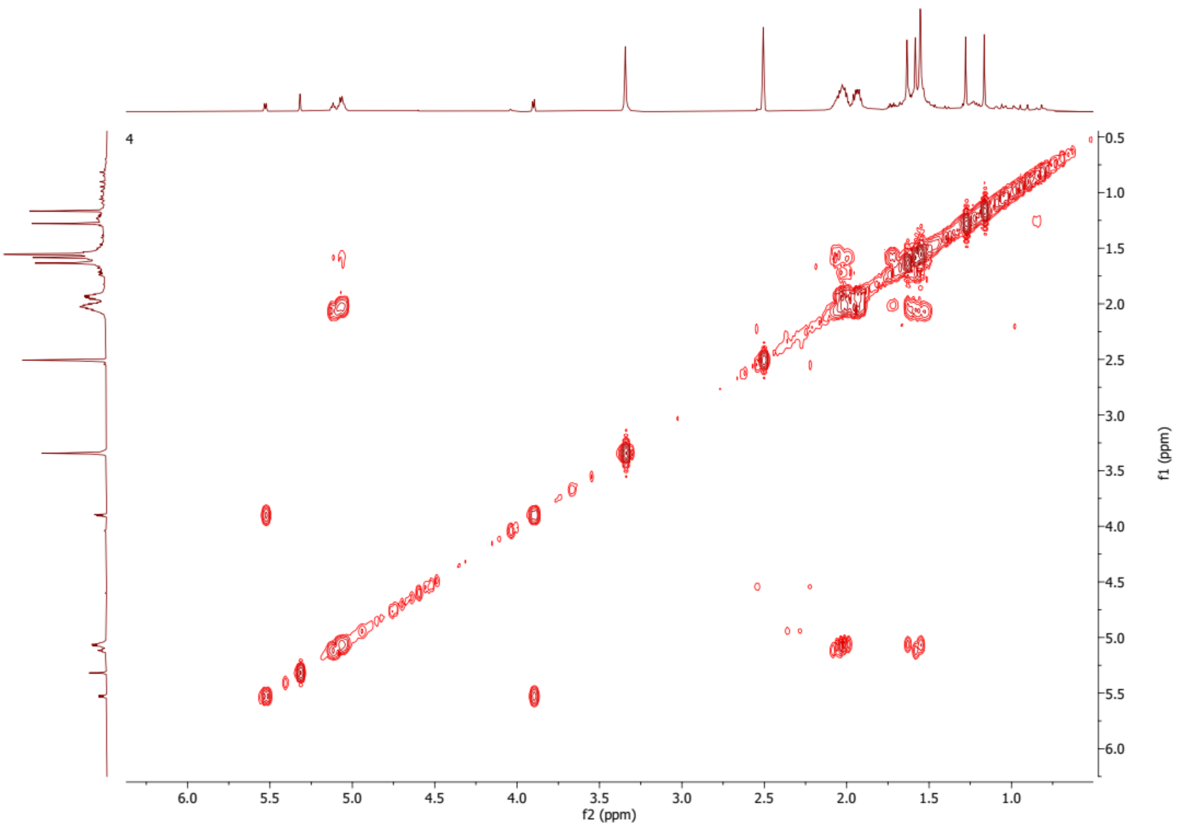

Figure S25: ^{1}H–^{1}H COSY spectrum of compound **4**

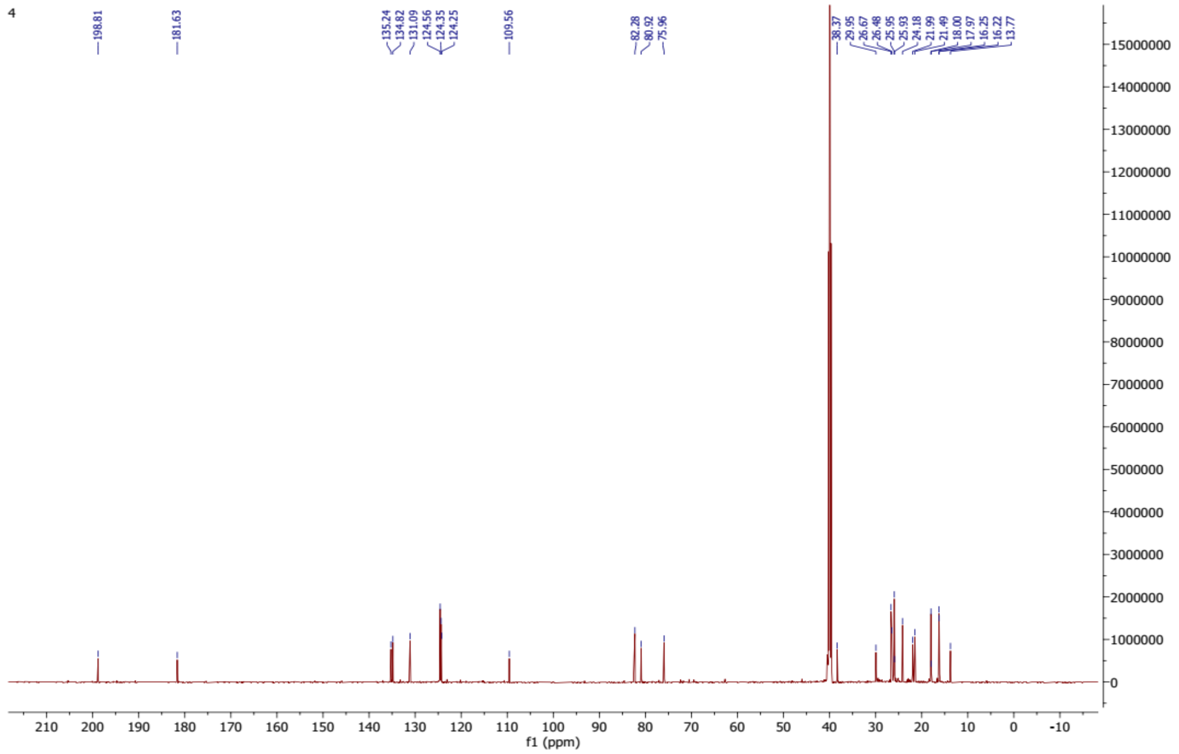

Figure S26: ^{13}C NMR (DMSO-d_6, 150 MHz) spectrum of compound **4**

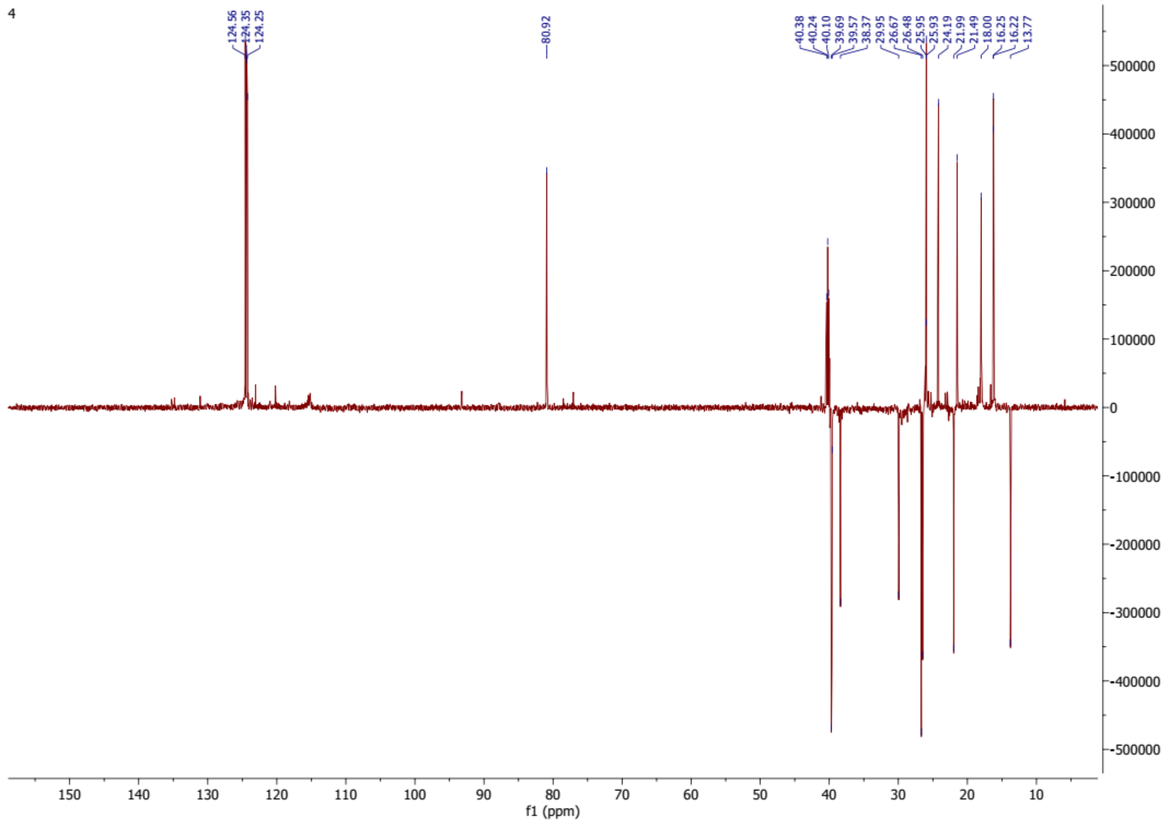

Figure S27: DEPT 135 spectrum of compound **4**

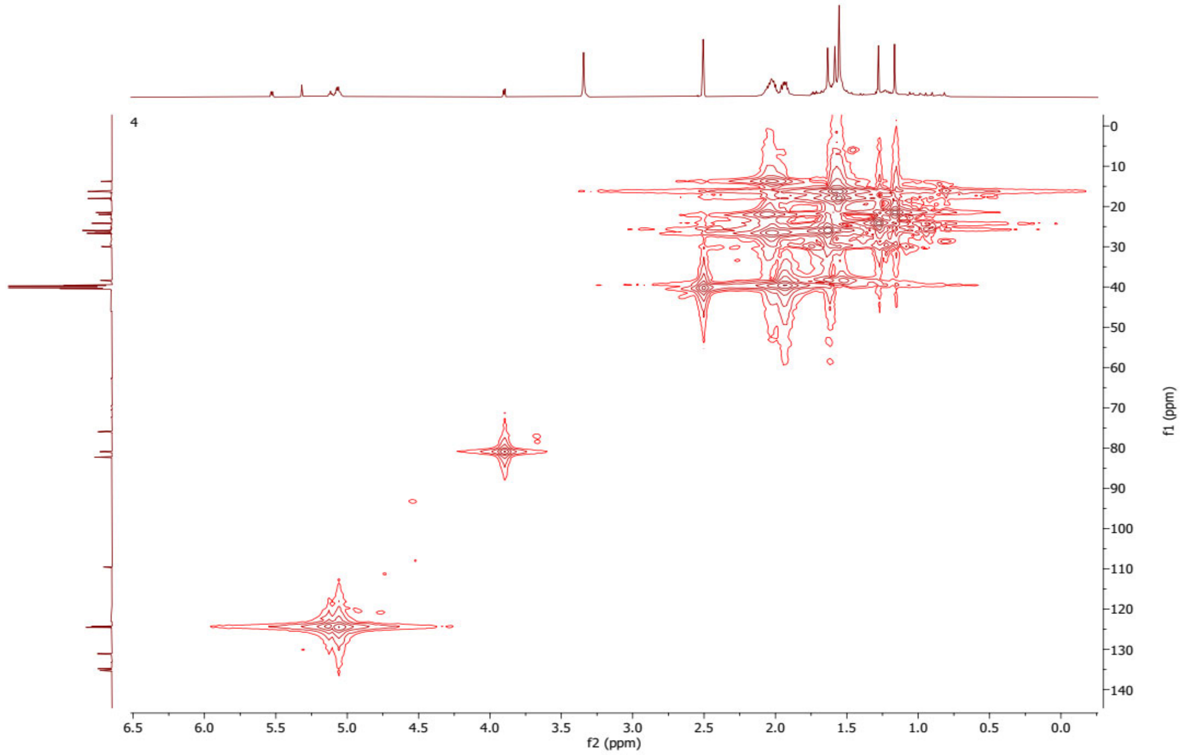

Figure S28: HSQC spectrum of compound **4**

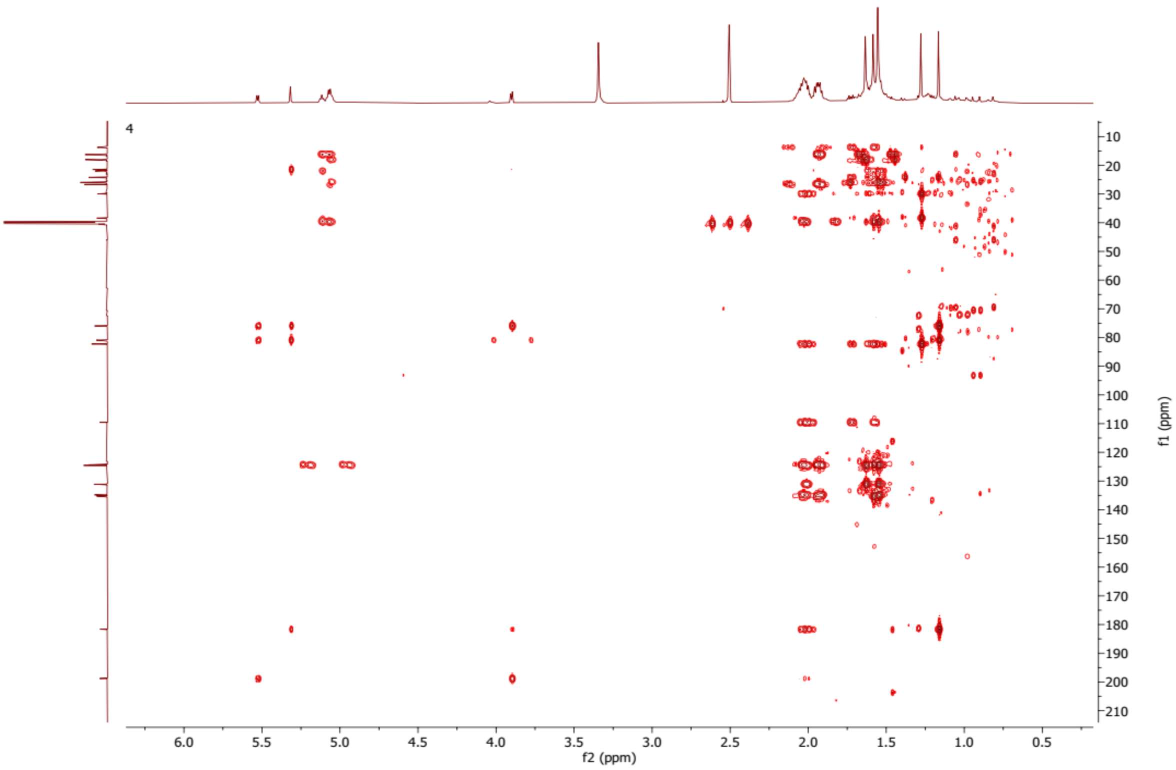

Figure S29: HMBC spectrum of compound **4**

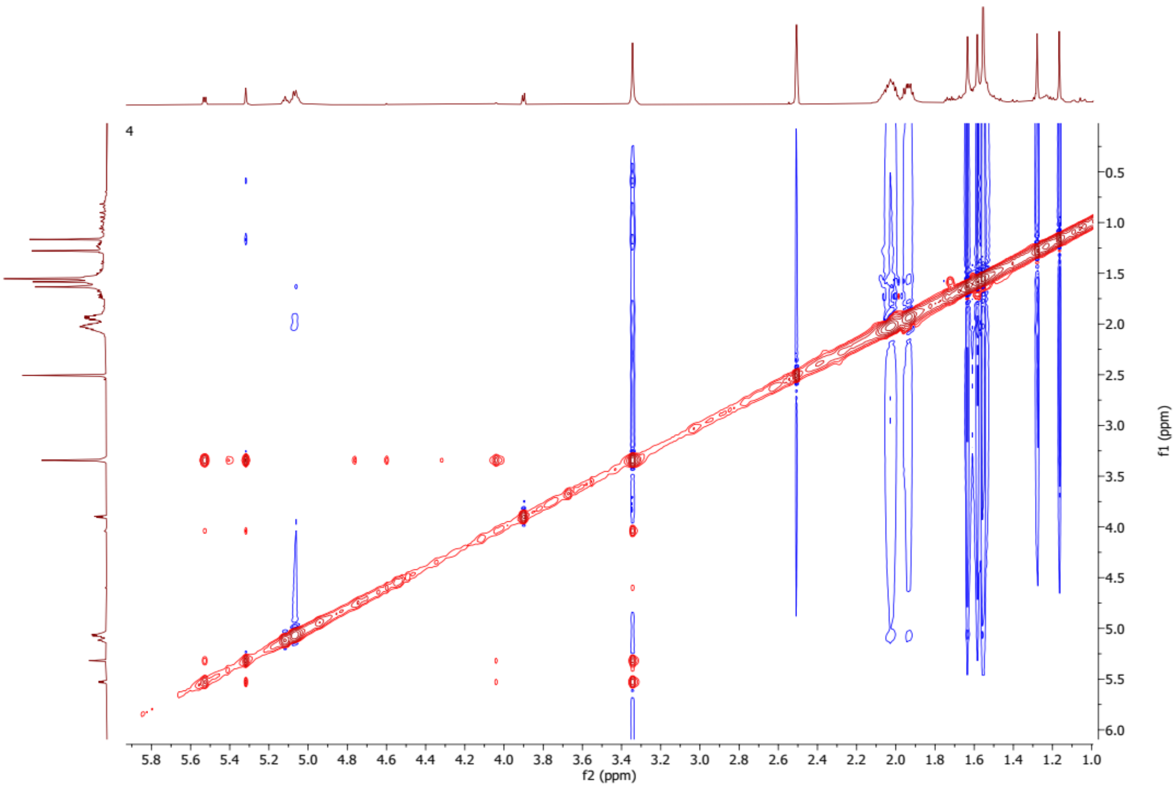

Figure S30: NOESY spectrum of compound **4**

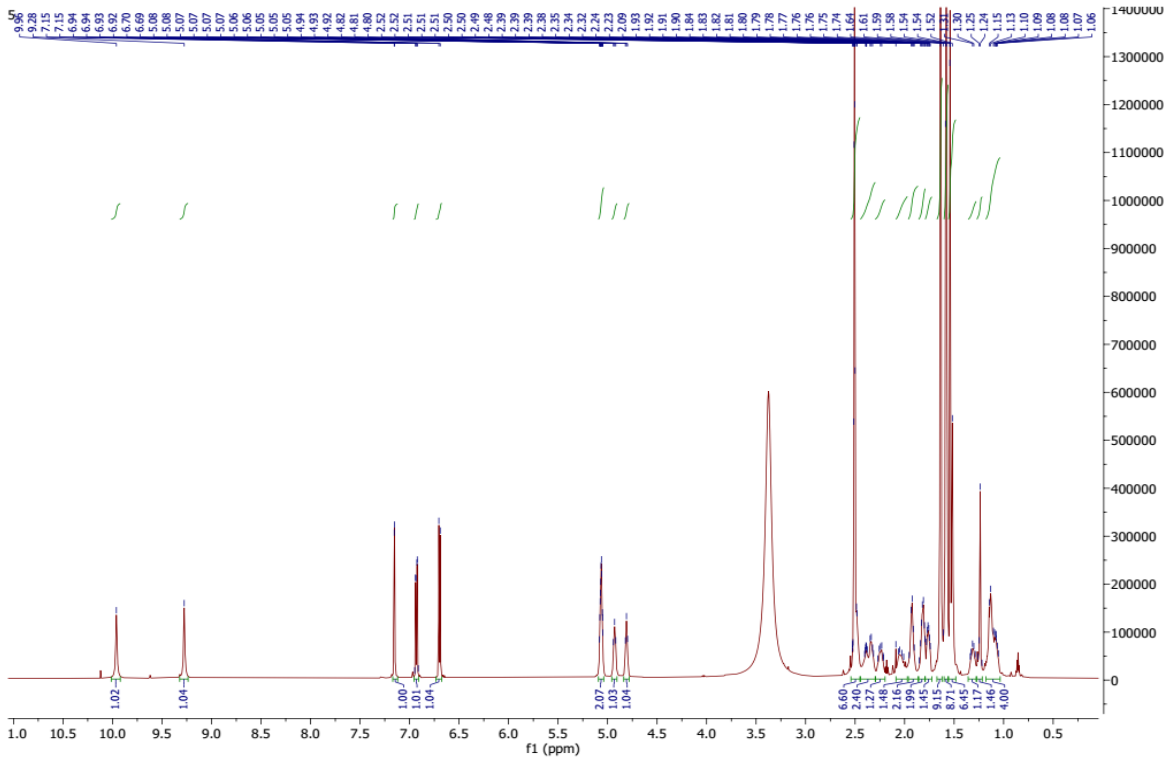

Figure S31: ^{1}H NMR (DMSO-d_6, 600 MHz) spectrum of compound **5**

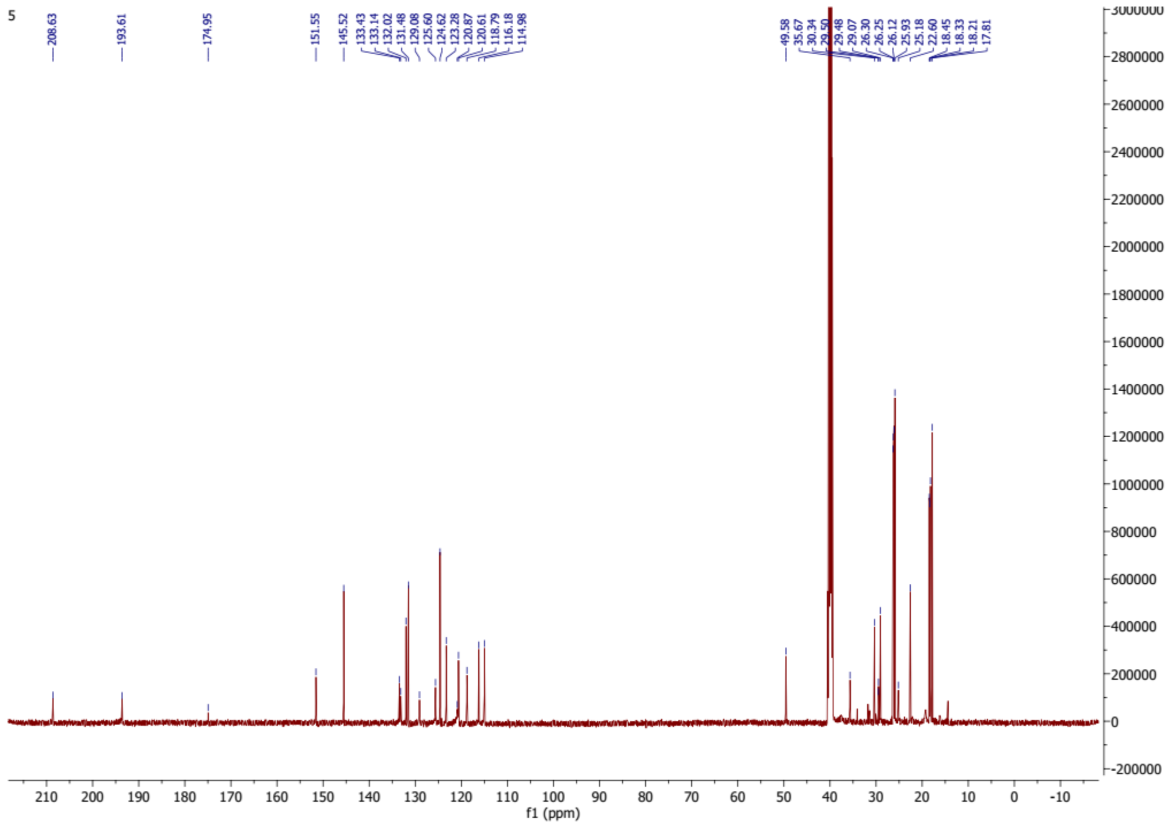

Figure S32: ^{13}C NMR (DMSO-d_6, 150 MHz) spectrum of compound **5**

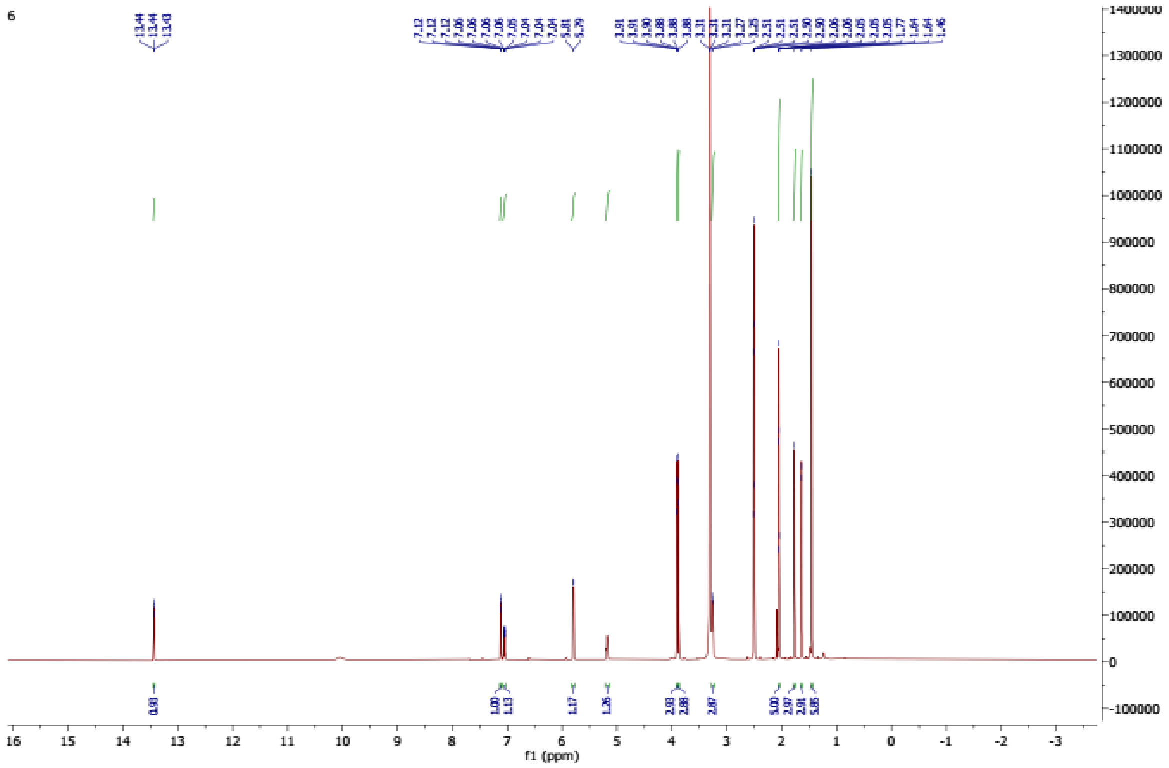

Figure S33: ^{1}H NMR (DMSO-d_6, 600 MHz) spectrum of compound **6**

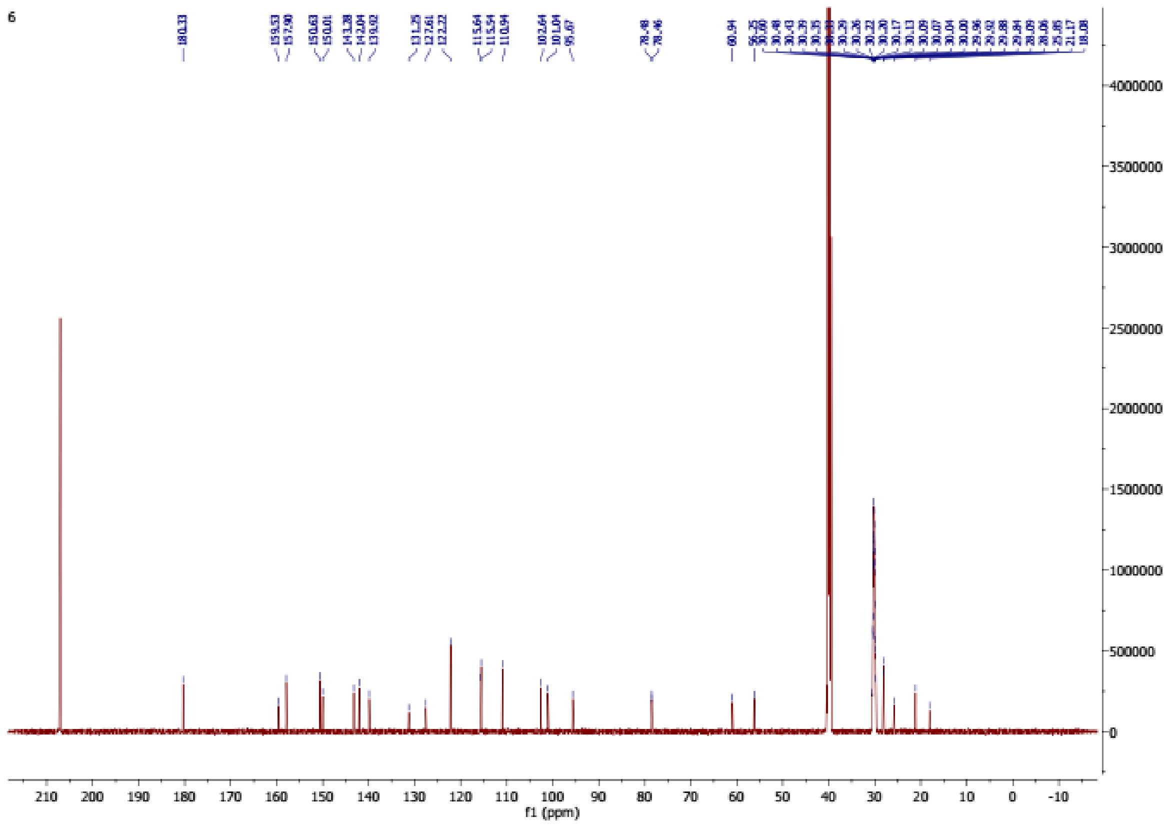

Figure S34: ^{13}C NMR (DMSO-d_6, 150 MHz) spectrum of compound **6**

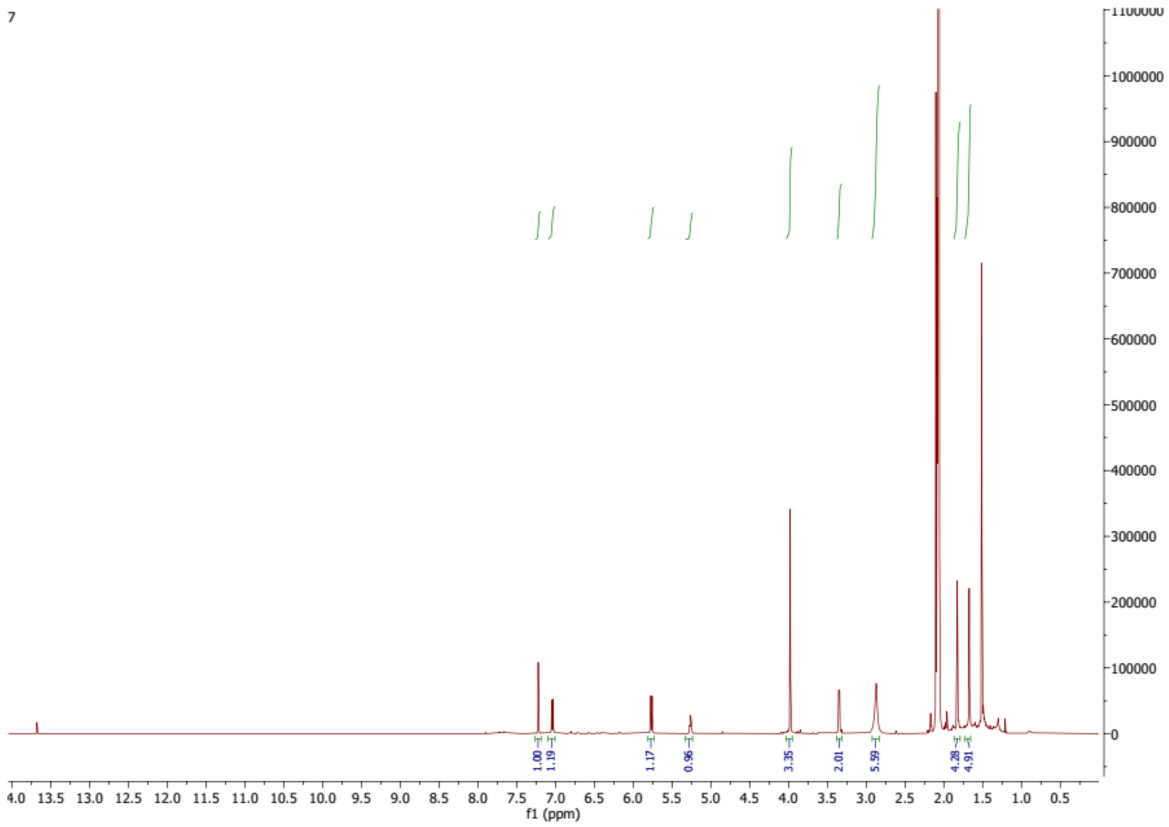

Figure S35: ^{1}H NMR (Acetone, 500 MHz) spectrum of compound **7**

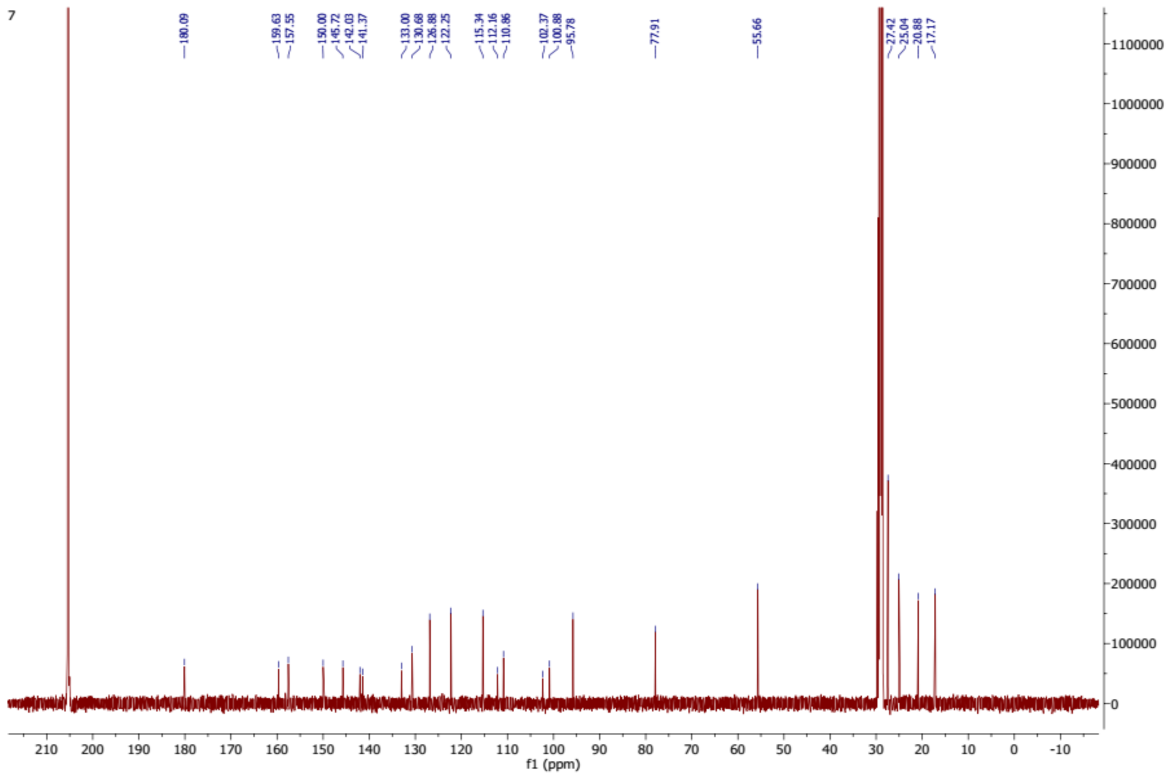

Figure S36: ^{13}C NMR (Acetone, 125 MHz) spectrum of compound **7**

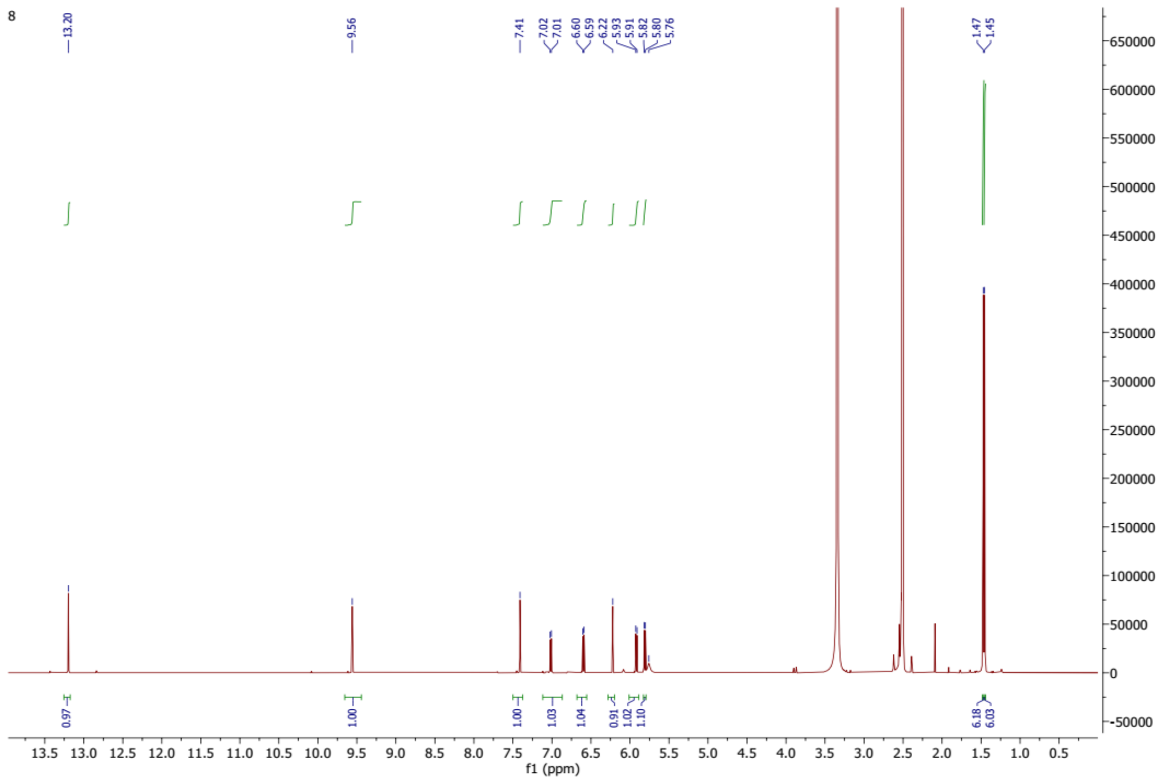

Figure S37: ^{1}H NMR (DMSO-d_6, 500 MHz) spectrum of compound **8**

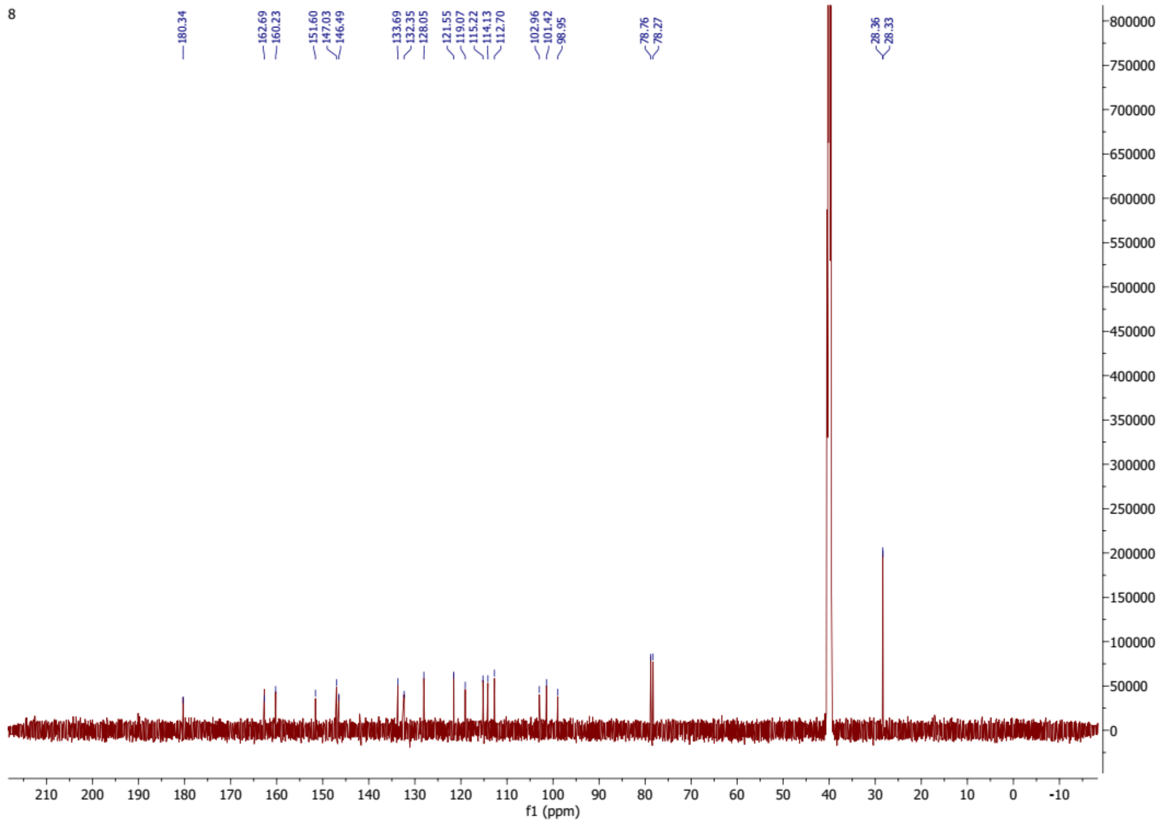

Figure S38: ^{13}C NMR (DMSO-d_6, 125 MHz) spectrum of compound **8**

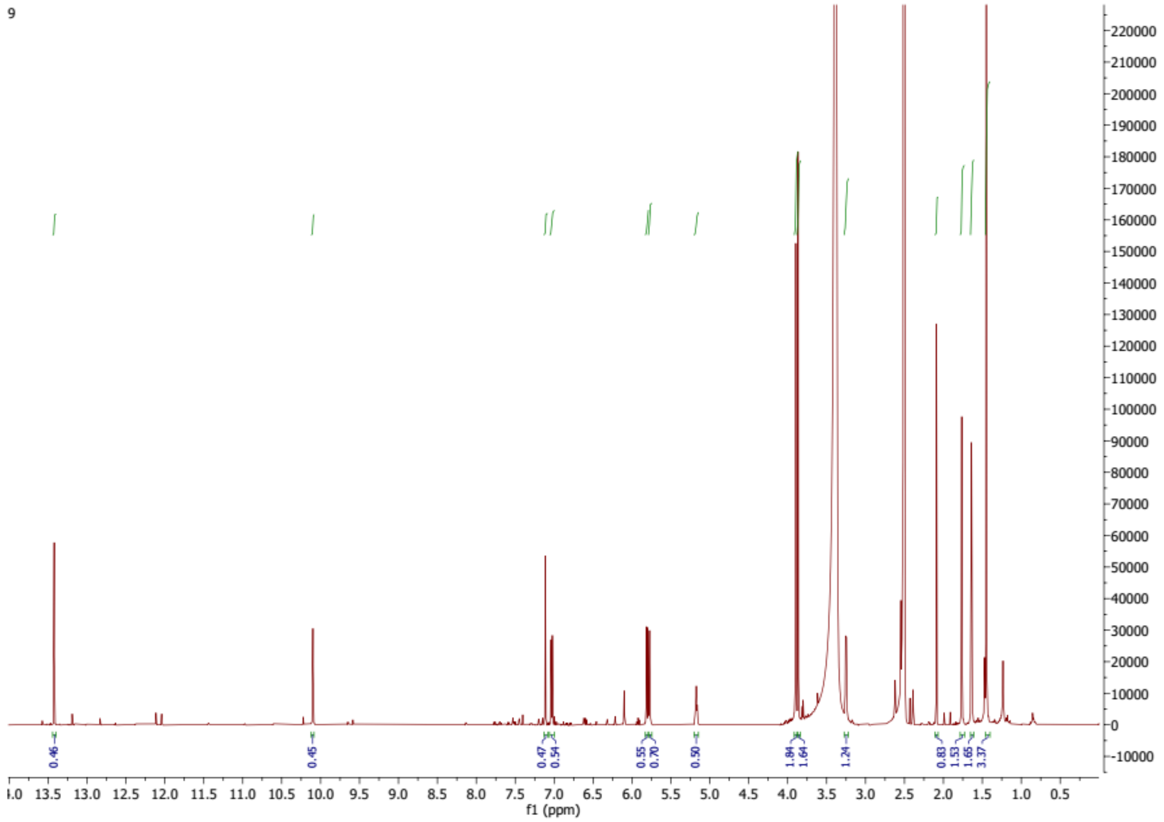

Figure S39: ^{1}H NMR (DMSO-d_6, 500 MHz) spectrum of compound **9**

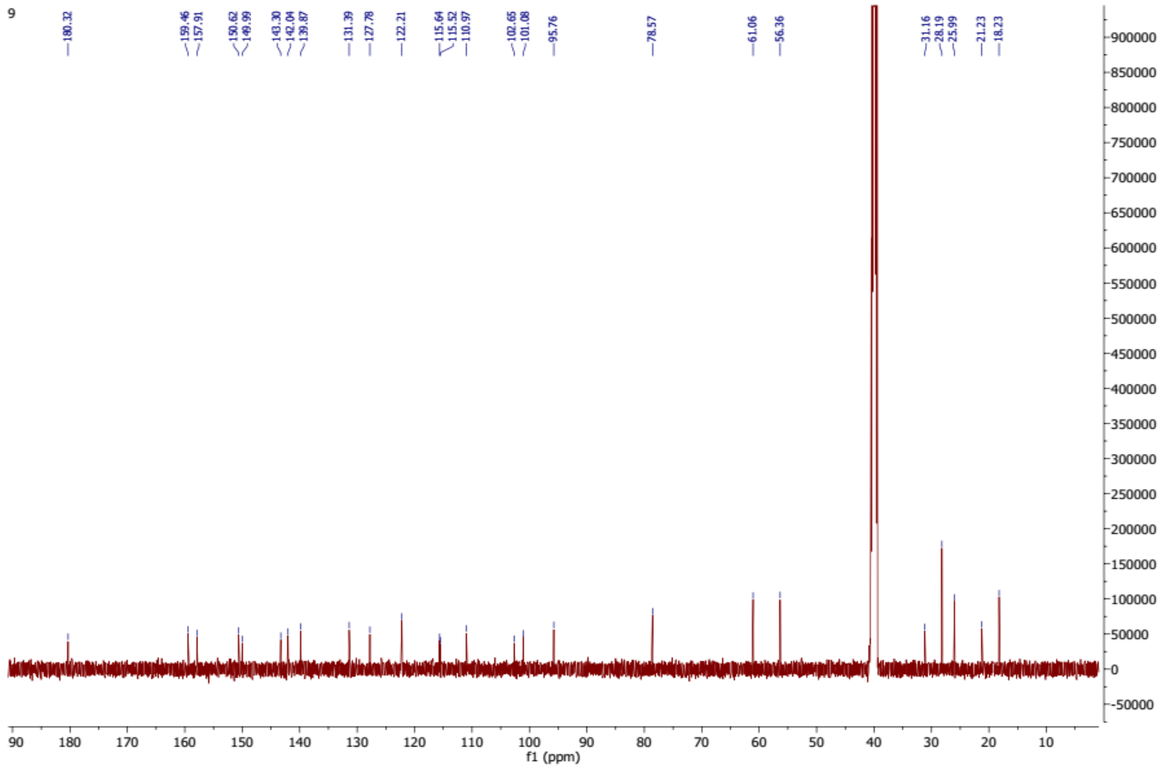

Figure S40: ^{13}C NMR (DMSO-d_6, 125 MHz) spectrum of compound **9**

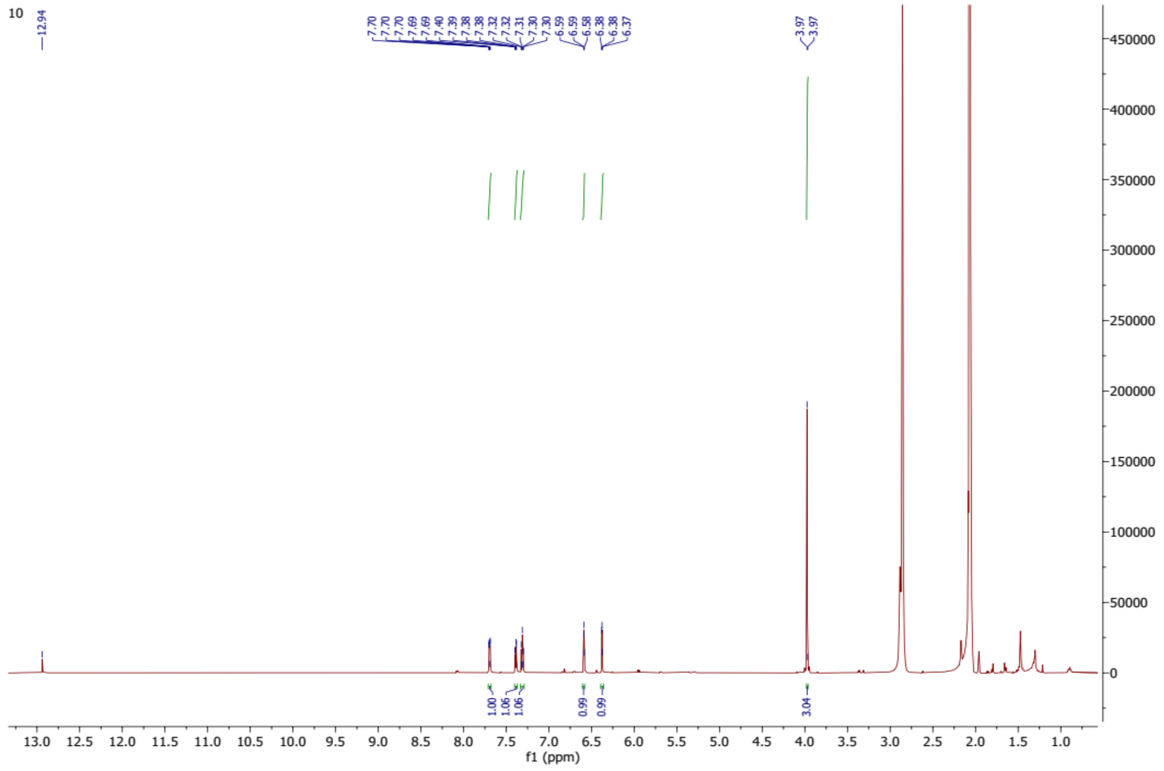

Figure S41: ^{1}H NMR (Acetone, 500 MHz) spectrum of compound **10**

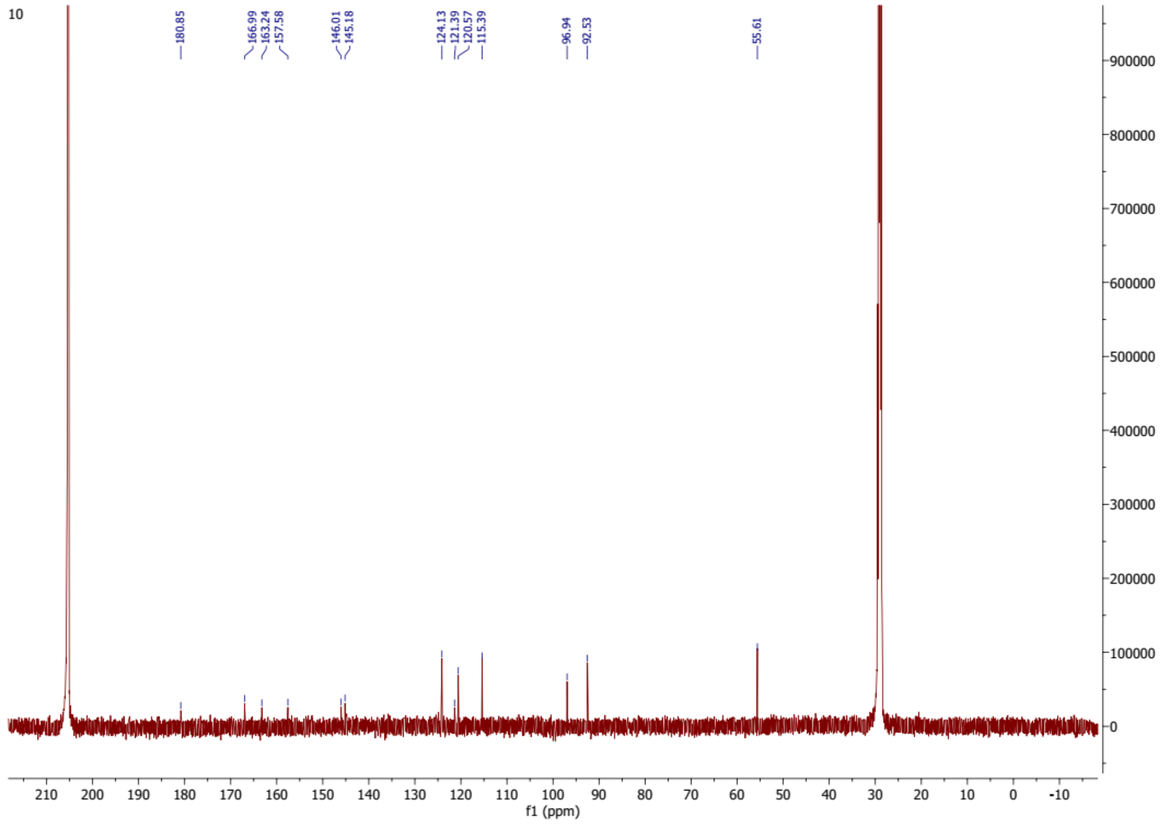

Figure S42: ^{13}C NMR (Acetone, 125 MHz) spectrum of compound **10**

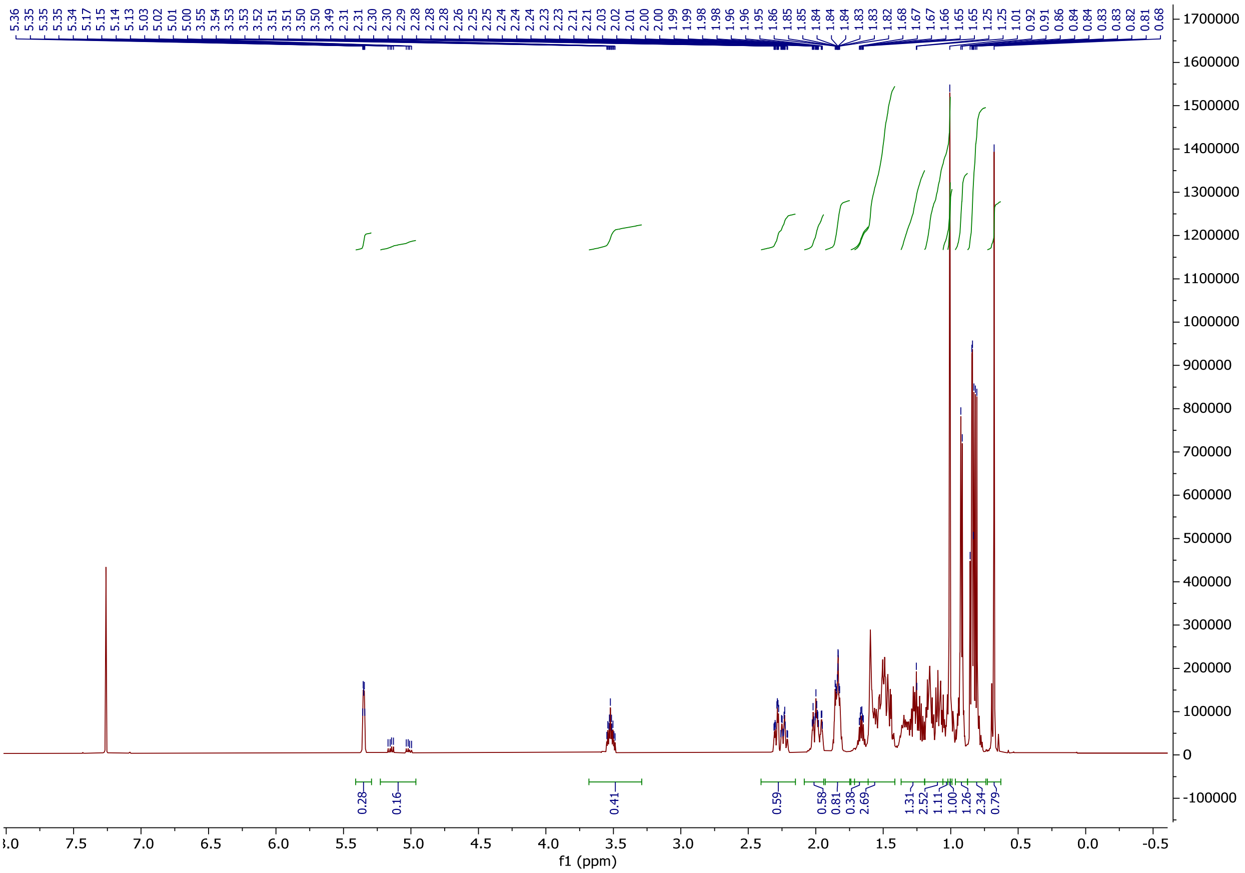

Figure S43: ^{1}H NMR ($CDCl_3$, 600 MHz) spectrum of compound **11+12**

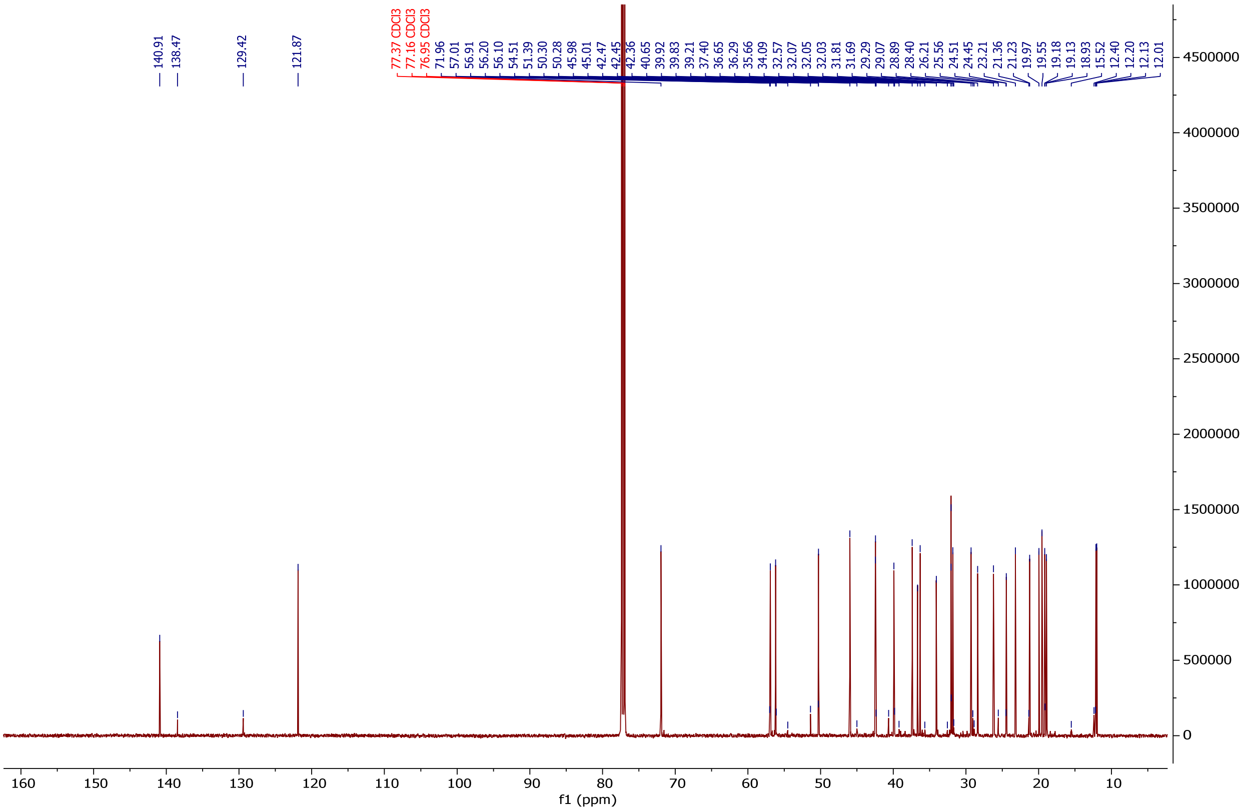

Figure S44: ^{13}C NMR ($CDCl_3$, 150 MHz) spectrum of compound **11+12**

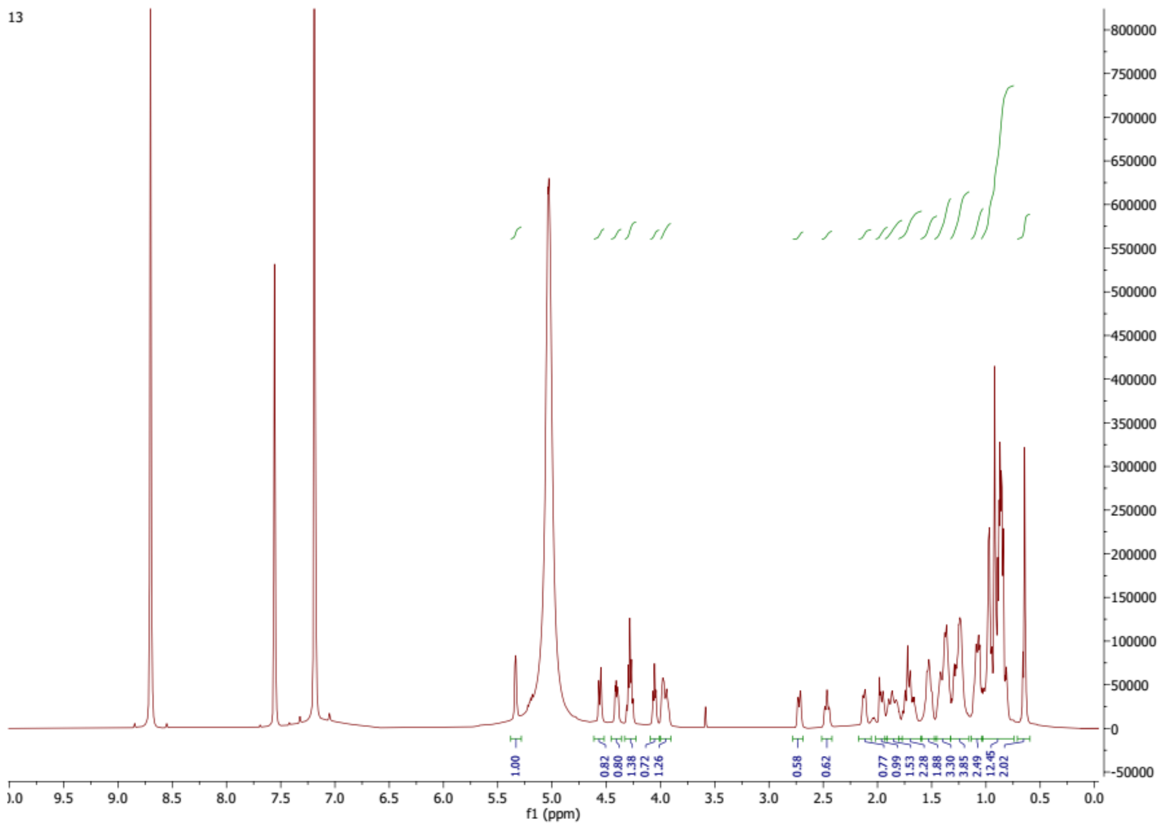

Figure S45: ^{1}H NMR (Pyridine-d_5, 600 MHz) spectrum of compound **13**

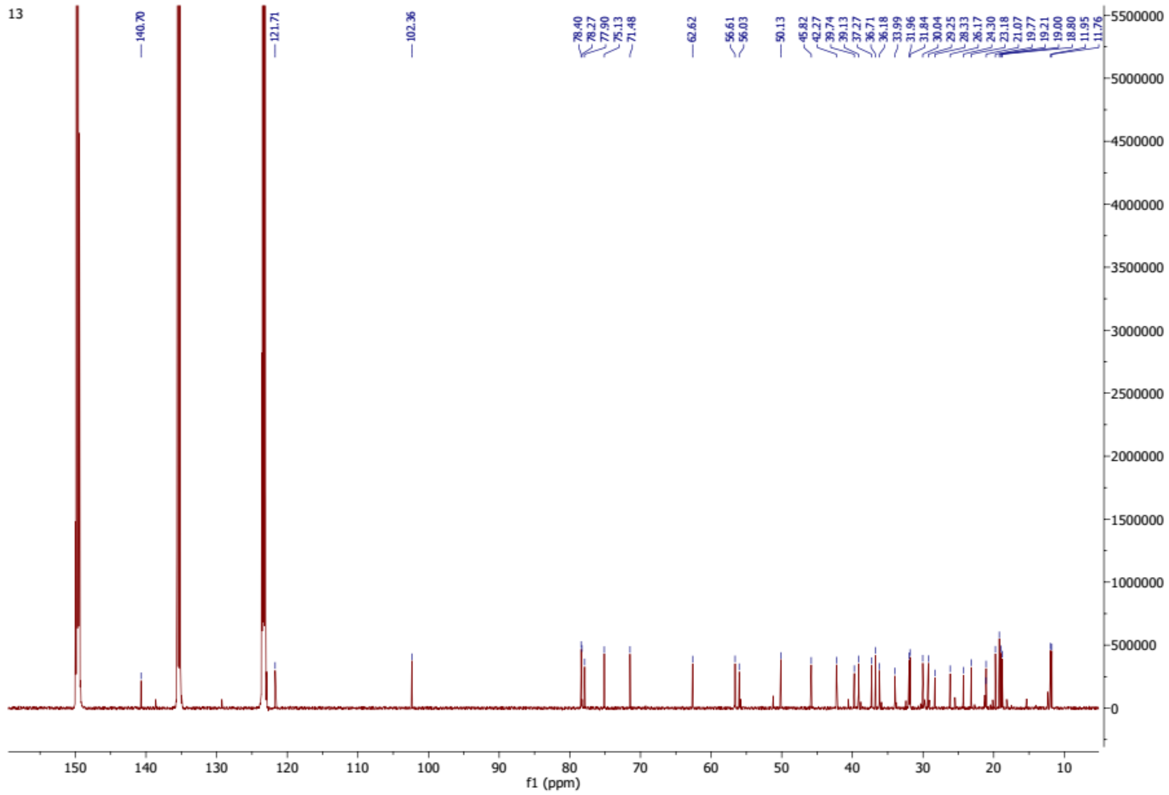

Figure S46: ^{13}C NMR (Pyridine-d_5, 150 MHz) spectrum of compound **13**

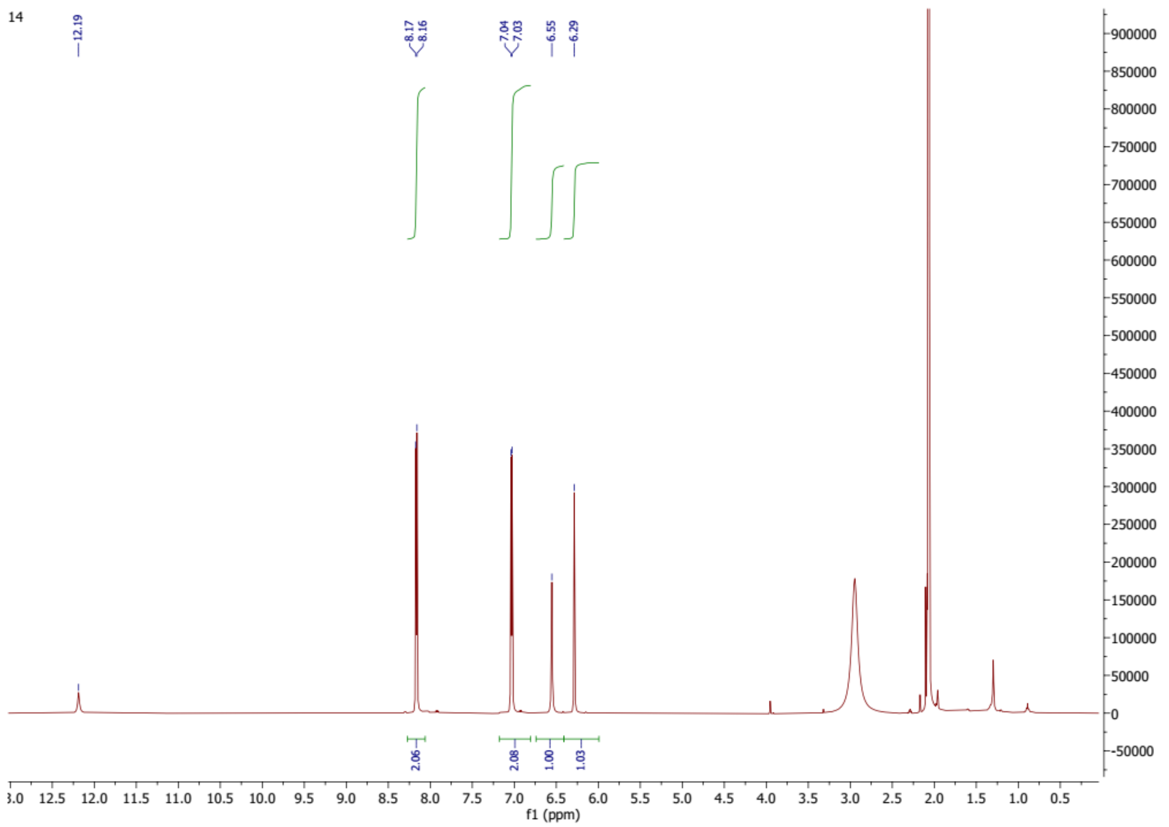

Figure S47: ^{1}H NMR (Acetone, 600 MHz) spectrum of compound **14**

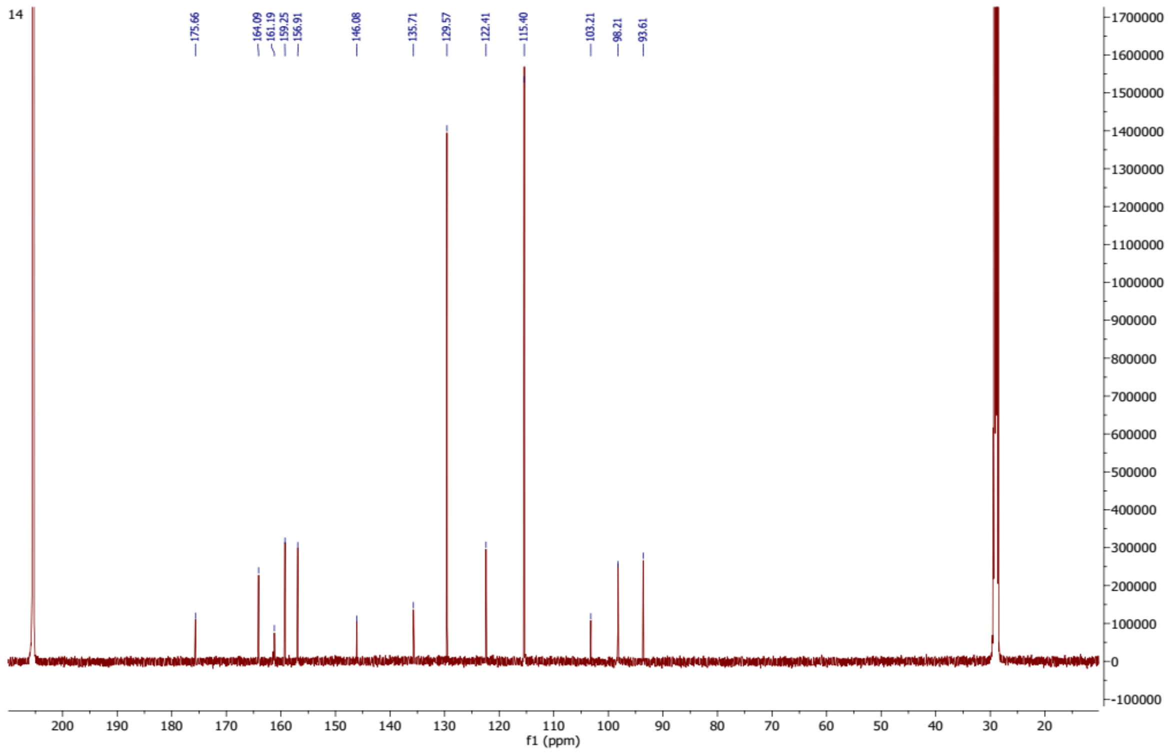

Figure S48: ^{13}C NMR (Acetone, 150 MHz) spectrum of compound **14**

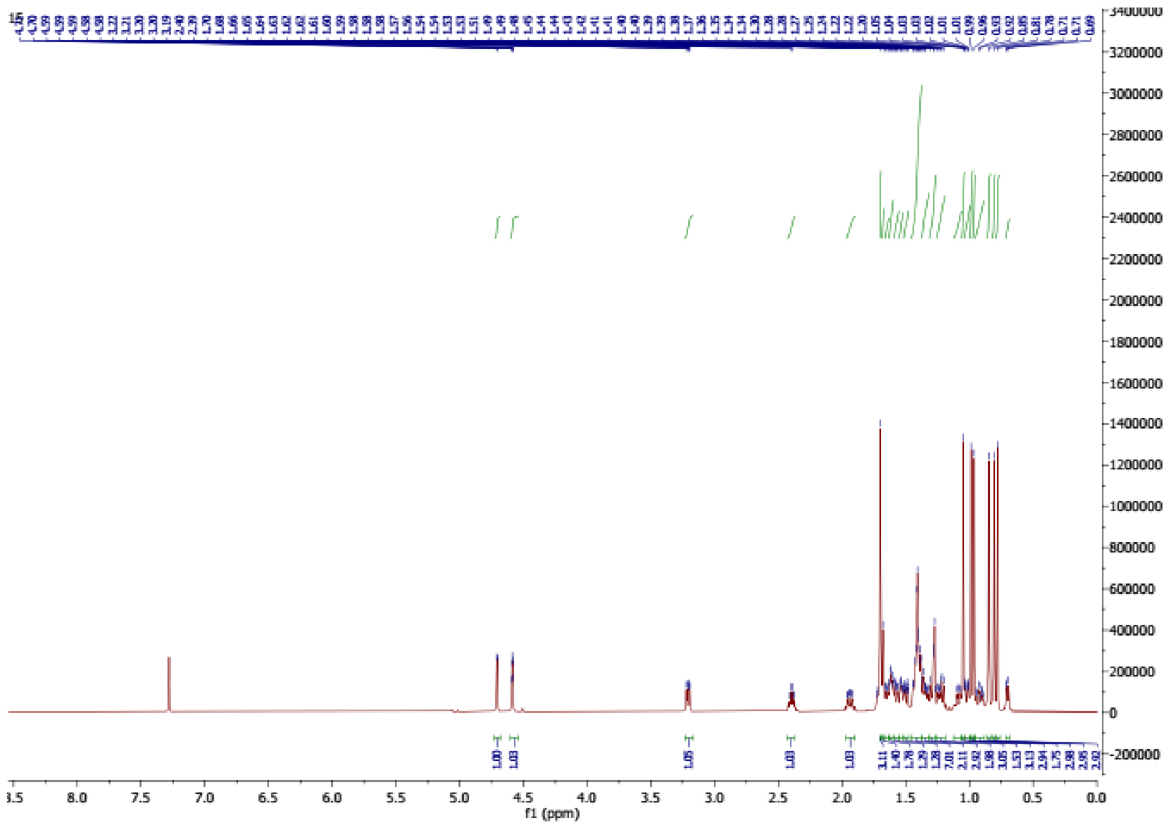

Figure S49: ^{1}H NMR ($CDCl_3$, 600 MHz) spectrum of compound **15**

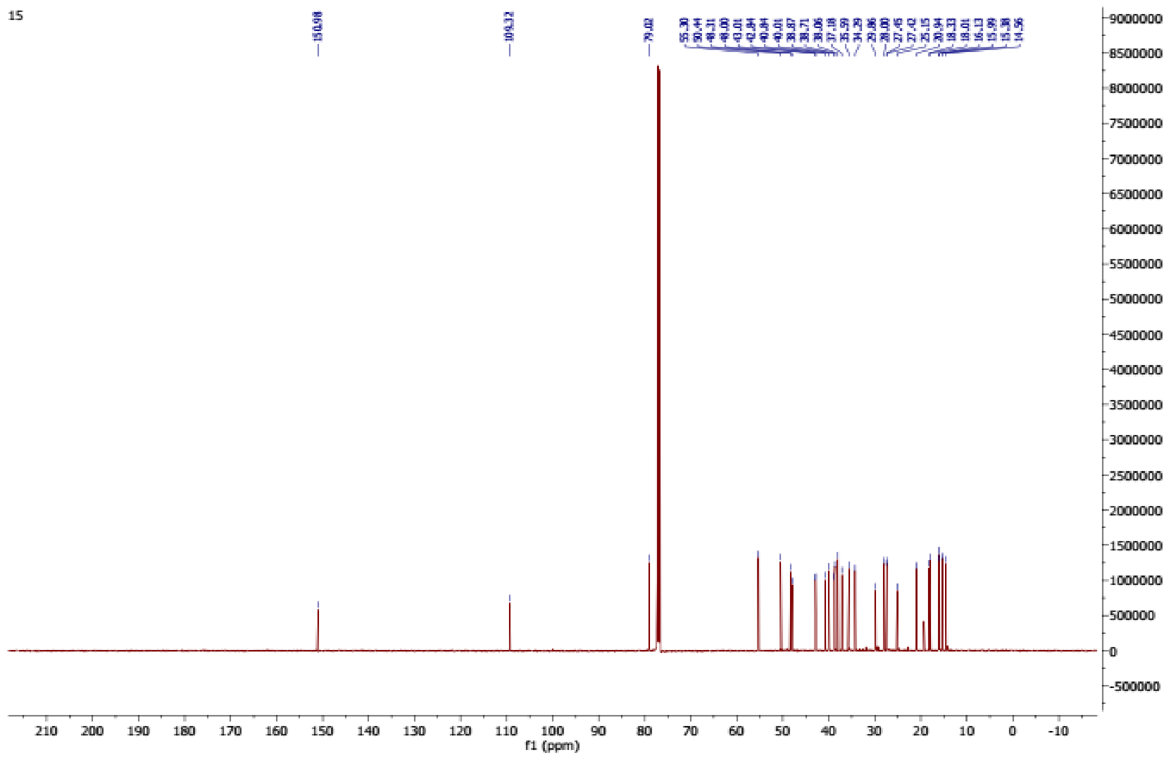

Figure S50: ^{13}C NMR ($CDCl_3$, 150 MHz) spectrum of compound **15**
